# Untargeted Metabolomics and Targeted Quantitative Analysis of Temporal and Spatial Variations in Specialized Metabolites Accumulation in *Poria cocos* (Schw.) Wolf (Fushen)

**DOI:** 10.3389/fpls.2021.713490

**Published:** 2021-09-21

**Authors:** Mei Yang, Yujiao Zhao, Yuejian Qin, Rui Xu, Zhengyang Yang, Huasheng Peng

**Affiliations:** ^1^School of Pharmacy, Anhui University of Chinese Medicine, Hefei, China; ^2^National Resource Center for Chinese Materia Medica, China Academy of Chinese Medical Sciences, Beijing, China; ^3^Research Unit of DAO-DI Herbs, Chinese Academy of Medical Sciences, Beijing, China

**Keywords:** Fushen, saprophytic fungus, UPLC-Q-TOF/MS, untargeted metabolomics, quantitative analysis, temporal and spatial distributions

## Abstract

*Poria cocos* (Schw.) Wolf is a saprophytic fungus that grows around the roots of old, dead pine trees. Fushen, derived from the sclerotium of *P. cocos* but also containing a young host pine root, has been widely used as a medicine and food in China, Japan, Korea, Southeast Asian countries, and some European countries. However, the compound variations at the different growth periods and in the different parts of Fushen have not previously been investigated. In this study, an untargeted metabolomics approach based on ultra-performance liquid chromatography-quadrupole time-of-flight mass spectrometry (UPLC-Q/TOF-MS) and targeted quantitative analysis was utilized to characterize the temporal and spatial variations in the accumulation of specialized metabolites in Fushen. There were 119 specialized metabolites tentatively identified using the UPLC-Q/TOF-MS. The nine growth periods of Fushen were divided into four groups using partial least squares discrimination analysis (PLS-DA). Four different parts of the Fushen [fulingpi (FP), the outside of baifuling (BO), the inside of baifuling (BI), and fushenmu (FM)] were clearly discriminated using a PLS-DA and orthogonal partial least squares discrimination analysis (OPLS-DA). Markers for the different growth periods and parts of Fushen were also screened. In addition, the quantitative method was successfully applied to simultaneously determine 13 major triterpenoid acids in the nine growth periods and four parts. The quantitative results indicated that the samples in January, March, and April, i.e., the late growth period, had the highest content levels for the 13 triterpenoid acids. The pachymic acid, dehydropachymic acid, and dehydrotumulosic acid contents in the FM were higher than those in other three parts in March, whereas the poricoic acid B, poricoic acid A, polyporenic acid C, dehydrotratrametenolic acid, dehydroeburicoic acid, and eburicoic acid in FP were higher beginning in October. These findings reveal characteristics in temporal and spatial distribution of specialized metabolites in Fushen and provide guidance for the identification of harvesting times and for further quality evaluations.

## Introduction

*Poria cocos* (Schw.) Wolf is a saprophytic fungus that grows around the roots of old, dead pine trees (Committee for the Pharmacopoeia of PR China, 2020). However, some taxonomists have proposed that the synonym *Pachyma hoelen* should be used rather than *P. cocos* as the name of the Chinese cultivar (Wu et al., 2020a). *P. cocos* has been widely used as a medicine and food in China, Japan, Korea, Southeast Asia, and some European countries (Zhu et al., 2020). In traditional Chinese medicine, it is used to treat edema with less urine, restlessness, and insomnia with palpitations (Committee for the Pharmacopoeia of PR China, 2020). Recently, pharmacological studies have revealed that *P. cocos* has a diverse array of activities, such as antitumor (Jiang and Fan, 2021), anti-inflammatory (Li et al., 2021), antihyperglycemic (Li et al., 2011), antioxidant (Wu et al., 2020b), immune-modulating (Chao et al., 2021), liver protection (Cheng et al., 2021b), diuresis (Liang et al., 2021), and sedative-hypnotic (Shah et al., 2014). Additionally, *P. cocos* has been shown to contain the triterpenoid acids, polysaccharides, sterols, and other active ingredients. Among them, triterpenoid acids are the main specialized metabolites, and, to date, more than 100 have been identified (Feng et al., 2018).

*Poria cocos* is an annual fungus. Its sclerotium, specifically, is a well-known traditional Chinese medicine, called Fushen or Fuling. Fushen refers to the sclerotium of *P. cocos* when it contains a young host pine root, which is called the fushenmu. The Fuling, however, refers to the sclerotium of *P. cocos* when it does not contain a young host pine root. However, the young host pine roots in Fushen are used in medicine due to the depletion of wild resources, difficulties in cultivation techniques, and increasing market demands for Fushen, and, consequently, the price of Fushen is much higher than that of Fuling. Market surveys and origin investigations have revealed that the earlier Fushen is harvested, the more subsequent profits are increased. Fushen is generally cultivated beginning in June, with harvesting occurring 5–10 months after cultivation. However, there is a close relationship between the harvesting date and quality of traditional Chinese medicines. There are also differences in the effective compounds during different growth periods (Gao, 2015). Early harvesting of Fushen affects the safety and effectiveness of the derived clinical medications. However, at present, the differences in the compounds of Fushen at different growth periods have not been investigated.

It is well-known that the quality and therapeutic effects of Chinese medicines are closely related with their specialized metabolites, which are mainly accumulated in different parts and different growth periods (Hu, 2014; Pusztahelyi et al., 2015; Bazargani et al., 2021; Zhao et al., 2021). The epidermis, middle part, and inner part of Fushen have previously been studied, and it was found that there were significant differences in the specialized metabolites among the three parts (Wang et al., 2015; Zhu et al., 2018b). Moreover, the different parts of the Fushen, from the outside to the inside, have different pharmacological effects and clinical applications (Zhu et al., 2020). However, the dynamic changes in specialized metabolites in these parts during different growth periods have not yet been studied.

In this study, we applied an untargeted metabolomics and targeted quantitative approach to the temporal and spatial variations in the accumulation of specialized metabolites in 115 Fushen samples. Qualitative characterization of triterpenoid acids in the four parts of the Fushen at nine growth periods was performed using ultra-performance liquid chromatography-quadrupole time-of-flight mass spectrometry (UPLC-Q/TOF-MS) and multivariate statistical analysis methods, including hierarchical clustering analysis heat map, principal component analysis (PCA), partial least squares discriminant analysis (PLS-DA), and orthogonal projections to latent structures discriminant analysis (OPLS-DA). In addition, the contents of 13 major triterpenoid acids were simultaneously determined by UPLC to explore the quantitative differences in Fushen. Studying the dynamic accumulation and distribution of specialized metabolites in Fushen during different growth periods is of theoretical importance. These findings provide a reference for growth regularity, harvest time, and further quality evaluations of Fushen.

## Materials and Methods

### Plant Materials

**Figure 1:**
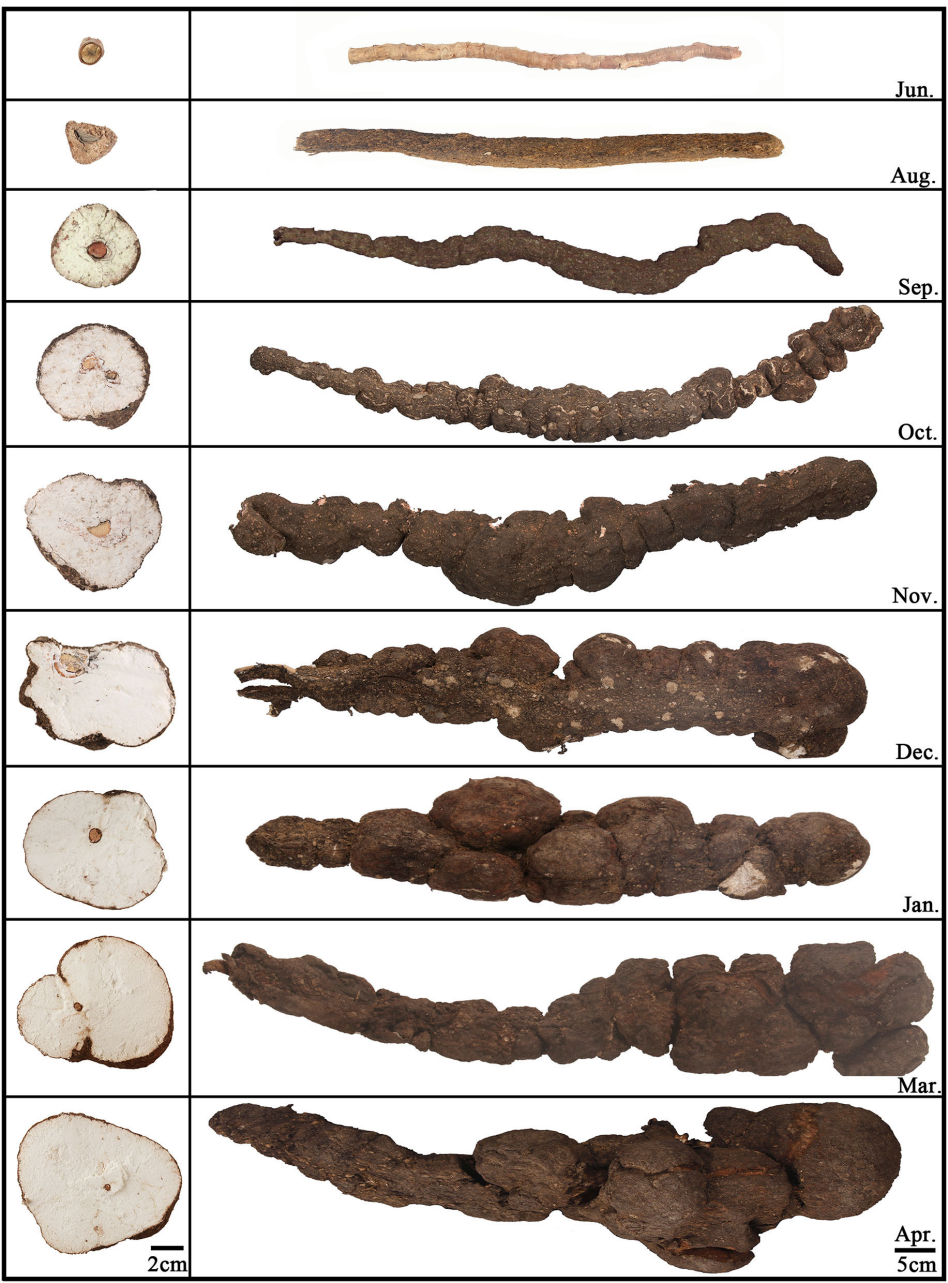
Morphological characteristics of nine growth periods of Fushen.

Twenty-six Fushen samples from nine growth periods and 89 samples from all parts of Fushen at nine growth periods were used in this study and were collected from June 2019 to April 2020 in Anhui province, China. Detailed information on the samples is provided in Supplementary Tables 1, 2. Three samples were taken in the first eight growth periods and two samples in the ninth growth period. There were three samples taken from each part in the first eight growth periods and two samples from each part during the ninth growth period. All Fushen samples were authenticated by Prof. Huasheng Peng from the National Resource Center for Chinese Materia Medica, China Academy of Chinese Medical Sciences. The samples for the nine growth periods are shown in Figure 1, and the environment of the field is shown in Supplementary Figure 1. The first growth period was collected in June, which had not been inoculated with mycelia. The rest of the samples were collected at intervals of 30–60 days.

Based on the morphological characteristics, the Fushen could be classified into four parts, from the outside to the inside, as fulingpi (the epidermis, abbreviated as FP), outside of the baifuling (the white part near the epidermis, abbreviated as BO), inside of the baifuling (the white part near the young host pine root, abbreviated as BI), and fushenmu (the young host pine root in the inner part, abbreviated as FM) (Supplementary Figure 2). The samples taken in June were separated into one part (FM); the samples taken in August were separated into two parts (FP, FM); the samples taken in September, October, November, December, January, March, and April were separated into four parts (FP, BO, BI, and FM). All the crude and dissected samples were ground into a powder (80 mesh) and stored under dry conditions at 25°C before analysis.

### Chemicals and Reagents

Based on the specific makers identified in this study and the active compounds of Fushen that have been reported in the literature, 13 triterpenoid acids were selected for quality control compounds (Sun et al., 2019; Deng et al., 2020). The 16α-hydroxydehydrotrametenolic acid, poricoic acid B, dehydrotumulosic acid, poricoic acid A, polyporenic acid C,3-o-acetyl-16α-hydroxydehydrotrametenolic acid, dehydropachymic acid, dehydroeburicoic acid, dehydrotrametenolic acid, 16α-hydroxytrametenolic acid, 3-o-acetyl-16α-hydroxytrametenolic acid, pachymic acid, and eburicoic acid were purchased from Chengdu Push Bio-technology Co., Ltd. (Chengdu, China), Jiangsu Yongjian Pharmaceutical Technology Co., Ltd. (Jiangsu, China), Baoji Herbest Bio-technology Co., Ltd. (Shanxi, China), and Chengdu Desite Bio-technology Co., Ltd. (Chengdu, China). The purity of each standard was above 98%, except for 3-o-acetyl-16α-hydroxydehydrotrametenolic acid (purity ≥95%). The chemical structures for all reference standards are shown in Supplementary Figure 3. HPLC-grade acetonitrile and methanol were supplied by Tedia (Fairfield, OH, USA), and formic acid [Liquid chromatography-mass spectrometry (LC-MS) grade] was purchased from Aladdin (CA, USA). Ultra-high purity water was obtained using a Milli-Q system (Millipore, MA, USA). Ethanol was purchased from Tedia (Fairfield, OH, USA).

### Standard and Sample Preparation

The standard compounds were accurately weighed and individually dissolved in methanol to prepare standard stock solutions in a range of concentrations from 1 to 2 mg/ml. The stock solutions were diluted with methanol to construct calibration curves. Both standard solutions were stored at 4°C until further analysis.

Samples (0.5 g) for the UPLC-Q/TOF-MS analysis were accurately weighed into 50-ml conical flasks and extracted with 20-ml methanol by sonication (40 kHz, 100 W) at 60°C for 60 min. Finally, solutions were filtered through 0.22-μm Millipore filters and stored at 4°C until analysis.

Samples for UPLC analysis were accurately weighed (1 g) into 50-ml conical flasks and extracted with 40-ml ethanol by sonication (40 kHz, 100 W) at 60°C for 60 min. The extracts were then shaken and filtered. The filtered fluid was then recovered and dried. The residues were then dissolved in methanol (5 ml). Finally, solutions were filtered through 0.22-μm Millipore filters and stored at 4°C until use in the analysis.

### UPLC-Q/TOF-MS Conditions for Untargeted Metabolomics

Ultra-performance liquid chromatography was performed on a Waters ACQUITY UPLC system (Waters Crop., Milford, MA, USA) with an ACQUITY UPLC BEH C18 column (2.1 × 100 mm, 1.7 μm, Waters) and a BEH C18 VanGuard Pre-column (2.1 × 5 mm, 1.7 μm, Waters) at 35°C. The mobile phase used was a mixture of acetonitrile (A) and 0.1% formic acid in water (B). The gradient elution was as follows: 0–2 min, 40–55% A; 2–25 min, 55%−80% A; 25–27 min, 80–90% A; 27–28 min, 90–90% A; 28–30 min, 90–46% A; 30–35 min, 46–46% A. The flow rate was set at 0.2 ml/min, and the injection volume was 1 μl.

Mass spectrometry detection was performed on a Waters Xevo G2-S Q-TOF mass spectrometer (Waters Corp, Milford, MA, USA), equipped with an electrospray ionization (ESI) source. The MS data were acquired using (ESI) in the negative and positive ionization modes with a scan range of *m*/*z* 50–1,200 Da. Mass spectrometry analysis was performed under the following optimized conditions: capillary voltage, 3 kV; desolvation gas flow rate, 600 L/h; cone gas flow rate, 50 L/h; desolvation gas temperature, 350°C; and ion source temperature, 120°C. The collision energies were set at 20 and 40 eV. Mass accuracy and reproducibility were maintained using a lock spray. Leucine enkephalin was used as a reference lock mass during data acquisition for online calibration to ensure mass accuracy. All MS data were analyzed using the MassLynx software (version 4.1, Waters Corp, Milford, MA, USA).

### UPLC Conditions for Quantitative Analysis of Triterpenoid Acids

Ultra-performance liquid chromatography was performed on a Waters ACQUITY UPLC system (Waters Crop., Milford, MA, USA) with a PAD detector, sample manager-FIN, quaternary solvent manager, and a column oven. Chromatographic separation was performed using an ACQUITY UPLC BEH C18 column (2.1 × 100 mm, 1.7 μm, Waters) and a BEH C18 VanGuard pre-column (2.1 × 5 mm, 1.7 μm, Waters) at 35°C. The mobile phase used was a mixture of acetonitrile (A) and 0.1% formic acid in water (B). The gradient elution was as follows: 0–3 min, 60–60% A; 3–15 min, 60–80% A; 15–17 min, 80–90% A; 17–19 min, 90–90% A; 19–21 min, 90–60% A. The flow rate was set at 0.2 ml/min, and the injection volume was 2 μl. PDA detection was set at 210 and 245 nm.

### Validation of the Quantitative Method

A series of standard solutions of appropriate concentrations was prepared for the construction of calibration curves. The limits of detections (LODs) and limits of quantification (LOQs) for the analytes were determined at signal-to-noise ratios (S/N) of 3 and 10, respectively. The intra-day and inter-day precisions were performed by analyzing the mixed standard solutions in six replicates within a single day and six consecutive days, respectively. Repeatability was evaluated using six replicates from the same sample (No. 1). For stability assessments, the sample solution (No. 1) was analyzed at 0, 2, 4, 8, 12, 16, 24, 36, and 48 h. The accuracy of the developed method was evaluated using a recovery test at different levels (80, 100, and 120%). It was performed by adding known amounts of standards into the same sample (No. 1) and calculated using the equation: recovery (%) = (total detected amount – original amount)/spiked amount × 100%. Variations in precision, repeatability, stability, and accuracy were expressed as relative standard deviation (RSD).

### Data Preprocessing and Statistical Analysis

The metabolites acquired by UPLC-Q/TOF-MS-based metabolomics profiling were analyzed using MassLynx software (version 4.1, Waters Corp., Milford, MA, USA). Peak detection, alignment, and filtering of the raw MS data were performed using the MassLynx software (Chang et al., 2017). The method parameters were set as follows: retention times (*tR*) of 1–30 min, mass tolerance of 0.05 Da, the noise elimination level of 10.00, the intensity threshold of 300 counts, and the XIC window of 0.02 Da. Isotopic peaks were excluded from the analysis. For peak integration, the parameters of peak width at 5% height and peak-to-peak baseline noise were automatically calculated. The ion intensities of each detected peak were normalized by the sum of the peak intensities within that sample using MarkerLynx. Then, specialized metabolites were definitely or tentatively identified by comparison with standard compounds, accurate mass, fragmentation patterns, and a database from literature. These reference standards with MS data and retention times were used to derive the structural information with confidence level 1 (CL1), while metabolite features without reference standards were putatively identified at confidence level 2 (CL2) according to the metabolomics standards initiative (Schymanski et al., 2014). Finally, the sample name, peak number, and ion intensity datasets were tabulated and imported into SIMCA 14 software (Umetrics, Malmo, Sweden) for multivariate statistical analysis, including unsupervised PCA, supervised PLS-DA, and supervised OPLS-DA. A hierarchical clustering analysis heat map was performed using TB tools. In addition, line charts were analyzed using GraphPad Prism 8.0 software (GraphPad, San Diego, USA).

## Results

### Identification of Specialized Metabolites in Fushen by UPLC-Q/TOF-MS

**Figure 2:**
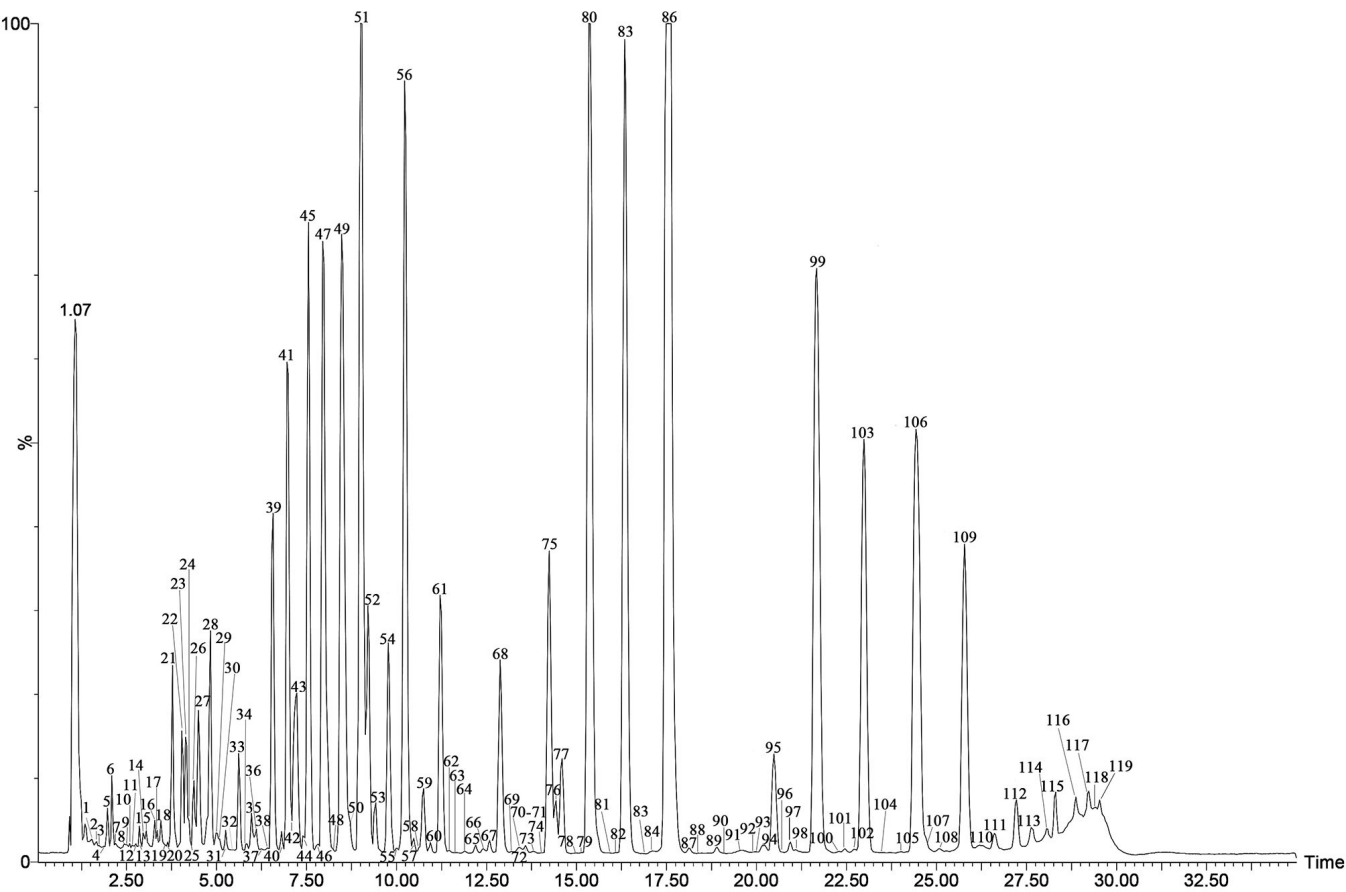
The TIC chromatogram of the QC of Fushen in ESI^−^.

The specialized metabolites of Fushen were analyzed using the UPLC-Q/TOF-MS method in both positive and negative ion modes. A representative total ion chromatogram (TIC) of Fushen is shown in Figure 2. A total of 119 specialized metabolites were definitely or tentatively identified in Fushen by comparison with standard compounds, accurate mass, fragmentation patterns, and databases from the literature (Zheng and Yang, 2008; Akihisa et al., 2009; Li, 2013; Wang, 2014, 2016; Feng et al., 2018; Zhu et al., 2018b; Chen et al., 2019; Zou et al., 2019; Deng et al., 2020). The detected and identified compounds were numbered according to their elution order, and the characterization of the specialized metabolites identified in the negative ion mode is shown in Table 1, and those identified in the positive ion mode are shown in Supplementary Table 3.

**Table 1:** Characterization of the specialized metabolites in Fushen using UPLC-Q/TOF-MS in ESI^−^.

**No**.	***tR* (min)**	**Molecular formula**	**Adduct ion (mass accuracy, ppm)**	**MS/MS Fragmentation (mass accuracy, ppm <10)**	**Identification**	**References**
1	1.53	C_29_H_38_O_14_	609.2192[M-H]^−^(1.5)	347.1870	Unknown	
2	1.67	C_20_H_26_O_5_	345.1689[M-H]^−^(−3.8)		Unknown	Zhu et al., 2018b
3	1.73	C_20_H_30_O_5_	349.1995[M-H]^−^(−5.7) 395.2063[M-H+HCOOH]^−^(−1.8)	265.1440	Pregn-7-ene-2β,3α,15α,20(S)-tetrol	Deng et al., 2020
4	1.87	C_20_H_32_O_5_	351.2140[M-H]^−^(−8.8) 397.2187[M-H+HCOOH]^−^(−9.8)	317.1723	Unknown	
5	1.97	C_20_H_28_O_4_	331.1883[M-H]^−^(−7.9)		Unknown	Zhu et al., 2018b
6	2.09	C_20_H_28_O_4_	331.1883[M-H]^−^(−7.9)		Unknown	Zhu et al., 2018b
7	2.20	C_17_H_24_O_4_	291.1574[M-H]^−^(−7.6)		Unknown	Zhu et al., 2018b
8	2.43	C_20_H_26_O_4_	329.1765[M-H]^−^(3.6)	251.1639	Unknown	Zhu et al., 2018b
9	2.52	C_20_H_28_O_4_	331.1883[M-H]^−^(−7.9)		Unknown	Zhu et al., 2018b
10	2.58	C_26_H_46_O_9_	501.3102[M-H]^−^(7.6)		Unknown	
11	2.65	C_20_H_32_O_4_	335.2229[M-H]^−^(2.1)		Unknown	
12	2.75	C_20_H_32_O_5_	351.2140[M-H]^−^(−8.8) 397.2187[M-H+HCOOH]^−^(−9.8)	317.1723	Unknown	
13	2.86	C_20_H_28_O_4_	331.1883[M-H]^−^(−7.9)		Unknown	Zhu et al., 2018b
14	2.96	C_20_H_26_O_4_	329.1765[M-H]^−^(3.6)	251.1639	Unknown	Zhu et al., 2018b
15	3.05	C_30_H_44_O_6_	499.3052[M-H]^−^(−1.6) 545.3123[M-H+HCOOH]^−^(1.7)	481.2930[M-H-H_2_O]^−^ 425.2697[M-H-C_2_H_5_COOH]^−^ 467.2764 327.2152	Poricoic acid E/Poricoic acid E isomer	Feng et al., 2018; Zhu et al., 2018b
16	3.28	C_30_H_46_O_5_	485.3242[M-H]^−^(−5.2) 531.3289[M-H+HCOOH]^−^(−6.2)	441.2691[M-H-CO_2_]^−^ 423.3255[M-H-CO_2_-H_2_O]^−^ 471.3108	Poricoic acid G	Zhu et al., 2018b Zou et al., 2019
17	3.35	C_31_H_48_O_7_	531.3336[M-H]^−^(2.6)	513.3320[M-H-H_2_O]^−^ 485.3242[M-H-HCOOH]^−^	Poricoic Acid K	Chen et al., 2019
18	3.45	C_31_H_46_O_6_	513.3223[M-H]^−^(1.4) 531.3224[M-H+HCOOH]^−^(−8.4)	497.2903[M-H-CH_4_]^−^ 501.3239 331.1920	Poricoic acid D/F	Zou et al., 2019
19	3.57	C_31_H_48_O_5_	499.3418[M-H]^−^(−1.0) 545.3458[M-H+HCOOH]^−^(−3.7)	483.3095[M-H-CH_4_]^−^	Poricoic acid GM	Zou et al., 2019
20	3.64	C_30_H_44_O_6_	499.3052[M-H]^−^(−1.6)	481.2930[M-H-H_2_O]^−^ 425.2697[M-H-C_2_H_5_COOH]^−^ 453.3100 437.2287	Poricoic acid E/Poricoic acid E isomer	Feng et al., 2018; Zhu et al., 2018b
21	3.78	C_31_H_48_O_5_	499.3418[M-H]^−^(−1.0) 545.3505[M-H+HCOOH]^−^(5.0) 999.6895[2M-H]^−^(−3.0)	481.3334[M-H-H_2_O]^−^ 419.2980[M-H-HCOOH-CH_4_-H_2_O]^−^ 437.3315	29-Hydroxydehydrotumulosic acid	Zou et al., 2019
22	4.03	C_31_H_46_O_6_	513.3223[M-H]^−^(1.4) 559.3224[M-H+HCOOH]^−^(5.0)	439.2852[M-H-C_2_H_5_COOH]^−^ 487.3416 441.2992	5α,3β-Peroxy-dehydrotumulosicacid/ 5α,8α-Peroxy-dehydrotumulosic acid	Wang, 2014; Zhu et al., 2018b Zou et al., 2019
23	4.17	C_31_H_46_O_6_	513.3223[M-H]^−^(1.4) 559.3224[M-H+HCOOH]^−^(5.0)	439.2809[M-H-C_2_H_5_COOH]^−^ 487.3416 441.2992	5α,3β-Peroxy-dehydrotumulosicacid/ 5α,8α-Peroxy-dehydrotumulosic acid	Wang, 2014; Zhu et al., 2018b; Zou et al., 2019
24	4.24	C_30_H_46_O_5_	485.3287[M-H]^−^(4.1)	467.3120[M-H-H_2_O]^−^ 449.2364	16α,27-Dihydroxy-dehydrotrametenoic acid	Zhu et al., 2018b; Zou et al., 2019
25	4.30	C_30_H_48_O_5_	487.3416[M-H]^−^(−1.4) 533.3452[M-H+HCOOH]^−^(−4.9)	451.3192[M-H-2H_2_O]^−^ 441.2261 367.2089	3α,16α,25-Trihydroxylanosta-8,24-dien-21-oic acid	Zou et al., 2019
26	4.37	C_31_H_48_O_5_	499.3418[M-H]^−^(−1.0) 545.3458[M-H+HCOOH]^−^(−3.7)	483.3095[M-H-CH_4_]^−^ 481.3334[M-H-H_2_O]^−^ 437.3420[M-H-CO_2_-H_2_O]^−^	16α,25-Dihydroxyeburiconic acid/ 25-Hydroxy-3-epi-dehydrotumulosic acid	Akihisa et al., 2009; Zou et al., 2019
27	4.49	C_31_H_46_O_5_	497.3270[M-H]^−^(0.6) 543.3356[M-H]^−^(6.3) 995.6573[2M-H]^−^(−3.9)	481.2930[M-H-CH_4_]^−^ 419.2980[M-H-H_2_O-CO_2_-CH_4_]^−^ 435.3310[M-H_2_O-CO_2_]^−^	25-Hydroxyporicoic acid C/25-Hydroxypolyporenic acid C/ 6-Hydroxypolyporenic acid C/Poricoic acid BM/ 3-Oxo-6,16α-dihydroxy-lanosta-7,9(11),24(31)-trien-21-oic acid	Li, 2013; Wang, 2016; Zhu et al., 2018b
28	4.83	C_31_H_48_O_6_	515.3367[M-H]^−^(−1.2) 561.3425[M-H+HCOOH]^−^(−0.4) 1031.6835[2M-H]^−^(1.1)	497.3270[M-H-H_2_O]^−^ 499.3418 423.2843	25-Hydroxyporicoic acid H	Zhu et al., 2018b
29	4.99	C_31_H_48_O_5_	499.3418[M-H]^−^(−1.0) 545.3458, [M-H+HCOOH]^−^(−3.7)	481.3334[M-H-H_2_O]^−^ 437.3420[M-H-CO_2_-H_2_O]^−^	15α-Hydroxydehydrotumulosic acid	Zou et al., 2019
30	5.08	C_32_H_50_O_5_	513.3548[M-H]^−^(−6.2) 559.3611[M-H+HCOOH]^−^(−4.3)	471.3474 439.2809	3-O-Acetyl-16α-hydroxytrametenolic acid isomer	
31	5.26	C_31_H_46_O_5_	497.3270[M-H]^−^(0.6) 543.3356[M-H]^−^(6.3) 995.6573[2M-H]^−^(−3.9)	481.2930[M-H-CH_4_]^−^ 419.2980[M-H-H_2_O-CO_2_-CH_4_]^−^ 483.3155	25-Hydroxyporicoic acid C/25-Hydroxypolyporenic acid C/ 6-Hydroxypolyporenic acid C/Poricoic acid BM/ 3-Oxo-6,16α-dihydroxy-lanosta-7,9(11),24(31)-trien-21-oic acid	Li, 2013; Wang, 2016; Zhu et al., 2018b
32	5.43	C_31_H_48_O_5_	499.3418[M-H]^−^(−1.0)	481.3334[M-H-H_2_O]^−^	Poricoic acid H	Zou et al., 2019
33	5.62	C_31_H_46_O_5_	497.3270[M-H]^−^(0.6) 543.3356[M-H]^−^(6.3) 995.6573[2M-H]^−^(−3.9)	483.3155 469.3313 467.3162	25-Hydroxyporicoic acid C/25-Hydroxypolyporenic acid C/ 6-Hydroxypolyporenic acid C/Poricoic acid BM/ 3-Oxo-6,16α-dihydroxy-lanosta-7,9(11),24(31)-trien-21-oic acid	Li, 2013; Wang, 2016; Zhu et al., 2018b
34	5.82	C_33_H_50_O_5_	525.3594[M-H]^−^(2.7) 571.3607[M-H+HCOOH]^−^(−4.9)	465.3363[M-CO_2_-CH_4_]^−^ 471.3463	16α-Acetytoxyeburiconic	Zou et al., 2019
35	5.97	C_30_H_44_O_4_	467.3162[M-H]^−^(0.2) 513.3177[M-H+HCOOH]^−^(−7.6)	407.2950[M-H-CO_2_-CH_4_]^−^ 393.2802[M-H-C_2_H_5_COOH]^−^	16-Deoxyporicoic acid B/ 25-Methoxyporicoic acid A	Wang, 2016; Zou et al., 2019
36	6.11	C_32_H_48_O_6_	527.3361[M-H]^−^(−2.3) 573.3436 [M-H+HCOOH]^−^(1.6)	481.3065 419.1387	Poricoic acid DM	Zhu et al., 2018b
37	6.28	C_31_H_46_O_5_	497.3316[M-H]^−^(9.9)	467.3118[H-H-HCOOH]^−^ 483.3115	25-Hydroxyporicoic acid C/25-Hydroxypolyporenic acid C/ 6-Hydroxypolyporenic acid C/Poricoic acid BM/ 3-Oxo-6,16α-dihydroxy-lanosta-7,9(11),24(31)-trien-21-oic acid	Li, 2013; Wang, 2016; Zhu et al., 2018b
38	6.41	C_31_H_50_O_4_	485.3603[M-H]^−^(−5.8) 531.3666[M-H+HCOOH]^−^(−3.8)	469.3623 427.2726	3β-Hydroxy-25-methoxy-24-methylene-27-Norlanost-8-en-21-oic acid/3-Oxo-6,16α-dihydroxy-lanosta-8,24-dien-21-oic acid	Zhu et al., 2018b Wang, 2014
39^*^	6.57	C_30_H_46_O_4_	469.3313[M-H]^−^(−1.1) 515.3367[M-H+HCOOH]^−^(−1.2) 939.6746[2M-H]^−^(3.4)	423.3264[M-H-HCOOH]^−^ 409.3091[M-H-CO_2_-CH_4_]^−^ 407.2993[M-H-HCOOH-CH_4_]^−^ 375.2686[M-H-H_2_O-CO_2_-2CH_4_]^−^ 311.1979[M-H-C_8_H_14_O_2_-CH_4_]^−^	16α-Hydroxydehydrotrametenolic acid	Wang, 2016
40	6.81	C_31_H_48_O_4_	483.3470[M-H]^−^(−0.8) 529.3495[M-H+HCOOH]^−^(−6.4)	439.2552 437.2928	3-epi-Dehydrotumulosic acid	Wang, 2016
41^*^	6.96	C_30_H_48_O_4_	471.3463[M-H]^−^(−1.1) 517.3549[M-H+HCOOH]^−^(3.9) 943.7065[2M-H]^−^ (4.0)	453.3361[M-H-H_2_O]^−^ 427.3487[M-H-CO_2_]^−^ 409.3091[M-H-HCOOH-CH_4_]^−^	16α-Hydroxytrametenolic acid	Zhu et al., 2018b
42	7.16	C_33_H_50_O_6_	541.3530[M-H]^−^(0.2) 587.3599[M-H+HCOOH]^−^(2.6)	481.3155[M-H-CH_3_COOH]^−^ 465.3010[M-H-CH_3_COOH-CH_4_]^−^ 461.3056[M-H-HCOOH-CH_4_-H_2_O]^−^	6α-Hydroxydehydropachymic acid	Zhu et al., 2018b
43	7.23	C_31_H_46_O_4_	481.3334[M-H]^−^(3.3) 527.3361[M-H+HCOOH]^−^(−2.3)	407.2930[M-H-C_2_H_5_COOH]^−^	Poricoic acid C	Zhu et al., 2018b
44	7.40	C_33_H_48_O_6_	539.3406[M-H]^−^(6.1)	521.2177	16α-Acetoxy-26,27-dimethoxyl-lanosta-8,24(31)-dien-21-oic acid	Zhu et al., 2018b
45^*^	7.56	C_30_H_44_O_5_	483.3110[M-H]^−^(0.0) 529.3212[M-H+HCOOH]^−^(8.9) 967.6332[2M-H]^−^(4.0)	409.2718[M-H-C_2_H_5_COOH]^−^	Poricoic acid B	Zhu et al., 2018b
46	7.77	C_31_H_46_O_5_	497.3315[M-H]^−^(9.7)	483.3110 467.3029	25-Hydroxyporicoic acid C/25-Hydroxypolyporenic acid C/ 6-Hydroxypolyporenic acid C/Poricoic acid BM/ 3-Oxo-6,16α-dihydroxy-lanosta-7,9(11),24(31)-trien-21-oic acid	Li, 2013; Wang, 2016; Zhu et al., 2018b
47^*^	7.89	C_31_H_48_O_4_	483.3470[M-H]^−^(−0.8) 529.3542[M-H+HCOOH]^−^(2.5) 967.7032[2M-H]^−^(0.5)	439.3538[M-H-CO_2_]^−^ 437.3400[M-H-HCOOH]^−^ 421.3119[M-H-HCOOH-CH_4_]^−^ 389.2847[M-H-H_2_O-CO_2_-2CH_4_]^−^ 311.2015[M-H-C_9_H_16_O_2_-CH_4_]^−^	Dehydrotumulosic acid	Zhu et al., 2018b
48	8.20	C_32_H_50_O_6_	529.3542[M-H]^−^(2.5)	483.3470[M-H-HCOOH]^−^ 469.3313[M-H-CO_2_-CH_4_]^−^	3β-Acetyloxy-16α,26-dihydroxy-Lanosta-8,24-dien-21-oic acid	Zhu et al., 2018b
49	8.47	C_31_H_50_O_4_	485.3648[M-H]^−^(3.5) 531.3666[M-H+HCOOH]^−^(−3.8) 971.7372[2M-H]^−^(3.3)	469.3313[M-H-CH_4_]^−^ 423.3264[M-H-HCOOH-CH_4_]^−^ 391.3001[M-H- H_2_O-CO_2_-2CH_4_]^−^	Tumulosic acid	Wang, 2016; Zhu et al., 2018b
50	8.84	C_31_H_46_O_5_	497.3270[M-H]^−^(0.6)	479.3151[M-H-H_2_O]^−^	25-Hydroxyporicoic acid C/25-Hydroxypolyporenic acid C/ 6-Hydroxypolyporenic acid C/Poricoic acid BM/ 3-Oxo-6,16a-dihydroxy-lanosta- 7,9(11),24(31)-trien-21-oic acid	Li, 2013 Wang, 2016 Zhu et al., 2018b
51^*^	9.00	C_31_H_46_O_5_	497.3270[M-H]^−^(0.6) 543.3308[M-H+HCOOH]^−^(−2.6) 995.6658[2M-H]^−^(4.6)	479.3151[M-H-H_2_O]^−^ 423.2885[M-H-C_2_H_5_COOH]^−^ 379.2993[M-H-C_2_H_5_COOH-CO_2_]^−^ 363.2688[M-H-C_2_H_5_COOH-CO_2_-CH_4_]^−^	Poricoic acid A	Zhu et al., 2018b
52	9.20	C_30_H_46_O_5_	485.3242[M-H]^−^(−5.2) 531.3336[M-H+HCOOH]^−^(2.6)	467.3162[M-H_2_O]^−^ 441.3336[M-H-CO_2_]^−^ 423.2927[M-H-HCOOH-CH_4_]^−^	Poriacosone A/Poriacosone B	Zheng and Yang, 2008; Zou et al., 2019
53	9.43	C_30_H_46_O_4_	469.3313[M-H]^−^(−1.1) 515.3367[M-H+HCOOH]^−^(−1.2)	451.3209[M-H-H_2_O]^−^ 423.3011 405.2698 375.2607	15α-Hydroxy-3-oxolanosta-8,24-dien-21-oic acid	Zhu et al., 2018b; Zou et al., 2019
54	9.77	C_33_H_52_O_6_	543.3690[M-H]^−^(0.7) 589.3753[M-H+HCOOH]^−^(2.2)	483.3425[M-H-CO_2_-CH_4_]^−^ 481.3289[M-H-HCOOH-CH_4_]^−^ 467.3516 437.3229	25-Hydroxypachymic acid	Zhu et al., 2018b
55	9.99	C_31_H_48_O_4_	483.3515[M-H]^−^(8.5) 529.3542[M-H+HCOOH]^−^(2.5)	439.3452, 437.3015	Dehydrotumulosic acid isomer	
56^*^	10.23	C_31_H_46_O_4_	481.3334[M-H]^−^(3.3) 527.3361[M-H+HCOOH]^−^(−2.3)	437.3400[M-H-CO_2_]^−^ 435.3265[M-H-HCOOH]^−^ 421.3119[M-H-CO_2_-CH_4_]^−^ 405.2780[M-H-CO_2_-2CH_4_]^−^ 403.2988[M-H-H_2_O-CO_2_-CH_4_]^−^	Polyporenic acid C	Zhu et al., 2018b
57	10.48	C_31_H_46_O_4_	481.3289[M-H]^−^(−6.0)	421.3077[M-H-CO_2_-CH_4_]^−^ 469.3313 325.1749	Polyporenic acid C isomer	
58	10.62	C_33_H_50_O_6_	541.3530[M-H]^−^(0.2)	495.3474[M-H-HCOOH]^−^ 481.3334[M-H-CH_3_COOH]^−^ 293.2087	29-Hydroxydehydropachymic acid	Zou et al., 2019
59	10.75	C_31_H_48_O_4_	483.3470[M-H]^−^(0.8) 529.3495[M-H+HCOOH]^−^(6.4)	439.3581[M-H-CO_2_]^−^ 437.3400[M-H-HCOOH]^−^ 423.3222[M-H-CO_2_-CH_4_]^−^ 421.3077[M-H-HCOOH-CH_4_]^−^	15α-Hydroxyeburiconic acid	Zou et al., 2019
60	10.94	C_31_H_48_O_5_	499.3418[M-H]^−^(−1.0) 545.3458[M-H+HCOOH]^−^(−3.7)	481.3334[M-H-H_2_O]^−^ 485.3242	16α,29-Dihydroxyeburiconic acid	Zou et al., 2019
61	11.21	C_31_H_48_O_4_	483.3470[M-H]^−^(0.8) 529.3542[M-H+HCOOH]^−^(2.5)	439.3581[M-H-CO_2_]^−^ 437.3400[M-H-HCOOH]^−^ 423.3264[M-H-CO_2_-CH_4_]^−^ 407.2952[M-H-CO_2_-2CH_4_]^−^ 389.2806[M-H-H_2_O-CO_2_-2CH_4_]^−^	16α-Hydroxyeburiconic acid	Zou et al., 2019
62	11.50	C_33_H_52_O_6_	543.3642[M-H]^−^(−8.1)	483.3425[M-H-CO_2_-CH_4_]^−^	25-Hydroxypachymic acid isomer	Zhu et al., 2018b
63	11.67	C_33_H_52_O_6_	543.3642[M-H]^−^(−8.1)	483.3470[M-H-CO_2_-CH_4_]^−^	25-Hydroxypachymic acid isomer	Zhu et al., 2018b
64	11.89	C_31_H_48_O_4_	483.3425[M-H]^−^(−10.1) 529.3495[M-H+HCOOH]^−^(6.4)	421.2951 407.2498	Dehydrotumulosic acid isomer	Zou et al., 2019
65	12.2	C_31_H_50_O_4_	485.3648[M-H]^−^(3.5)	423.3264[M-H-HCOOH-CH_4_]^−^ 295.2237	Hispindic acid B	Zou et al., 2019
66	12.39	C_32_H_48_O_5_	511.3444[M-H]^−^(4.1) 557.3446[M-H+HCOOH]^−^(−5.7)	451.3209[M-H-CH_3_COOH]^−^ 467.3162 465.3363	3β-Acetoxy-16α,26-dihydroxy-lanosta-8,24-dien-21-oic acid/Poricoic acid AM	Wang, 2016; Zhu et al., 2018b; Zou et al., 2019
67	12.59	C_32_H_46_O_5_	509.3286[M-H]^−^(3.7) 555.3269[M-H+HCOOH]^−^(7.7)	449.3056[M-H-CO_2_-CH_4_]^−^	Unknown	
68	12.88	C_20_H_28_O_2_	299.1999[M-H]^−^(−4.0)		Unknown	
69	13.19	C_26_H_56_O_11_	543.3738[M-H]^−^(−1.1)		Unknown	
70	13.41	C_32_H_50_O_5_	513.3548[M-H]^−^(−6.2) 559.3611[M-H+HCOOH]^−^(−4.3)	469.3224 325.1823	3-O-Acetyl-16α-hydroxytrametenolic acid isomer	
71	13.48	C_35_H_54_O_6_	569.3862[M-H]^−^(3.5)		16-O-Acetylpachymic acid/ 3β,15α-Bis(acetyloxy)-24-dien-21-oic acid	Wang, 2016; Zou et al., 2019
72	13.58	C_32_H_48_O_5_	511.3397[M-H]^−^(−5.1) 557.3446[M-H+HCOOH]^−^(−5.7)	451.3078 353.2073 293.1737	3β-Acetoxy-16α,26-dihydroxy-lanosta-8,24-dien-21-oic acid /Poricoic acid AM	Wang, 2016; Zhu et al., 2018b; Zou et al., 2019
73	13.81	C_32_H_48_O_5_	511.3397[M-H]^−^(−5.1) 557.3446[M-H+HCOOH]^−^(−5.7)	451.3078 353.2073 293.1737	3β-Acetoxy-16α,26-dihydroxy-lanosta-8,24-dien-21-oic acid/ Poricoic acid AM	Wang, 2016; Zhu et al., 2018b; Zou et al., 2019
74	14.03	C_33_H_52_O_5_	527.3737[M-H]^−^(0.2)	511.3351 339.1989	Pachymic acid isomer	
75^*^	14.24	C_32_H_48_O_5_	511.3397[M-H]^−^(−5.1) 557.3495[M-H+HCOOH]^−^(3.1)	467.3516[M-H-CO_2_]^−^ 465.3363[M-H-HCOOH]^−^ 451.3209[M-H-CH_3_COOH]^−^ 355.2295[M-H-C_9_H_16_O_2_]^−^	3-O-Acetyl-16α-hydroxydehydrotrametenolic acid	Zhu et al., 2018b; Zou et al., 2019
76	14.43	C_33_H_50_O_5_	525.3594[M-H]^−^(2.7) 571.3607[M-H+HCOOH]^−^(−4.9)	463.3166[M-H-HCOOH-CH_4_]^−^ 449.3058[M-H-CH_3_COOH-CH_4_]^−^	3-epi-Dehydropachymic acid	Zou et al., 2019 Zhu et al., 2018b
77	14.60	C_33_H_48_O_5_	523.3444[M-H]^−^(4.0) 569.3471[M-H+HCOOH]^−^(−1.2)	463.3210[M-H-CH_3_COOH]^−^	16α-Acetoxypolyporenic acid C	Wang, 2016
78	14.99	C_32_H_50_O_5_	513.3594[M-H]^−^(2.7)	497.3179	3-O-Acetyl-16α-hydroxytrametenolic acid isomer	
79	15.12	C_34_H_42_O_2_	481.3199[M-H]^−^(2.5)		Unknown	
80^*^	15.41	C_32_H_50_O_5_	513.3594[M-H]^−^(2.7) 559.3660[M-H+HCOOH]^−^(4.5)	453.3361[M-H-CH_3_COOH]^−^ 451.3209[M-H-HCOOH-CH_4_]^−^	3-O-Acetyl-16α-hydroxytrametenolic acid	Wang, 2014; Zhu et al., 2018b
81	15.87	C_32_H_50_O_5_	513.3548[M-H]^−^(−6.2) 559.3660[M-H-HCOOH]^−^(4.5)		3-O-Acetyl-16α-hydroxytrametenolic acid isomer	
82	16.11	C_32_H_50_O_5_	513.3548[M-H]^−^(−6.2) 559.3660[M-H-HCOOH]^−^(4.5)		3-O-Acetyl-16α-hydroxytrametenolic acid isomer	
83^*^	16.33	C_33_H_50_O_5_	525.3594[M-H]^−^(2.7) 571.3657[M-H-HCOOH]^−^(3.9)	481.3649[M-H-CO_2_]^−^ 471.3509[M-H-HCOOH]^−^ 465.3363[M-H-CH_3_COOH]^−^ 463.3166[M-HCOOH-CH_4_]^−^	Dehydropachymic acid	Zhu et al., 2018b
84	16.82	C_31_H_48_O_4_	483.3425[M-H]^−^(−10.1) 529.3495[M-H+HCOOH]^−^(6.4)	465.3010 309.0355	Dehydrotumulosic acid isomer	Zou et al., 2019
85	17.09	C_33_H_52_O_5_	527.3737[M-H]^−^(0.2) 573.3779[M-H+HCOOH]^−^(−2.1)	467.3604[M-H-CH_3_COOH]^−^ 449.3448	3-eip-Pachymic acid	Wang, 2016
86^*^	17.56	C_33_H_52_O_5_	527.3737[M-H]^−^(0.2) 573.3779[M-H+HCOOH]^−^(−2.1) 1055.7560[2M-H]^−^(0.9)	481.3649[M-H-HCOOH]^−^ 467.3516[M-CH_3_COOH]^−^ 465.3363[M-H-HCOOH-CH_4_]^−^ 339.1951[M-H-C_9_H_16_O_2_-2CH_4_]^−^	Pachymic acid	Zhu et al., 2018b
87	18.12	C_30_H_44_O_4_	467.3162[M-H]^−^(0.2) 935.6389[2M-H]^−^−1.3 513.3177[M-H+HCOOH]^−^(−7.6)	393.2802[M-H-C_2_H_5_COOH]^−^ 449.3362[M-]	16α-Hydroxydehydrotrametenonic	Zou et al., 2019
88	18.37	C_29_H_46_O_5_	473.3311[M-H]^−^(9.3)		Unknown	
89	18.90	C_34_H_50_O_6_	553.3514[M-H] ^−^(−2.7)	425.2909 367.3501	3,15-O-Diacetyl-dehydrotrametenolic Acid	Chen et al., 2019
90	19.10	C_30_H_44_O_3_	451.3209[M-H] ^−^(−0.7)	433.1260	16α-Hydroxy-lanosta-7,9(11),24-trien-21-oic acid	Wang, 2016
91	19.7	C_35_H_50_O	485.3746[M-H] ^−^(−7.6)		Unknown	Wang, 2014
92	19.88	C_25_H_42_O_9_	485.2791[M-H]^−^(8.2)	453.3318	Unknown	Zhu et al., 2018b
93	19.96	C_30_H_46_O_3_	453.3361[M-H] ^−^(−1.8) 499.3418[M-H+HCOOH] ^−^(−1.0)	435.3351 409.2429	3β-Hydroxylanosta-7,9(11),24-trien-21-oic acid	Zhu et al., 2018b
94	20.16	C_32_H_52_O_4_	499.3783[M-H] ^−^(−0.8) 545.3888[M-H+HCOOH]^−^(8.4)		Unknown	
95	20.45	C_23_H_32_O_2_	339.2328[M-H]^−^(1.2)		Unknown	
96	20.72	C_31_H_46_O_4_	481.3334[M-H]^−^(3.3) 527.3361[M-H+HCOOH] ^−^(−2.3)	437.3400[M-CO_2_]^−^ 421.3119[M-CO_2_-CH_4_]^−^	Polyporenic acid C isomer	
97	20.94	C_38_H_52_O_5_	587.3748[M-H]^−^(2.0)		Unknown	
98	21.08	C_35_H_52_O_6_	567.3711[M-H]^−^(4.4)	351.2523	Unknown	
99^*^	21.72	C_30_H_46_O_3_	453.3361[M-H] ^−^(−1.8) 499.3418[M-H-HCOOH] ^−^(−1.0) 907.6812[2M-H] ^−^(−0.4)	435.3265[M-H_2_O]^−^	Dehydrotrametenolic acid	Zhu et al., 2018b
100	22.29	C_48_H_42_O_4_	681.3003[M-H] ^−^(−0.3)		Unknown	Zhu et al., 2018b
101	22.46	C_35_H_54_O_6_	569.3862[M-H]^−^(3.5) 615.3923[M-H+HCOOH]^−^(4.2)		16-O-Acetylpachymic acid/ 3β,15α-Bis (acetyloxy)-24-dien-21-oic acid	Zou et al., 2019 Wang, 2016;
102	22.69	C_31_H_48_O_3_	467.3516[M-H] ^−^(−1.9) 513.3594,[M-H+HCOOH]^−^(2.7)	371.2554, 339.2668	Dehydroeburicoic acid isomer	
103	22.98	C_30_H_48_O_3_	455.3515[M-H] ^−^(−2.2) 501.3605[M-H+HCOOH]^−^(5.0)	437.3315	Trametenolic acid/Oleanolic acid	Wang, 2016
104	23.37	C_30_H_48_O_3_	455.3515[M-H] ^−^(−2.2) 501.3605[M-H+HCOOH]^−^(5.0)	437.3315	Trametenolic acid/Oleanolic acid	Zhu et al., 2018b
105	24.03	C_21_H_34_O_3_	333.2399[M-H] ^−^(−9.3)		Unknown	
106^*^	24.43	C_31_H_48_O_3_	467.3516[M-H] ^−^(−1.9) 513.3594[M-H-HCOOH]^−^(2.7) 935.7140[2M-H]^−^(1.2)	371.2554, 337.2531, 339.2668 279.2256	Dehydroeburicoic acid	Zhu et al., 2018b
107	24.72	C_30_H_44_O_3_	451.3209[M-H] ^−^(−0.7) 497.3270[M-H+HCOOH]^−^(0.6)	409.3091 325.1786	Porilactone A/Porilactone B	Chen et al., 2019
108	25.09	C_22_H_40_O_4_	367.2874[M-H]^−^(7.1)		Unknown	
109^*^	25.78	C_31_H_50_O_3_	469.3668[M-H] ^−^(−3.0) 515.3738[M-H]^−^(0.4)	453.3492 373.2771 339.2668 279.2227	Eburicoic acid	Zhu et al., 2018b; Wang, 2016
110	26.20	C_40_H_60_O_5_	619.4365[M-H]^−^(0.5) 665.4432[M-H+HCOOH]^−^(2.3)	469.3668 465.3363	Unknown	
111	26.60	C_18_H_34_O_2_	281.2469[M-H] ^−^(−4.3)		Unknown	
112	27.22	C_31_H_46_O_3_	465.3363[M-H] ^−^(−1.3) 511.3397[M-H+HCOOH] ^−^(−5.1)		Unknown	Chen et al., 2019
113	27.62	C_31_H_46_O_3_	465.3363[M-H] ^−^(−1.3) 511.3397[M-H+HCOOH] ^−^(−5.1)		Unknown	Wang, 2016
114	28.09	C_31_H_48_O_3_	467.3516[M-H] ^−^(−1.9)		Dehydroeburicoic acid isomer	
115	28.31	C_27_H_28_O_8_	479.1717[M-H]^−^(2.7)		Unknown	
116	28.87	C_24_H_44_O_7_	443.3006[M-H] ^−^(−0.7)		Unknown	
117	29.23	C_33_H_62_O_10_	617.4265[M-H] ^−^(−2.9)		Unknown	
118	29.43	C_30_H_50_O_4_	473.3612[M-H] ^−^(−4.0)		Unknown	
119	29.56	C_32_H_48_O_4_	495.3483[M-H]^−^(1.8) 541.3482[M-H+HCOOH] ^−^(−8.7)	473.3612	Poricoic acid CM	Zhu et al., 2018b

* *Identified by comparing with standard compounds*.

**Figure 3:**
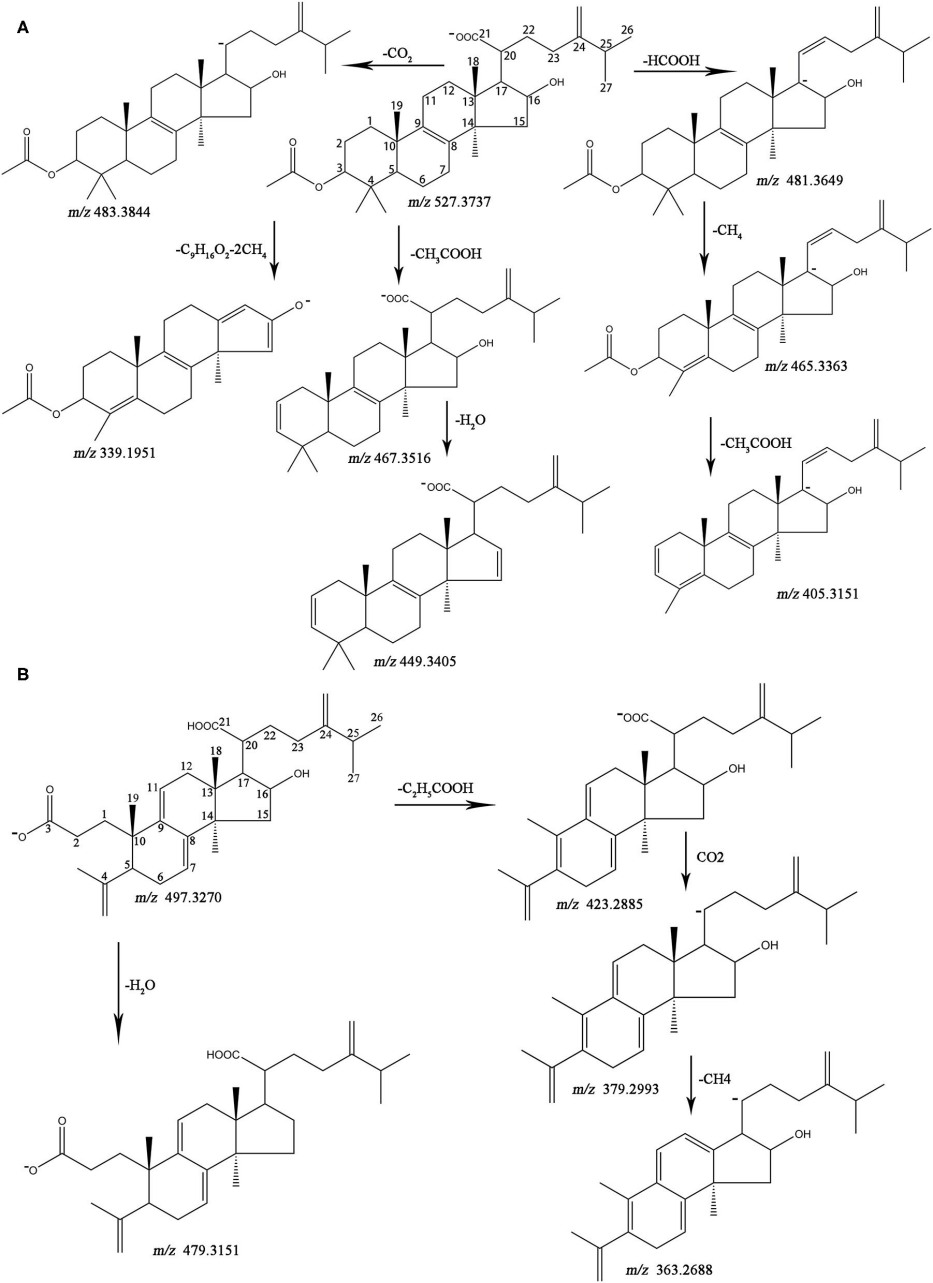
The fragmentation pathways for pachymic acid **(A)** and porioic acid A **(B)** in ESI^−^.

The MS data and structures of the 13 reference compounds are presented in Supplementary Figure 3. Peaks 39, 41, 45, 47, 51, 56, 75, 80, 83, 86, 99, 106, and 109 were identified as 16α-hydroxydehydrotrametenolic acid, 16α-hydroxytrametenolic acid, poricoic acid A, dehydrotumulosic, poricoic acid B, polyporenic acid C, 3-O-acetyl-16α-hydroxydehydrotrametenolic acid, 3-O-acetyl-16α-hydroxytrametenolic acid, dehydropachymic acid, pachymic acid, dehydrotrametenolic acid, and dehydroeburicoic acid, respectively, by comparing the MS data with the reference standards. Triterpenoid acids are the main specialized metabolites of Fushen, which are biosynthesized *via* the mevalonic acid pathway, thus share numerous common skeletons and modifications (Zou et al., 2019). Pachymic acid and poricoic acid A are shown as examples to illustrate the cracking of fragment ions in the triterpenoid acids in the negative ion mode (Figure 3).

Pachymic acid showed [M-H]^−^ at *m*/*z* 527.3737 (0.2 ppm), [M-H+HCOOH]^−^ at *m*/*z* 573.3779 (−2.1 ppm), [2M-H]^−^ at *m*/*z* 1055.7560 (0.9 ppm) in the negative ion mode, which supports the molecular formula C_33_H_52_O_5_. The fragment ions at *m*/*z* 483.3844 (1.2 ppm) and *m*/*z* 481.3649 (−6.9 ppm) were generated from the neutral loss of CO_2_ (44 Da) and HCOOH (46 Da) at the C-20 position. A separate fragment with *m*/*z* 467.3516 (−1.9 ppm) lost 60 Da, which was consistent with the loss of an acetoxyl group from C-3. The fragment ion [M-H-HCOOH-CH_4_]^−^ at *m/z* 465.3363 (−1.3 ppm), corresponded to a carboxyl group loss at the C-20 position and a methyl group loss at the C-4 position. The fragment ion at *m*/*z* 449.3405 (−3.3 ppm) indicated a neutral loss of CH_3_COOH (60 Da) at the C-3 position and H_2_O (18 Da) at the C-16 position. The ion [M-H-HCOOH-CH_4_-CH_3_COOH]^−^ at *m*/*z* 405.3151 (−1.5 ppm) was observed. The ion [M-H-C_9_H_16_O_2_-2CH_4_]^−^ resulted from the neutral loss of the C_9_H_16_O_2_ (156 Da) side chain at C-17 and two CH_4_ (18 Da). Poricoic acid A exhibited the following quasi-molecular ions [M-H]^−^, [M-H+HCOOH]^−^, and [2M-H]^−^ at *m*/*z* 497.3270 (0.6 ppm), *m*/*z* 543.3308 (−2.6 ppm), and *m*/*z* 995.6658 (4.6 ppm), respectively, supported the molecular formula C_31_H_46_O_5_. The product ion [M-H-H_2_O]^−^ at *m*/*z* 479.3151 (−2.1 ppm) indicated the loss of the hydroxyl group at C-16. The characteristic ion [M-H-C_2_H_5_COOH-CO_2_-CH_4_]^−^ at *m*/*z* 363.2688 (4.4 ppm) showed the neutral loss of C_2_H_5_COOH (62 Da), CO_2_ (44 Da), and CH_4_ (16 Da) at C-10, C-20, and C-13 positions, respectively. The fragment ions [M-H-C_2_H_5_COOH]^−^ and [M-H-C_2_H_5_COOH-CO_2_]^−^ at *m*/*z* 423.2885 (−3.3 ppm) and *m*/*z* 379.2993 (−2.1 ppm) were also observed. The other reference compounds were also analyzed, and their characteristic ions are listed in Table 1.

After the analysis of the characteristic fragmentation pathways and diagnostic ions with reference standards, other compounds were tentatively identified. Compounds 19, 21, 26, 29, 32, and 60 showed the same formula as C_31_H_48_O_5_. According to the elution order and the corresponding ion fragments, compounds 19, 21, 26, 29, 32, and 60 were tentatively identified as poricoic acid GM, 29-hydroxydehydrotumulosic acid, 16α,25-dihydroxyeburiconic acid or 25-hydroxy-3-epi-dehydrotumulosic acid, 15α-hydroxydehydrotumulosic acid, poricoic acid H, and 16α,29-dihydroxyeburiconic acid, respectively (Zou et al., 2019). Compounds 27, 31, 33, 37, 46, 50, and 51 showed the same formula as C_31_H_46_O_5_. Compound 51 was unequivocally identified as poricoic acid A in comparison with the reference compound. Compounds 27, 31, 33, 37, 46, and 50 were tentatively identified as 25-hydroxyporicoic acid C or 25-hydroxypolyporenic acid C or 6-hydroxypolyporenic acid C or poricoic acid BM or 3-oxo-6,16α-dihydroxy-lanosta-7,9(11),24(31)-trien-21-oic acid, owing to the deficiencies of the corresponding reference standards. For compound 80, fragment ions such as [M-H-CH_3_COOH]^−^ at *m*/*z* 453.3361 (−1.8 ppm) and [M-H-HCOOH-CH_4_]^−^ at *m/z*451.3209 (−0.7 ppm) were observed, which unequivocally identified as 3-o-acetyl-16α-hydroxytrametenolic acid in comparison with the reference compound. Compounds 70, 78, 81, and 82 showed the same formula C_32_H_50_O_5_ as compound 80 and were tentatively identified as 3-o-acetyl-16α-hydroxytrametenolic acid isomer. Considering the similarities in retention behavior and the substitutions with those of reference compound 86, compound 85 was tentatively identified as 3-epi-pachymic acid. Compound 49 was tentatively identified as tumulosic acid as it was relatively abundant in the Fushen extract, and the fragmentation patterns were in line with those presented in the literature (Zou et al., 2019). In addition, 35 compounds were unknown (compounds 1, 2, 4–14, 67–69, 79, 88, 91, 92, 94, 95, 97, 98, 100, 105, 108, 110–113, 115–118). The other compounds are tentatively identified in Table 1. Some trace triterpene acids were only tentatively identified according to the quasi-molecular ions and the retention capabilities, owing to the deficiencies of the corresponding reference standards, literature, and the lack of available high-quality MS/MS spectra.

### Quantitative Analysis Method Validation

The validation results of the UPLC quantitative method are listed in Supplementary Table 4. All calibration curves showed good linearity over relatively wide concentration ranges (*r* ≥ 0.999). The LODs and LOQs were 0.0008–0.0430 mg/ml and 0.0034–0.1200 mg/ml, respectively. The RSDs of the intraday and inter-day precision were shown to be 0.27–1.58 and 0.97–2.94%, respectively. The RSDs of the repeatability and stability were <2.50 and 2.51%, respectively. The average recoveries of the analytes ranged from 96.38 to 103.65% with RSDs lower than 4.00%, indicating the reliability and feasibility of the approach. These results showed that the validated method was sensitive, repeatable, stable, and accurate for the simultaneous determination of the 13 reference compounds.

### Accumulation of Specialized Metabolites in Fushen at Different Growth Periods

There were obvious differences in the number of specialized metabolites in Fushen during the nine growth periods. In June, a total of 20 compounds were identified, but they were all unknown. In August, 84 compounds were detected and identified or putatively characterized. In September, October, November, and December, 94, 98, 99, and 100 compounds were identified, while a total of 102 compounds were characterized in January, March, and April.

#### Multivariate Statistical Analysis

**Figure 4:**
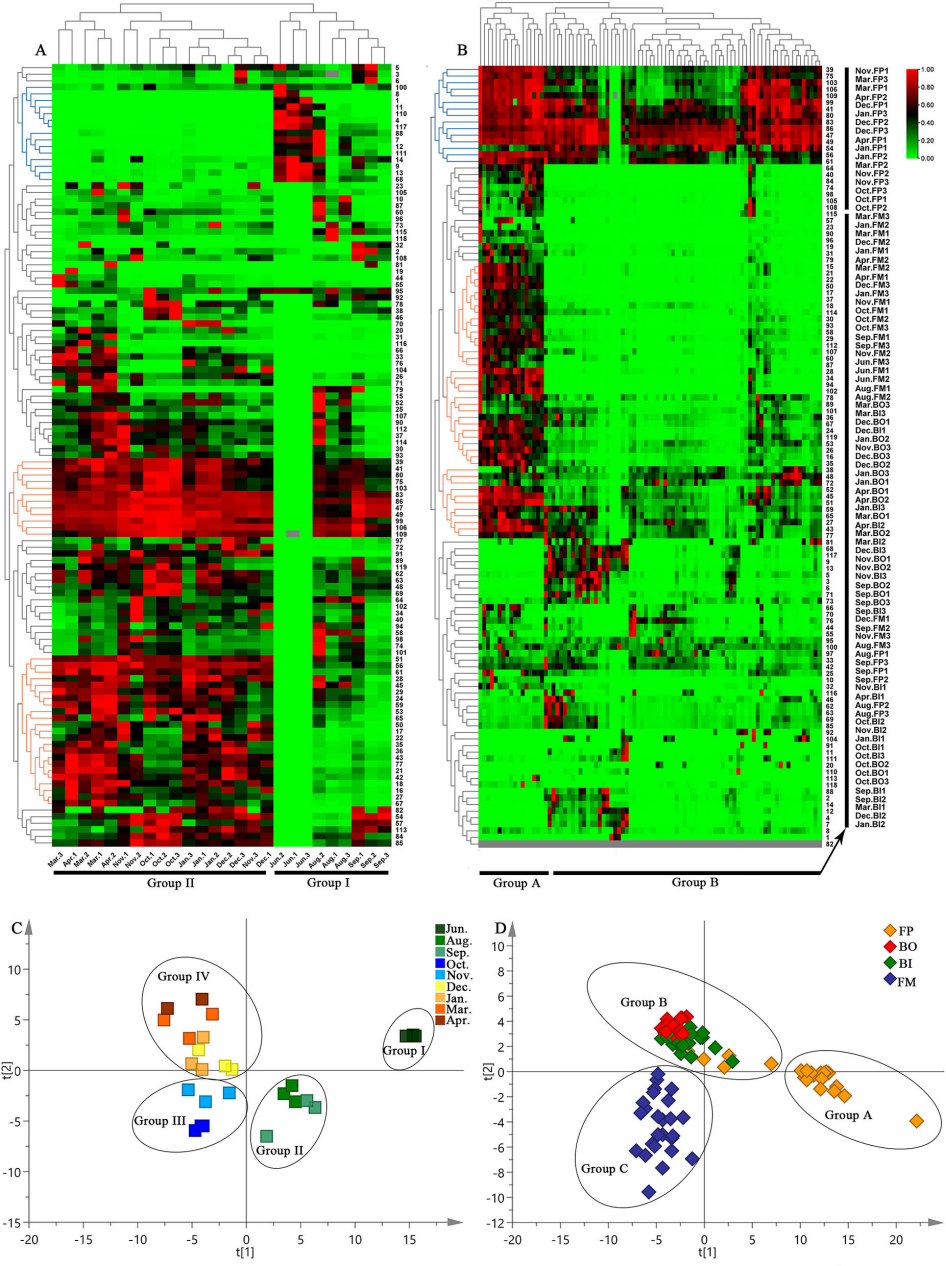
Hierarchical clustering analysis heat maps for the nine growth periods **(A)** and four parts **(B)** of the Fushen, PLS-DA score plots of nine growth periods **(C)** and four parts **(D)** of the Fushen based on UPLC-Q/TOF-MS in ESI^−^.

To further characterize the dissimilarity of the specialized metabolites in the nine growth periods of Fushen, multivariate statistical analysis, a hierarchical clustering analysis heat map, PCA, and PLS-DA were used to analyze the 119 specialized metabolites in the negative ion mode. The hierarchical clustering analysis heat map of the nine growth periods of Fushen is shown in Figure 4A. The colors represent the level of accumulation for each metabolite, from low (green) to high (red). The samples were clearly classified into two clusters, as the samples from June, August, and September were in Group I, and the samples from October, November, December, January, March, and April were in Group II. Compounds 39, 41, 80, 75, 103, 83, 86, 47, 49, 99, 106, 109, 51, 56, 61, 28, 45, 29, 24, 59, 53, 65, 50, 17, 22, 35, 36, 43, 77, 21, 42, 18, 16, 27 and 67 had higher accumulation levels in Group II, while 15 unknown compounds (compounds 100, 8, 1, 11, 110, 4, 117, 88, 7, 12, 111, 14, 9, 13, and 68) had higher accumulation levels in Group I, especially in June.

After Pareto scaling with mean-centering, the PCA score plots showed that the nine QCs were tightly clustered in the center of the plots (Supplementary Figure 4A). The results verified the reliability of the analysis using UPLC-Q/TOF-MS. To identify markers for the nine growth periods, a supervised PLS-DA model was used (Figure 4C). The nine growth periods were divided into four groups: the samples from June 1 to 3 were classified as Group I; August 1–3 and September 1–3 were classified as Group II; October 1–3 and November 1–3 were classified as Group III; and the samples from the other periods as Group IV. Compared with the hierarchical clustering analysis heat map, PLS-DA performed a detailed classification. The resulting R^2^Y- and Q^2^-values in this study were >0.7, indicating excellent fitness and predictability of the PLS-DA mode (Kang et al., 2017). Subsequently, the VIP approach was used to evaluate the importance of the variables in the projection of the PLS-DA model. In general, features with a VIP >1 were considered to carry the most relevant information for class discrimination (Cho et al., 2008). Thirty-two specific markers were found to possess high discrimination potentials (Supplementary Table 5). Compounds 45, 47, 75, 80, and 109 were unambiguously identified by matching their retention times and accurate masses with those of the authentic standards. Other specific markers (compounds 15, 21, 22, 25, 33, 35, 36, 38, 43, 48, 52, 54, 57, 58, 60, 64, 65, 66, 67, 74, 76, 77, 90, 92, 101, 104, and 113) were also tentatively identified. In addition, the PLS-DA model was validated using a permutation test (Supplementary Figure 4B). The R^2^Y intercept was below 0.3, and the Q^2^Y intercept was below 0.0 in the PLS-DA model, which was another strong indication of the validity of the models (Chatterjee et al., 2019).

#### Quantitative Analysis of 13 Triterpenoid Acids

**Figure 5:**
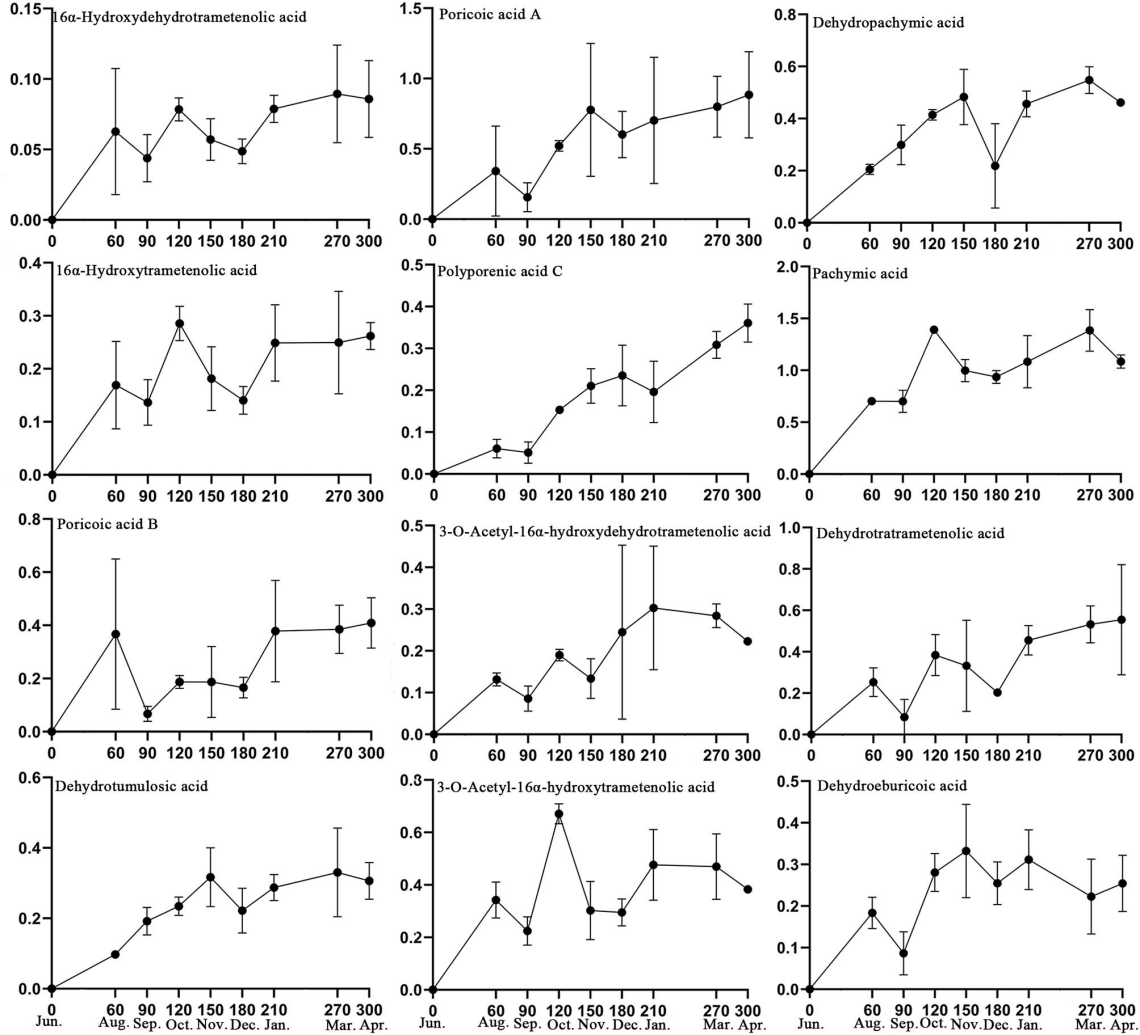
Content levels (mg/g) for the 13 triterpenoid acids in the nine growth periods of Fushen.

Based on the 32 specific makers identified in this study and the active compounds of Fushen that have been reported in the literature (Sun et al., 2019; Deng et al., 2020), 13 triterpenoid acids were selected for simultaneous determination in 26 Fushen samples with nine growth periods by UPLC (Table 2, Supplementary Figure 5). The pachymic acid content (0.701 ± 0.107 to 1.391 ± 0.035 mg/g) was the highest in all the samples, followed by poricoic acid A (0.156 ± 0.103 to 0.885 ± 0.306 mg/g), dehydrotrametenolic acid (0.084 ± 0.086 to 0.555 ± 0.267 mg/g), dehydropachymic acid (0.205 ± 0.019 to 0.547 ± 0.051 mg/g), poricoic acid B (0.067 ± 0.028 to 0.409 ± 0.095 mg/g), while 16α-hydroxydehydrotrametenolic acid content was lowest (0.044 ± 0.017 to 0.090 ± 0.034 mg/g); of note, eburicoic acid was not detected. Pachymic acid began to accumulate at high concentrations during the early growth periods of Fushen, and the triterpenoid acid content varied across the nine growth periods. The content of the 12 triterpenoid acids increased during the growth period, as they were not detected in June, lower in August and September, further decreased in September and December, and the highest in March and April (Figure 5).

**Table 2:** The content levels (mg/g) of 13 triterpenoid acids in nine growth periods of Fushen.

**No**	**1**	**2**	**3**	**4**	**5**	**6**	**7**	**8**	**9**	**10**	**11**	**12**	**13**
Jun.	–	–	–	–	–	–	–	–	–	–	–	–	–
Aug.	0.063 ± 0.044	0.169 ± 0.083	0.367 ± 0.282	0.097 ± 0.008	0.342 ± 0.320	0.060 ± 0.022	0.131 ± 0.016	0.342 ± 0.069	0.205 ± 0.019	0.702 ± 0.026	0.253 ± 0.069	0.184 ± 0.038	–
Sep.	0.044 ± 0.017	0.136 ± 0.043	0.067 ± 0.028	0.192 ± 0.040	0.156 ± 0.103	0.051 ± 0.026	0.086 ± 0.030	0.225 ± 0.054	0.299 ± 0.076	0.701 ± 0.107	0.084 ± 0.086	0.086 ± 0.052	
Oct.	0.078 ± 0.008	0.285 ± 0.032	0.187 ± 0.024	0.234 ± 0.026	0.520 ± 0.038	0.153 ± 0.006	0.190 ± 0.014	0.671 ± 0.038	0.415 ± 0.020	1.391 ± 0.035	0.383 ± 0.100	0.281 ± 0.046	–
Nov.	0.057 ± 0.015	0.182 ± 0.06	0.187 ± 0.133	0.317 ± 0.084	0.777 ± 0.473	0.210 ± 0.042	0.133 ± 0.047	0.302 ± 0.111	0.483 ± 0.106	0.998 ± 0.106	0.332 ± 0.220	0.332 ± 0.112	–
Dec.	0.049 ± 0.009	0.141 ± 0.026	0.166 ± 0.039	0.222 ± 0.063	0.601 ± 0.166	0.235 ± 0.073	0.244 ± 0.208	0.295 ± 0.052	0.218 ± 0.162	0.937 ± 0.062	0.203 ± 0.020	0.255 ± 0.051	–
Jan.	0.079 ± 0.010	0.245 ± 0.072	0.378 ± 0.191	0.287 ± 0.037	0.702 ± 0.449	0.196 ± 0.073	0.303 ± 0.148	0.476 ± 0.134	0.457 ± 0.050	1.083 ± 0.251	0.455 ± 0.071	0.311 ± 0.072	–
Mar.	0.090 ± 0.034	0.249 ± 0.097	0.385 ± 0.091	0.330 ± 0.127	0.800 ± 0.217	0.309 ± 0.032	0.284 ± 0.029	0.470 ± 0.125	0.547 ± 0.051	1.385 ± 0.201	0.532 ± 0.089	0.223 ± 0.090	–
Apr.	0.086 ± 0.028	0.262 ± 0.025	0.409 ± 0.095	0.306 ± 0.052	0.885 ± 0.306	0.361 ± 0.046	0.223 ± 0.002	0.383 ± 0.003	0.462 ± 0.011	1.084 ± 0.064	0.555 ± 0.267	0.255 ± 0.067	–

*“–”: under LOQ; 1, 2, 3, 4, 5, 6, 7, 8, 9, 10, 11, 12, and 13 were represented 16α-hydroxydehydrotrametenolic acid, 16α-hydroxytrametenolic acid, poricoic acid B, dehydrotumulosic acid, poricoic acid A, polyporenic acid C, 3-o-acetyl-16α-hydroxydehydrotrametenolic acid, 3-o-acetyl-16α-hydroxytrametenolic acid, dehydropachymic acid, pachymic acid, dehydrotrametenolic acid, dehydroeburicoic acid, and eburicoic acid, respectively*.

### Accumulation of Specialized Metabolites in Four Parts of Fushen at Different Growth Periods

The specialized metabolites in the four parts of the Fushen also varied. Compounds were most abundant in the FP, as 102 were identified, while, in the BO, BI, and FM, there were 74, 95, and 94 compounds identified.

#### Multivariate Statistical Analysis

To understand the differences in the specialized metabolites in the different parts of the Fushen, a hierarchical clustering analysis heat map, PCA, PLS-DA, and OPLS-DA were performed separately for the FP, BO, BI, and FM. The hierarchical clustering analysis heat map for four parts of Fushen at different growth periods is shown in Figure 4B. The four parts were clearly classified into two clusters. Except for the August and September samples, all the FP samples were clustered into Group A. The samples from BO, BI, and FM were divided into Group B. Among the compounds, 39, 75, 103, 106, 109, 99, 41, 80, 83, 86, 47, 49, 54, 56, and 61 had higher levels of accumulation in most samples. Moreover, compounds 15, 21, 22, 50, 17, 37, 18, 114, 30, 93, 58, 29, 112, 107, 60, 87, 28, 34, 94, 102, 78, 89, 101, 36, 67, 24, 119, 53, 26, 16, 35, 38, 48, 72, 52, 45, 51, 59, 65, 27, 43, and 77 had higher levels of accumulation in the FP than the other three parts.

After Pareto scaling with mean centering, the results of the PCA were found to be consistent with those of the hierarchical clustering analysis heat map (Supplementary Figure 4C). However, as shown in Figure 4D, the four parts of the Fushen were divided into three groups by the PLS-DA (R^2^Y = 0.786, Q^2^ = 0.618). The FP samples were divided into Group A, except for the August and September FP samples. The BO and BI samples were divided into Group B, while the FM samples were divided into Group C. Thirty-nine specific markers were found to possess a high discrimination potential (VIP > 1). Compounds 2, 3, 4, 5, 6, 7, 9, 11, 12, 13, 14, 27, 28, 38, 48, 49, 51, 54, 59, 62, 68, 69, 71, 72, 73, 75, 76, 80, 86, 88, 99, 101, 103, 106, 109, 112, 114, 117, and 119 were tentatively identified (Supplementary Table 6). Moreover, in 200 rounds of random permutations, the R^2^ and Q^2^ intercepts were 0.146 and −0.284, respectively, indicating that the established PLS-DA model was validated without overfitting (Supplementary Figure 4D). To investigate the differences between the BO and BI, OPLS-DA was carried out, and the groups were further subdivided into B-I and B-II (Supplementary Figures 4E,F). Group B–I included all samples of BE, and Groups B–II included all BI samples. There were nine orthogonal X vectors in the OPLS-DA model.

#### Quantitative Analysis of Fushen in Four Parts at Nine Growth Periods

**Figure 6:**
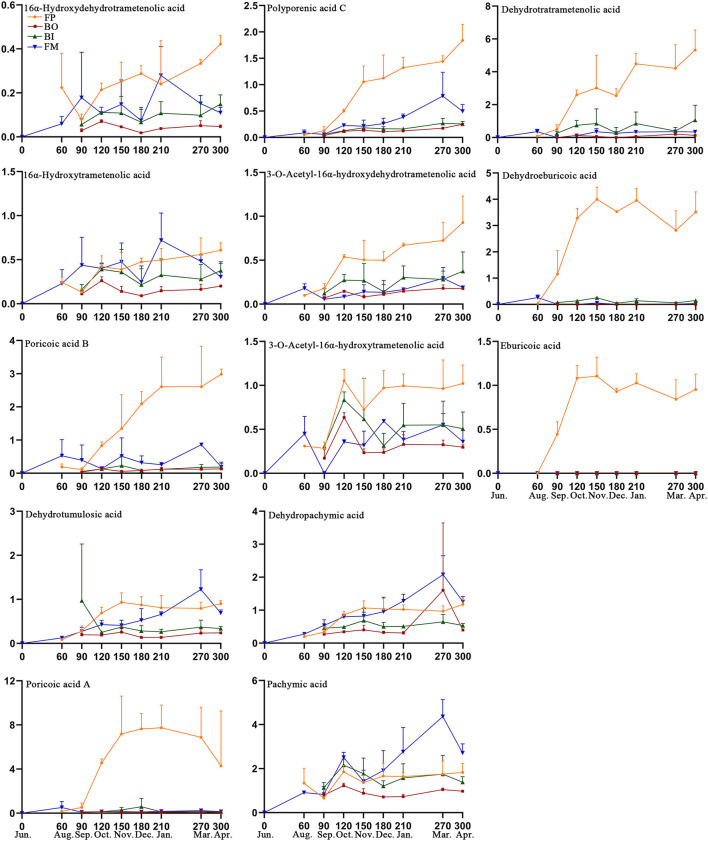
Content levels (mg/g) for 13 triterpenoid acids in the four parts of Fushen at nine growth periods.

The content levels of the 13 triterpenoid acids were determined in the 89 samples from the different parts of the Fushen using UPLC (Table 3). Triterpenoid acid was not detected in any of the four parts in June (Figure 6). The levels of 13 triterpenoid acids in the four parts were lowest in August and September. There was no significant difference in the content of the 13 triterpenoid acids in the four parts of Fushen in June, August, and September. After September, the overall trends for 16α-hydroxydehydrotrametenolic acid, poricoic acid B, poricoic acid A, polyporenic acid C, 3-o-acetyl-16α-hydroxydehydrotrametenolic acid, dehydrotrametenolic acid, and dehydroeburicoic acid followed the order FP > FM > BI > BO. The content of 16α-hydroxytrametenolic acid was lower in the BO, while that of the 3-o-acetyl-16α-hydroxytrametenolic acid was lower in the FM. Eburicoic acid was under the quantitative limited in most samples, except for the FP. In addition, the levels of pachymic acid (4.365 ± 0.773 mg/g), dehydropachymic acid (2.079 ± 0.573 mg/g), and dehydrotumulosic acid (1.223 ± 0.449 mg/g) were much higher in the FM in March when compared with the other three parts.

**Table 3:** The content levels (mg/g) of 13 triterpenoid acids in four parts of Fushen at the nine growth periods.

**No**	**1**	**2**	**3**	**4**	**5**	**6**	**7**	**8**	**9**	**10**	**11**	**12**	**13**
Jun.FM	–	–	–	–	–	–	–	–	–	–	–	–	–
Aug.FP	0.224 ± 0.156	0.242 ± 0.063	0.184 ± 0.094	0.083 ± 0.030	0.162 ± 0.079	0.049 ± 0.005	0.099 ± 0.014	0.311 ± 0.003	0.197 ± 0.079	1.339 ± 0.661	0.074 ± 0.034	0.036 ± 0.004	
Aug.FM	0.059 ± 0.034	0.229 ± 0.158	0.526 ± 0.486	0.125 ± 0.034	0.512 ± 0.543	0.087 ± 0.054	0.179 ± 0.051	0.450 ± 0.197	0.275 ± 0.021	0.905 ± 0.051	0.355 ± 0.147	0.258 ± 0.098	–
Sep.FP	0.080 ± 0.024	0.132 ± 0.038	0.111 ± 0.050	0.279 ± 0.116	0.530 ± 0.391	0.128 ± 0.074	0.179 ± 0.054	0.283 ± 0.071	0.330 ± 0.158	0.657 ± 0.000	0.494 ± 0.282	1.157 ± 0.893	0.445 ± 0.141
Sep.BO	0.029 ± 0.008	0.112 ± 0.024	0.035 ± 0.000	0.196 ± 0.031	0.091 ± 0.016	0.036 ± 0.007	0.069 ± 0.018	0.172 ± 0.099	0.271 ± 0.053	0.795 ± 0.089	–	–	–
Sep.BI	0.056 ± 0.027	0.162 ± 0.056	0.051 ± 0.014	0.971 ± 1.285	0.111 ± 0.017	0.058 ± 0.008	0.122 ± 0.057	0.305 ± 0.000	0.456 ± 0.061	1.135 ± 0.227	0.254 ± 0.132	0.073 ± 0.064	–
Sep.FM	0.178 ± 0.207	0.436 ± 0.316	0.393 ± 0.460	0.271 ± 0.092	0.090 ± 0.032	0.058 ± 0.017	0.056 ± 0.023	–	0.537 ± 0.170	0.830 ± 0.357	–	–	–
Oct.FP	0.160 ± 0.123	0.422 ± 0.120	0.828 ± 0.111	0.692 ± 0.129	4.570 ± 0.356	0.498 ± 0.037	0.538 ± 0.026	1.053 ± 0.126	0.863 ± 0.088	1.852 ± 0.288	2.600 ± 0.288	3.282 ± 0.368	1.082 ± 0.145
Oct.BO	0.070 ± 0.008	0.261 ± 0.045	0.128 ± 0.038	0.188 ± 0.013	0.121 ± 0.051	0.118 ± 0.025	0.146 ± 0.003	0.634 ± 0.055	0.344 ± 0.015	1.221 ± 0.110	0.121 ± 0.027	–	–
Oct.BI	0.111 ± 0.006	0.376 ± 0.035	0.128 ± 0.021	0.252 ± 0.022	0.133 ± 0.022	0.126 ± 0.021	0.274 ± 0.063	0.835 ± 0.090	0.492 ± 0.034	2.160 ± 0.268	0.740 ± 0.296	0.137 ± 0.046	–
Oct.FM	0.107 ± 0.028	0.400 ± 0.062	0.134 ± 0.061	0.427 ± 0.096	0.132 ± 0.025	0.229 ± 0.011	0.085 ± 0.007	0.359 ± 0.023	0.799 ± 0.075	2.508 ± 0.229	0.111 ± 0.019	–	–
Nov.FP	0.251 ± 0.088	0.389 ± 0.194	1.346 ± 1.022	0.931 ± 0.215	7.131 ± 3.510	1.052 ± 0.303	0.503 ± 0.223	0.726 ± 0.356	1.067 ± 0.215	1.362 ± 0.340	3.007 ± 1.993	3.997 ± 0.462	1.105 ± 0.215
Nov.BO	0.046 ± 0.002	0.139 ± 0.058	0.056 ± 0.016	0.257 ± 0.119	0.101 ± 0.030	0.134 ± 0.035	0.080 ± 0.023	0.234 ± 0.044	0.402 ± 0.139	0.882 ±± 0.208	0.066 ± 0.030	–	–
Nov.BI	0.109 ± 0.075	0.359 ± 0.257	0.229 ± 0.272	0.372 ± 0.154	0.299 ± 0.218	0.183 ± 0.066	0.268 ± 0.195	0.617 ± 0.464	0.684 ± 0.257	1.772 ± 0.705	0.852 ± 0.888	0.251 ± 0.069	–
Nov.FM	0.148 ± 0.111	0.474 ± 0.215	0.512 ± 0.553	0.410 ± 0.114	0.147 ± 0.081	0.212 ± 0.117	0.14 ± 0.081	0.318 ± 0.161	0.818 ± 0.227	1.430 ± 0.496	0.349 ± 0.202	0.052 ± 0.000	–
Dec.FP	0.288 ± 0.036	0.474 ± 0.042	2.093 ± 0.370	0.874 ± 0.187	7.644 ± 1.387	1.124 ± 0.438	0.499 ± 0.098	0.971 ± 0.197	1.034 ± 0.314	1.661 ± 0.575	2.546 ± 0.46	3.533 ± 0.033	0.930 ± 0.038
Dec.BO	0.019 ± 0.001	0.091 ± 0.003	0.082 ± 0.001	0.137 ± 0.026	0.104 ± 0.011	0.123 ± 0.021	0.111 ± 0.018	0.239 ± 0.039	0.322 ± 0.042	0.711 ± 0.058	–	–	–
Dec.BI	0.067 ± 0.068	0.214 ± 0.183	0.086 ± 0.031	0.283 ± 0.126	0.586 ± 0.748	0.163 ± 0.039	0.141 ± 0.081	0.313 ± 0.142	0.503 ± 0.126	1.210 ± 0.239	0.283 ± 0.326	0.043 ± 0.000	–
Dec.FM	0.074 ± 0.051	0.246 ± 0.184	0.314 ± 0.206	0.523 ± 0.267	0.118 ± 0.036	0.262 ± 0.103	0.134 ± 0.133	0.593 ± 0.000	0.950 ± 0.442	1.910 ± 0.906	0.255 ± 0.000	–	–
Jan.FP	0.240 ± 0.197	0.495 ± 0.133	2.609 ± 0.893	0.808 ± 0.280	7.748 ± 2.062	1.321 ± 0.196	0.671 ± 0.024	0.994 ± 0.134	1.021 ± 0.137	1.638 ± 0.172	4.486 ± 0.634	3.957 ± 0.460	1.027 ± 0.105
Jan.BO	0.038 ± 0.000	0.147 ± 0.046	0.121 ± 0.054	0.136 ± 0.009	0.082 ± 0.031	0.123 ± 0.021	0.147 ± 0.032	0.328 ± 0.110	0.309 ± 0.043	0.730 ± 0.102	0.064 ± 0.054	–	–
Jan.BI	0.108 ± 0.053	0.326 ± 0.184	0.118 ± 0.029	0.264 ± 0.057	0.130 ± 0.045	0.162 ± 0.021	0.303 ± 0.132	0.547 ± 0.250	0.508 ± 0.054	1.575 ± 0.644	0.849 ± 0.704	0.141 ± 0.074	–
Jan.FM	0.279 ± 0.132	0.719 ± 0.311	0.256 ± 0.064	0.659 ± 0.059	0.162 ± 0.032	0.384 ± 0.057	0.165 ± 0.019	0.382 ± 0.094	1.277 ± 0.205	2.761 ± 1.103	0.329 ± 0.117	–	–
Mar.FP	0.334 ± 0.018	0.560 ± 0.188	2.609 ± 1.221	0.793 ± 0.140	6.872 ± 2.728	1.441 ± 0.107	0.725 ± 0.207	0.963 ± 0.325	0.967 ± 0.162	1.746 ± 0.594	4.207 ± 1.433	2.818 ± 0.745	0.842 ± 0.222
Mar.BO	0.051 ± 0.022	0.163 ± 0.059	0.121 ± 0.039	0.235 ± 0.099	0.106 ± 0.034	0.175 ± 0.015	0.180 ± 0.038	0.324 ± 0.054	1.601 ± 2.044	1.012 ± 0.014	0.202 ± 0.060	–	–
Mar.BI	0.099 ± 0.060	0.279 ± 0.166	0.174 ± 0.088	0.369 ± 0.156	0.160 ± 0.068	0.268 ± 0.092	0.281 ± 0.095	0.551 ± 0.269	0.646 ± 0.222	1.749 ± 0.847	0.394 ± 0.226	0.062 ± 0.048	–
Mar.FM	0.151 ± 0.037	0.482 ± 0.072	0.860 ± 0.018	1.223 ± 0.449	0.239 ± 0.072	0.784 ± 0.450	0.294 ± 0.123	0.551 ± 0.129	2.079 ± 0.573	4.365 ± 0.773	0.354 ± 0.047	0.034 ± 0.000	–
Apr.FP	0.422 ± 0.038	0.608 ± 0.083	2.985 ± 0.151	0.902 ± 0.066	4.287 ± 4.975	1.838 ± 0.301	0.930 ± 0.301	1.023 ± 0.210	1.175 ± 0.182	1.828 ± 0.403	5.321 ± 1.22	3.517 ± 0.763	0.953 ± 0.174
Apr.BO	0.047 ± 0.007	0.200 ± 0.007	0.129 ± 0.007	0.239 ± 0.020	0.079 ± 0.015	0.248 ± 0.003	0.177 ± 0.006	0.298 ± 0.028	0.399 ± 0.001	0.972 ± 0.001	0.131 ± 0.010	–	–
Apr.BI	0.149 ± 0.042	0.377 ± 0.080	0.185 ± 0.117	0.336 ± 0.044	0.139 ± 0.062	0.256 ± 0.049	0.375 ± 0.220	0.508 ± 0.189	0.534 ± 0.063	1.384 ± 0.248	1.056 ± 0.907	0.148 ± 0.000	–
Apr.FM	0.110 ± 0.024	0.304 ± 0.171	0.189 ± 0.142	0.690 ± 0.070	0.145 ± 0.031	0.496 ± 0.129	0.189 ± 0.018	0.358 ± 0.128	1.255 ± 0.156	2.707 ± 0.414	0.337 ± 0.107	0.036 ± 0.000	–

*“–”: under LOQ; 1, 2, 3, 4, 5, 6, 7, 8, 9, 10, 11, 12, and 13 were represented 16α-hydroxydehydrotrametenolic acid, 16α-hydroxytrametenolic acid, poricoic acid B, dehydrotumulosic acid, poricoic acid A, polyporenic acid C, 3-o-acetyl-16α-hydroxydehydrotrametenolic acid, 3-o-acetyl-16α-hydroxytrametenolic acid, dehydropachymic acid, pachymic acid, dehydrotrametenolic acid, dehydroeburicoic acid, and eburicoic acid, respectively*.

## Discussion

### Dynamic Changes in Specialized Metabolites of Fushen at Different Growth Periods

In recent years, identification and quality control of Fushen have been ongoing areas of research. Fushen has been shown to contain triterpene acids, polysaccharides, sterols, and other active ingredients, and the methods of chromatography and mass spectrometry for this species have gradually improved. Wang et al. identified 27 triterpenoid acids from Fushen (Wang et al., 2015), while Zou et al. preliminarily identified 62 triterpenoid acids in *P. cocos* using UPLC-Q/TOF-MS (Zou et al., 2019). Zhu et al. established a fingerprint method to study the quality of *P. cocos* from different areas using UPLC-QQQ-MS (Zhu et al., 2018a). LC-MS has the merits of high resolution, high sensitivity, and high throughput in that it can detect a wide range of metabolites in short analysis times, and, consequently, LC-MS has certain advantages in the study of Fushen metabolomics. In this study, 119 specialized metabolites were identified in Fushen, but, unfortunately, 35 of the compounds were unknown; however, previous reports have also found unknown compounds in Fushen (Zhu et al., 2018b). Some traced triterpene acids were only tentatively identified according to the quasi-molecular ions and the retention capabilities, owing to the deficiencies of the corresponding reference standards, literature, and the lack of available high-quality MS/MS spectra. This is a clear indication that the compounds in Fushen require further investigation. In addition, because of the complexity of the compounds in Fushen, and the difficulty in separating and purifying the triterpenoid acids, it is difficult to quantify multiple compounds simultaneously. Thirteen triterpenoid acids were simultaneously determined for the first time using UPLC in this study, and they were identified as the major compounds in Fushen (Sun et al., 2019; Deng et al., 2020).

The dynamic changes in the specialized metabolites of Fushen at different growth periods have not previously been investigated. Market survey data and sample origin information showed that the harvest time for Fushen was not fixed. However, there is a close relationship between the harvest time and the resultant quality of the derived traditional Chinese medicines. There are also differences in the effective compounds during different growth periods (Gao, 2015). In our study, there were obvious differences in the specialized metabolites of the Fushen in the nine growth periods. The results showed that the accumulation of 15 unknown compounds in the Fushen samples from June was higher than in the other growth periods. The main reason for the differences in the specialized metabolites was that the samples collected in June had not yet been inoculated with mycelia, and all compounds were detected in young pine roots. In the markets, the artificial insertion of young pine roots into Fuling to create false Fushen has been reported. The data reported in this investigation will help to distinguish the growth periods of Fushen but also provide a method for identifying adulterated Fushen in the market. Additionally, 32 potential growth-period-specific markers were identified. Untargeted metabolomics in different growth periods revealed that the specialized metabolites of Fushen were affected by different harvest periods.

The triterpenoid acids are some of the most effective compounds in Fushen and include pachymic acid, dehydropachymic acid, polyporenic acid C, dehydrotrametenolic acid, poricoic acid A, and poricoic acid B (Zhao et al., 2020). The triterpenoid acid content in the different growth periods of Fushen was also different. The content of eburicoic acid in the 26 Fushen samples was lower than that in the LQDs, and it was mainly found in the epidermis of the Fushen (Zhu et al., 2018b). However, the proportion of the epidermis in the transverse section area in Fushen was smaller, so the content of the eburicoic acid was lower in all Fushen samples. Except in June, every growth period displayed higher content levels for pachymic acid and poricoic acid A than the other triterpenoid acids. This is an interesting finding that requires further investigation. Furthermore, the triterpenoid acid content was lower in June, August, and September, and higher in January, March, and April. Fushen is a saprophytic fungus that requires a special environment (Cheng et al., 2021a). The mycelia grow rapidly at approximately 25°C (Cheng et al., 2021a), and previous studies have reported that they grow faster and accumulate more biomass as lower triterpenoid acids are accumulating (Hu et al., 2017). The mean temperatures of Jinzhai, Anhui Province in June, August, and September 2019 are approximately 25, 26, and 22°C, respectively. Mean temperature began to decrease in October 2019. As temperature affects the mycelia growth rate of Fushen, it may also affect the accumulation of triterpenoid acid. Combined with untargeted metabolomics and targeted quantitative analysis results, the specialized metabolites of Fushen were abundant, and the main triterpenoid acids had high accumulation levels after January. However, different parts of the Fushen, from the outside to the inside, had different therapeutic applications. It is necessary to further explore the effect of the growth period on the compounds of the different parts of Fushen, in part to help determine a suitable harvest time.

### Distribution of Fushen Specialized Metabolites in Different Parts at Different Growth Periods

Fushen is a well-known traditional Chinese medicine that has been used for millennia as a medicinal and edible resource. Different parts of the Fushen have different pharmacological effects and clinical applications. Previous studies have confirmed the differences in compounds in different parts of Fushen (Wang et al., 2015). However, no conclusive specialized metabolites analysis for the different parts at growth periods of Fushen has previously been reported. In this study, untargeted metabolomics analysis showed that the FP, BO, BI, and FM of the Fushen at the nine growth periods were divided into four categories. This indicated that spatial variations had a greater effect than temporal variations on the specialized metabolites in Fushen. The distribution of the specialized metabolites in the FP, BO, BI, and FM of the Fushen was specific, and the 39 specific markers selected could be used to distinguish the different parts of Fushen. In addition, the specialized metabolites were more abundant in the FP than the other three parts. This was consistent with previous research that found that specialized metabolites were more abundant in the epidermis (Wang et al., 2015; Zhu et al., 2018b). Thus, the Fushen epidermis is a promising raw material for products.

Zhu et al. reported a higher content of dehydroeburicoic acid, polyporenic acid C, dehydrotrametenolic acid, and eburicoic acid in fulingpi, while pachymic acid was higher in fushenmu (Zhu et al., 2018b). Wang et al. demonstrated that the content of the dehydroeburicoic acid was 16-fold higher in fulingpi than in baifuling (Wang et al., 2015). Meng et al. also found a rich pachymic acid in fushenmu (Meng et al., 2015). Pharmacological and preclinical studies have revealed that pachymic acid has sedative-hypnotic effects, while dehydroeburicoic acid, polyporenic acid C, dehydrotrametenolic acid, and eburicoic acid are reported to have diuretic effects (Zhao et al., 2012; Feng et al., 2013; Shah et al., 2014). Therefore, the treatment of different diseases using different parts of Fushen is closely related to these triterpenoid acids. From our results, beginning in October, FP was found to be the key site for the accumulation of triterpenoid acids, including 16α-hydroxydehydrotrametenolic acid, poricoic acid B, poricoic acid A, polyporenic acid C, 3-o-acetyl-16α-hydroxydehydrotrametenolic acid, dehydrotrametenolic acid, dehydroeburicoic acid, and eburicoic acid. In March, pachymic acid, dehydropachymic acid, and dehydrotumulosic acid are mainly stored in the FM. The data determined in this study not only lend sufficient support to the above literature but also determine the influence of the growth period on the triterpenoid acid content in four parts of Fushen. However, some studies have found that dehydropachymic acid and dehydrotumulosic acid contents are higher in the fulingpi (Zhang et al., 2019). This could be caused by different harvest times, according to the experimental results. The BO and BI also had differences in their compounds as BI is close to FM, which is affected by the compounds of FM. Furthermore, previous studies reported differences between fulingpi and baifiling in expression of genes, which are putatively associated with secondary metabolite production (Wang, 2015). The expression levels of gene comp239634_c0 and gene comp18705_c0 in fulingpi were higher than those in baifuling. These genes were positively correlated with the contents of polyporhinic acid C and poricoic acid B. Certainly, further research is needed to elucidate the mechanism. As mentioned above, although the triterpenoid acid content of fulingpi was higher in the early growth periods, the triterpenoid acid content of fushenmu was higher in the late growth periods. From the perspective of the compounds in the four parts, Fushen should ideally be harvested in March, that is, after 270 days of growth.

Potential differential markers and bioactive compounds associated with the temporal and spatial variations of specialized metabolites in Fushen were identified. These results provide valuable reference data to help determine the harvesting time, perform quality evaluations, and assess the therapeutic applications of Fushen. Nevertheless, this study was limited as the uncertain metabolites were not identified, and, thus, some of the detail of the metabolites in Fushen is missing. Furthermore, the biosynthetic pathways of the differential markers and the other main active ingredients require further study.

## Conclusion

In this study, untargeted metabolomics and targeted quantitative approaches were utilized for the first time to characterize the temporal and spatial variations that occur in the accumulation of Fushen-specialized metabolites. A total of 119 specialized metabolites were identified and characterized; their content levels were found to vary dramatically among the nine growth periods assessed, and the highest accumulations occurred after January. Furthermore, the specialized metabolites showed different distributions in the four parts of the Fushen. The specialized metabolites were most abundant in the FP, indicating that FP is a promising raw material for products. In March, pachymic acid, dehydropachymic acid, and dehydrotumulosic acid were found to be mainly stored in the FM. Based on the close relationship between the compounds and their pharmacology, we have suggested that the most suitable harvest time for the Fushen is in March, that is, after 270 days of growth. These results have increased our understanding of the differential accumulation and distribution of specialized metabolites in the Fushen, during different growth periods. Taken together, our study provides a valuable reference for the future utilization of Fushen.

## Data Availability Statement

The original contributions presented in the study are included in the article/Supplementary Material, further inquiries can be directed to the corresponding author/s.

## Author Contributions

MY and HP conceived and designed the experiments. MY, YQ, and ZY performed the experiments. MY wrote the manuscript. MY, YZ, and RX conducted the data analyses. YZ and HP revised the manuscript. All authors contributed to the article and approved the submitted version.

## Funding

This study was supported by the National Key Research and Development Program of China (Grant No. 2017YFC1701601), the Major Increase and Reduction Project at the Central Level (No. 2060302), and the CAMS Innovation Fund for Medical Sciences (2019-I2M-5-065).

## Conflict of Interest

The authors declare that the research was conducted in the absence of any commercial or financial relationships that could be construed as a potential conflict of interest.

## Publisher's Note

All claims expressed in this article are solely those of the authors and do not necessarily represent those of their affiliated organizations, or those of the publisher, the editors and the reviewers. Any product that may be evaluated in this article, or claim that may be made by its manufacturer, is not guaranteed or endorsed by the publisher.

## References

[B1] Akihisa T. Uchiyama E. Kikuchi T. Tkuda H. Suzuki T. Kimura Y. (2009). Anti-tumor-promoting effects of 25-methoxyporicoic acid A and other triterpene acids from *Poria cocos*. J. Nat. Prod. 72, 1786–1792. 10.1021/np9003239 19746919

[B2] Bazargani M. M. Falahati-Anbaran M. Rohloff J. (2021). Comparative analyses of phytochemical variation within and between congeneric species of willow herb, *Epilobium hirsutum* and *E. parviflorum*: contribution of environmental factors. Front. Plant Sci. 11, 595190. 10.3389/fpls.2020.595190 33679815 PMC7925418

[B3] Chang X. W. Zhang J. J. Li D. K. Zhou D. Z. Zhang Y. L. Wang J. C.. (2017). Nontargeted metabolomics approach for the differentiation of cultivation ages of mountain cultivated ginseng leaves using UHPLC/QTOF-MS. J. Pharm. Biomed. Anal. 141, 108–122. 10.1016/j.jpba.2017.04.009 28437718

[B4] Chao C. L. Huang H. W. Su M. H. Lin H. C. Wu W. M. (2021). The lanostane triterpenoids in *Poria cocos* play beneficial roles in immunoregulatory activity. Life. 11:111. 10.3390/life11020111 33535602 PMC7912843

[B5] Chatterjee N. S. Chevallier O. P. Wielogorska E. Black C. Elliott C. T. (2019). Simultaneous authentication of species identity and geographical origin of shrimps: untargeted metabolomics to recurrent biomarker ions. J. Chromatogr. A 1599, 75–84. 10.1016/j.chroma.2019.04.001 30967241

[B6] Chen B. S. Zhang J. J. Han J. J. Zhao R. L. Bao L. Huang Y.. (2019). Lanostane triterpenoids with glucose-uptake-stimulatory activity from peels of the cultivated edible mushroom *Wolfiporia cocos*. J. Agric. Food Chem. 26, 7348–7364. 10.1021/acs.jafc.9b02606 31180673

[B7] Cheng M. E. Yang M. Yin M. Z. Jing Z. X. Peng H. S. Shi T. T.. (2021a). Suitable planting area of *Poria cocos* in Jinzhai county of Dabie Mountains region. Chin. J. Chin. Mater. Med. 46, 260–266. 10.19540/j.cnki.cjcmm.20201024.102 33645110

[B8] Cheng Y. Xie Y. Ge J. C. Wang L. Pen D. Y. Yu N. J.. (2021b). Structural characterization and hepatoprotective activity of a galactoglucan from *Poria cocos*. Carbohydr. Polym. 263, 117979. 10.1016/j.carbpol.2021.117979 33858575

[B9] Cho H. W. Kim S. B. Jeong M. K. Park Y. Miller N. G. Ziegler T. R.. (2008). Discovery of metabolite features for the modelling and analysis of high-resolution NMR spectra. Int. J. Data Min. Bioinform. 2, 176–192. 10.1504/IJDMB.2008.019097 18767354 PMC3883573

[B10] Committee for the Pharmacopoeia of PR China. (2020). Pharmacopoeia of PR China, Part I. Beijing: China Medical Science and Technology Press.

[B11] Deng T. M. Peng D. Y. Yu N. J. Wang L. Zhang Y. Ding Z. X.. (2020). Research progress on chemical composition and pharmacological effects of *Poriacocos* and predictive analysis on quality markers. Chin. Tradit. Herbal Drugs. 51, 2703–2717. 10.7501/j.issn.0253-2670.2020.10.013

[B12] Feng G. F. Zheng Y. Sun Y. F. Liu S. Pi Z. F. Song F. R.. (2018). A targeted strategy for analyzing untargeted mass spectral data to identify lanostaneetype triterpene acids in *Poria cocos* by integrating a scientific information system and liquid chromatography and mass spectrometry combined with ion mobility spectrometry. Anal. Chim. Acta. 1033, 87–99. 10.1016/j.aca.2018.06.048 30172336

[B13] Feng Y. L. Lei P. Tian T. Yin L. Chen D. Q. Chen H.. (2013). Diureticactivity of some fractions of the epidermis of *Poria cocos*. J. Ethnopharmacol. 150, 1114–1118. 10.1016/j.jep.2013.10.043 24184192

[B14] Gao X. M. (2015). Traditional Chinese Medicine. Beijing: China Press of Traditional Chinese Medicine.

[B15] Hu G. S. Huang C. G. Zhang Y. Xiao W. Jia J. M. (2017). Accumulation of biomass and four triterpenoids in two-stage cultured *Poria cocos* mycelia and diuretic activity in rats. Chin. J. Nat. Med. 15, 265–270. 10.1016/S1875-5364(17)30043-2 28527511

[B16] Hu Z. H. (2014). Relationship Between Structure, Development and Medicinal Components of Medicinal Plants. Shanghai: Shanghai Scientific and Technical Publishers.

[B17] Jiang Y. Fan L. P. (2021). The effect of *Poria cocos* ethanol extract on the intestinal barrier function and intestinal microbiota in mice with breast cancer. J. Ethnopharmacol. 266:113456. 10.1016/j.jep.2020.113456 33039631

[B18] Kang C. Lai C. J. Zhao D. Zhou T. Liu D. H. Lv C.. (2017). A practical protocol for comprehensive evaluation of sulfur-fumigation of Gastrodia Rhizoma using metabolome and health risk assessment analysis. J. Hazard. Mater. 340, 221–230. 10.1016/j.jhazmat.2017.07.003 28715745

[B19] Li K. (2013). Isolation, Purification, Structure Elucidation of Triterpenoids from Surface Layer of Poria cocos and Study on Fingerprints of Traditional Chinese Medicine Poria cocos (Schw.) Wolf. Hubei: Hubei University of Chinese Medicine.

[B20] Li T. H. Hou C. C. Chang C. L. T. Yang W. C. (2011). Anti-hyperglycemic properties of crude extract and triterpenes from *Poria cocos*. Evid. Based Complement. Alternat. Med. 2011:128402. 10.1155/2011/128402 20924500 PMC2949581

[B21] Li W. F. Yu J. J. Zhao J. M. Xiao X. Li W. Q. Zang L. L.. (2021). *Poria cocos* polysaccharides reduces high-fat diet-induce darteriosclerosis in ApoE^−/−^ mice by inhibiting inflammation. Phytother. Res. 35, 2220–2229. 10.1002/ptr.6980 33350533

[B22] Liang D. L. Yong T. Q. Diao X. Chen S. D. Chen D. L. Xiao C.. (2021). Hypouricaemic and nephroprotective effects of *Poria cocos* in hyperuricemic mice by up-regulating ATP-binding cassette super-family G member 2. Pharm. Biol. 59, 275–286. 10.1080/13880209.2021.1885450 33651969 PMC7928048

[B23] Meng L. Nie L. Shen J. Y. Wang G. Z. (2015). Analysis of pachymic acid and total terpene constituents of Poria, Poria cum Pini Radix, Pini Radix in Poria and Pini Radix. J. Hubei Univ. Chin. Med. 17, 40–43. 10.3969/j.issn.1008-987x.2015.05.14

[B24] Pusztahelyi T. Holb I. J. Pócsi I. (2015). Secondary metabolites in fungus-plant interactions. Front. Plant Sci. 6:573. 10.3389/fpls.2015.00573 26300892 PMC4527079

[B25] Schymanski E. L. Jeon J. Gulde R. Fenner K. Ruff M. Singer H. P.. (2014). Identifying small molecules via high resolution mass spectrometry: communicating confidence. Environ. Sci. Technol. 48, 2097–2098. 10.1021/es5002105 24476540

[B26] Shah V. K. Choi J. J. Han J. Y. Lee M. K. Hong J. T. Oh K. W. (2014). Pachymic acid enhances pentobarbital-induced sleeping behaviors via GABA_A_-ergic systems in mice. Biomol. Ther. 22, 314–320. 10.4062/biomolther.2014.045 25143810 PMC4131518

[B27] Sun Y. F. Zhen X. Y. Liu T. S. Liu S. Liu Z. Y. Song F. R.. (2019). Discovery of quality markers and quality evaluation of *Poria cocos* based on “*invitro-in vivo*” multidimensional chemical group associated network. Chin. Tradit. Herbal Drugs. 50, 4562–4568. 10.7501/j.issn.0253-2670.2019.19.006

[B28] Wang H. X. (2016). Qualitative and Quantitative Analysis of Components in Different Medicinal Parts of Poria and Study on Quality Standard of Rubra Poria. Hebei: Hebei Medical University.

[B29] Wang K. F. (2014). Study on Chemical Constituents and Quality Control of Poria cocos. Beijing: Beijing University of Chinese Medicine.

[B30] Wang W. H. (2015). Triterpene Acid Comparison Study in Different Part of Poria cocos (Schw.) Wolf and the Mechanism Investigation Base on LC-MS and Transcriptome Analysis. Beijing: China Academy of Chinese Medical Sciences.

[B31] Wang W. H. Dong H. J. Yan R. Y. Li H. Li P. Y. Chen P.. (2015). Comparative study of lanostane-type triterpene acids in differentparts of *Poria cocos* (Schw.) Wolf by UHPLC-Fourier transform MS and UHPLC-triple quadruple MS. J. Pharm. Biomed. Anal. 102, 203–214. 10.1016/j.jpba.2014.09.014 25282601

[B32] Wu F. Li S. J. Dong C. H. Dai Y. C. Papp V. (2020a). The genus *Pachyma* (Syn. *Wolfiporia*) reinstated and species clarification of the cultivated medicinal mushroom “Fuling” in China. Front. Microbiol. 11:590788. 10.3389/fmicb.2020.590788 33424793 PMC7793888

[B33] Wu P. Tan H. Y. Zhan J. F. Wang W. X. Hu T. Li S. M. (2020b). Optimization of bioprocess extraction of *Poria cocos* polysaccharide (PCP) with *Aspergillus niger* β-glucanase and the evaluation of PCP antioxidant property. Molecules. 25:5930. 10.3390/molecules25245930 33333769 PMC7765248

[B34] Zhang G. H. Wang H. X. Xie W. Y. Wang Q. Wang X. Wang C. Y.. (2019). Comparison of triterpene compounds of four botanical parts from *Poria cocos*(Schw.) wolf using simultaneous qualitative and quantitative method and metabolomics approach. Food Res. Int. 121, 666–677. 10.1016/j.foodres.2018.12.036 31108794

[B35] Zhao Q. L. Zhang L. Bian X. K. Qian D. W. Guo S. Yan H.. (2020). Analysis of 8 triterpene acids in Poria from different habitats based on UPLC-QTRAP-MS. Chin. J. Pharm. Anal. 40, 1169–1177. 10.16155/j.0254-1793.2020.07.04

[B36] Zhao Y. J. Chu S. S. Gui S. Y. Qin Y. J. Xu R. Shan T. Y.. (2021). Tissue-specific metabolite profiling of *Fallopia multiflora* (Heshouwu) and *Fallopia multiflora* var. *angulata* by mass spectrometry imaging and laser microdissection combined with UPLC-Q/TOF-MS. J. Pharm. Biomed. Anal. 200:114070. 10.1016/j.jpba.2021.114070 33878622

[B37] Zhao Y. Y. Feng Y. L. Du X. Xi Z. H. Cheng X. L. Wei F. (2012). Diuretic activity of the ethanol and aqueous extracts of the surface layer of *Poria cocos* in rat. J. Ethnopharmacol. 144, 775–778. 10.1016/j.jep.2012.09.033 23058989

[B38] Zheng Y. Yang X. W. (2008). Poriacosones A and B: two new lanostane triterpenoids from *Poria cocos*. J. Asian Nat. Prod. Res. 10, 645–651. 10.1080/10286020802133597 18636376

[B39] Zhu L. X. Wang X. Li S. C Qi E. R. Meng J. Ching Lam K. Y.. (2020). Qualitative and quantitative characterization of carbohydrate profilesin three different parts of *Poria cocos*. J. Pharm. Biomed. Anal. 179:113009. 10.1016/j.jpba.2019.113009 31816475

[B40] Zhu L. X. Xu J. Wang R. J. Li H. X. Tan Y. Z. Chen H. B.. (2018a). Correlation between quality and geographicalorigins of *Poria cocos* revealed by qualitative fingerprint profiling and quantitative determination of triterpenoid acids. Molecules. 23:2200. 10.3390/molecules23092200 30200284 PMC6225149

[B41] Zhu L. X. Xu J. Zhang S. J. Huang Q. Wang R. J. Chen H. B.. (2018b). Qualitatively and quantitatively comparing secondary metabolites in three medicinal parts derived from *Poria cocos* (Schw.) Wolf using UHPLC-QTOF-MS/MS-based chemical profiling. J. Pharm. Biomed. Anal. 150, 278–286. 10.1016/j.jpba.2017.11.066 29258047

[B42] Zou Y. T. Long F. Wu C. Y. Zhou J. Zhang W. Xu J. D.. (2019). A dereplication strategy for identifying triterpene acid analogues in *Poria cocos* by comparing predicted and acquired UPLC-ESI-QTOF-MS/MS data. Phytochem. Anal. 30, 292–310. 10.1002/pca.2813 30569602

