# Supplementary material for: Untargeted Metabolomics and Targeted Quantitative Analysis of Temporal and Spatial Variations in Specialized Metabolites Accumulation in Poria cocos (Schw.) Wolf (Fushen)
Source: Front Plant Sci. 2021 Sep 21;12:713490. doi: 10.3389/fpls.2021.713490 (PMC8490877; doi:10.3389/fpls.2021.713490)
Supplement: Supplementary file 1 [file Data_Sheet_1.docx]

**Supplementary Material**

**Supplementary Figures**

Supplementary Figure 1. Environment of field of Fushen from nine growth periods (-: no found).

Supplementary Figure 2. Transverse section segmentation of four parts of Fushen.

Supplementary Figure 3. The MS data and structures of the 13 reference compounds.

Supplementary Figure 4. PCA score plot of nine growth periods (A), permutation plot (after 200 times) of PLS-DA model of nine growth periods (B), PCA score plot of four parts (C), permutation plot (after 200 times) of PLS-DA model of four parts (D), OPLS-DA score plot of two parts (E), permutation plot (after 200 times) of OPLS-DA model of two parts (F) of Fushen based on UPLC-Q/TOF-MS in ESI^-^.

Supplementary Figure 5. The UPLC chromatogram of thirteen compounds of Fushen. (1) 16α-hydroxydehydrotrametenolic acid, (2) 16α-hydroxytrametenolic acid, (3) poricoic acid B, (4) dehydrotumulosic acid, (5) poricoic acid A, (6) polyporenic acid C, (7) 3-o-acetyl-16α-hydroxydehydrotrametenolic acid, (8) 3-o-acetyl-16α-hydroxytrametenolic acid, (9) dehydropachymic acid, (10) pachymic acid, (11) dehydrotrametenolic acid, (12) dehydroeburicoic acid, (13) eburicoic acid.

**Supplementary Tables**

Supplementary Table 1. Collection information of Fushen from nine growth periods.

Supplementary Table 2. Collection information from four parts of Fushen at nine growth periods.

Supplementary Table 3. Characterization of specialized metabolites of Fushen by UPLC-Q/TOF-MS in ESI^+^.

Supplementary Table 4. Calibration curves, LOD, LOQ, precision, stability, repeatability and recovery of thirteen standard compounds.

Supplementary Table 5. Potential markers for the differentiation of nine growth periods of Fushen.

Supplementary Table 6. Potential markers for the differentiation of four parts of Fushen.


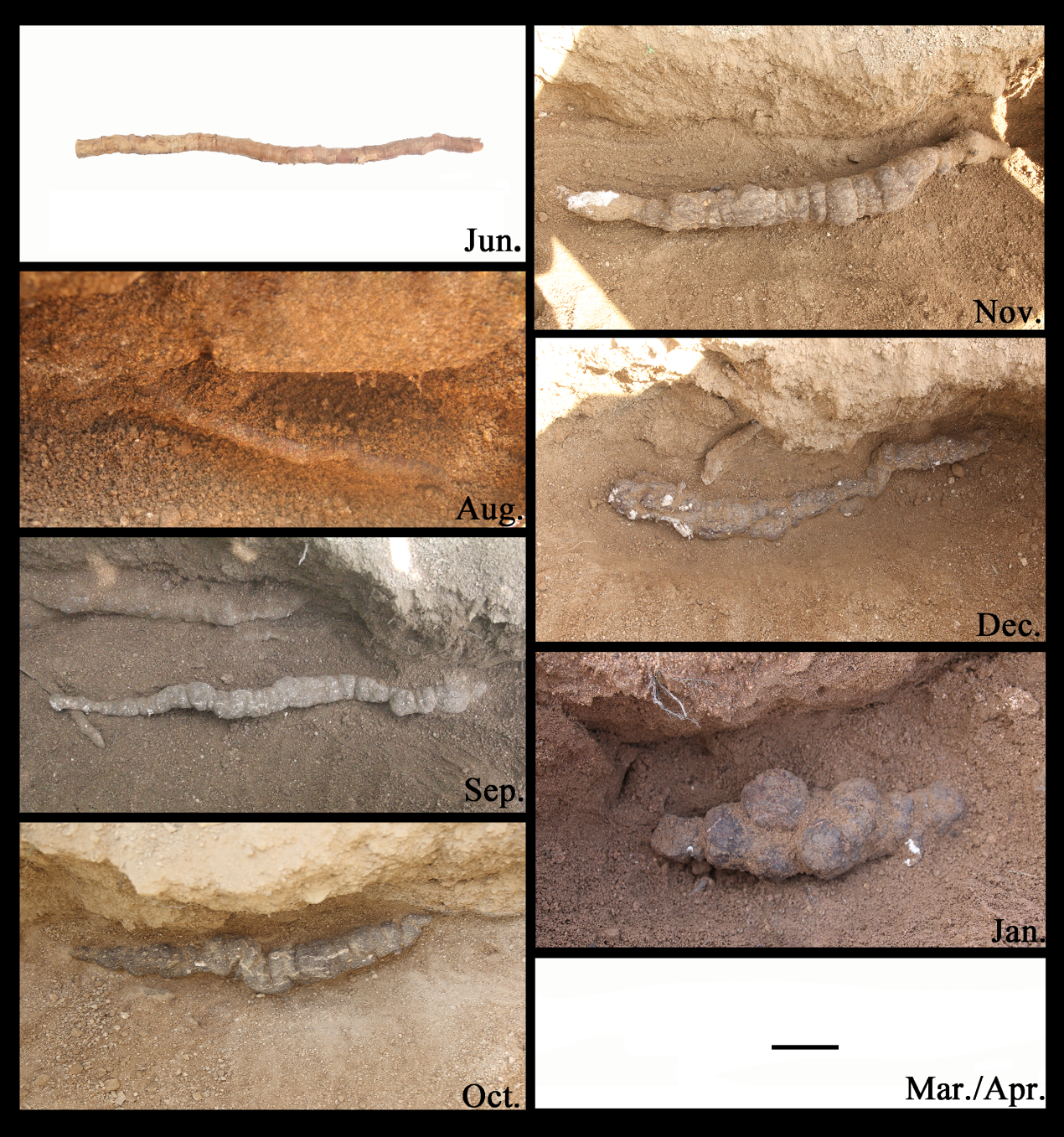


Supplementary Figure 1. Environment of field of Fushen from nine growth periods (-: no found).


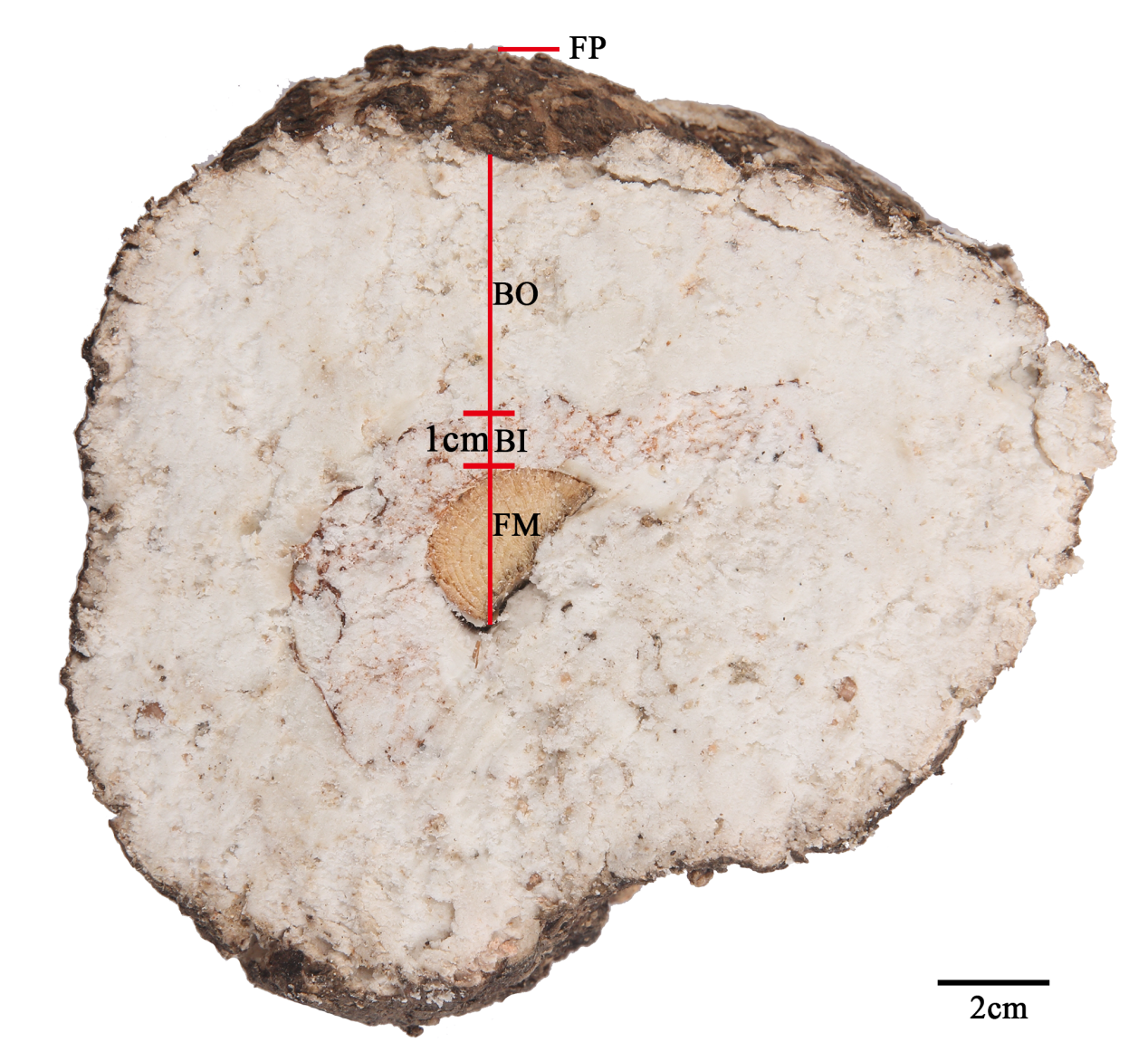


Supplementary Figure 2. Transverse section segmentation of four parts of Fushen.


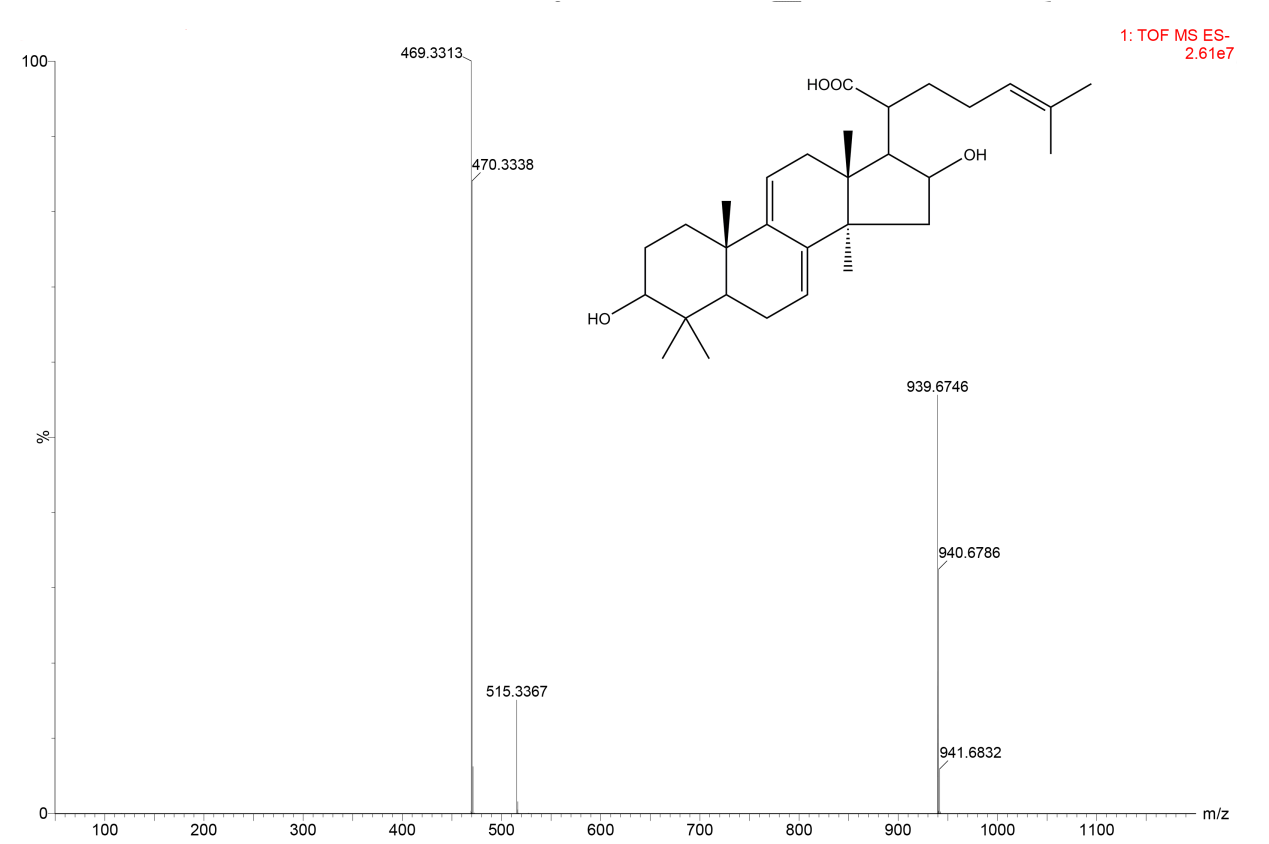


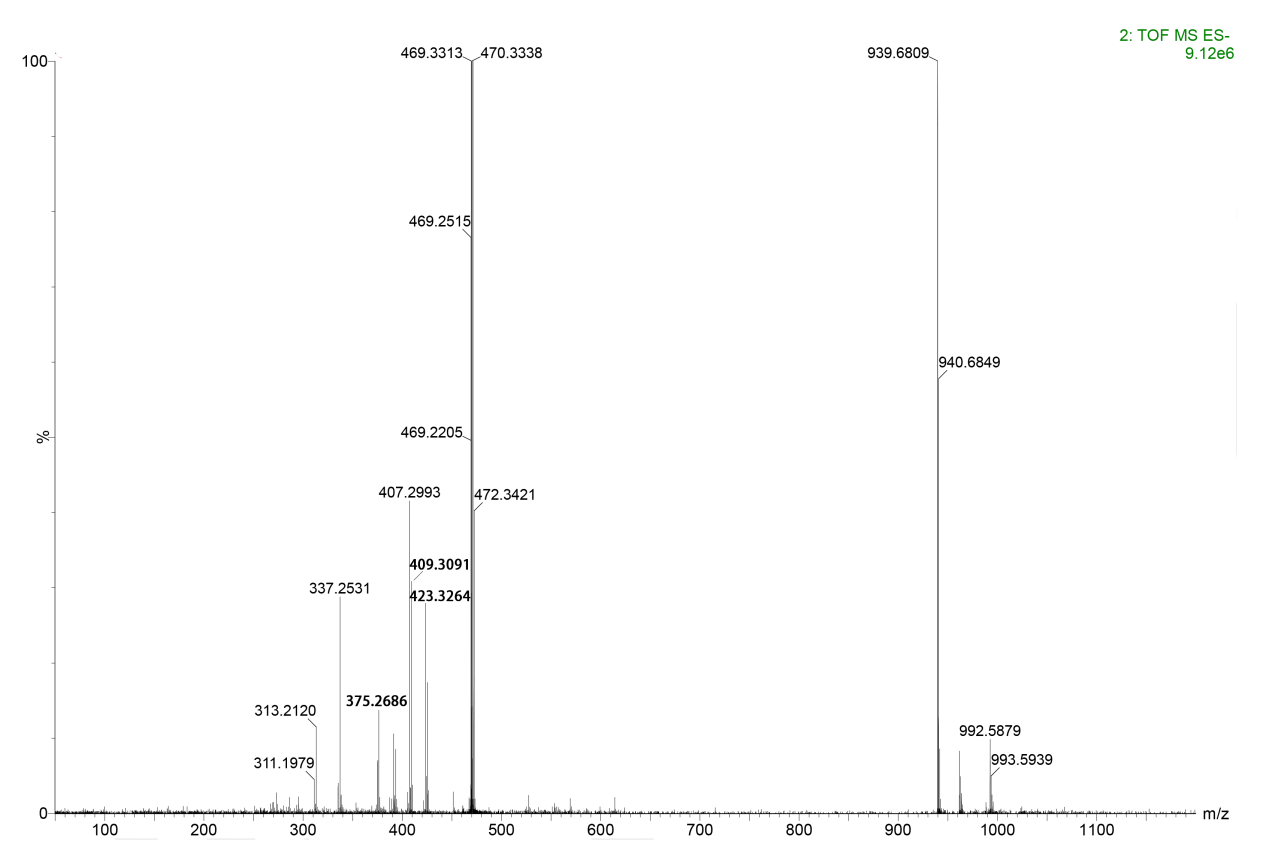


16α-Hydroxydehydrotrametenolic acid
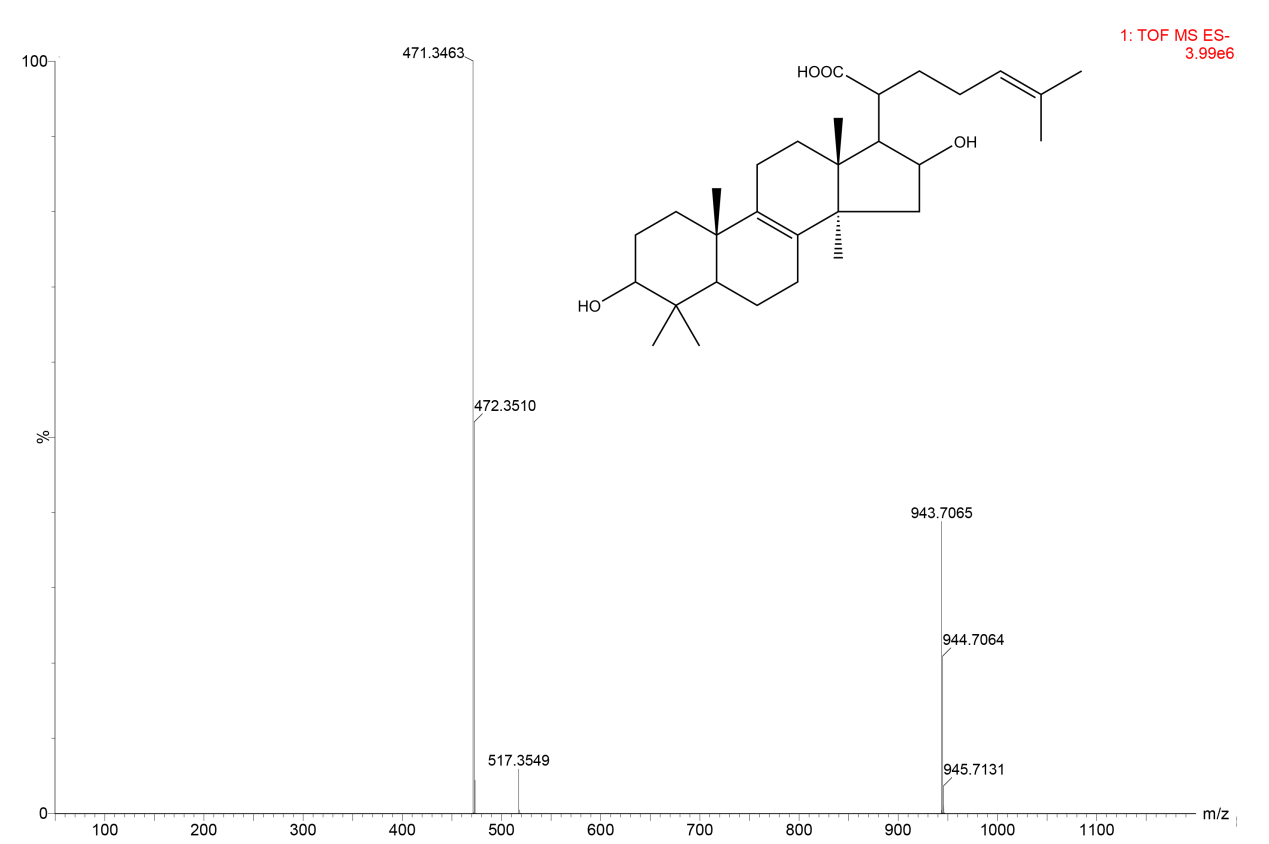

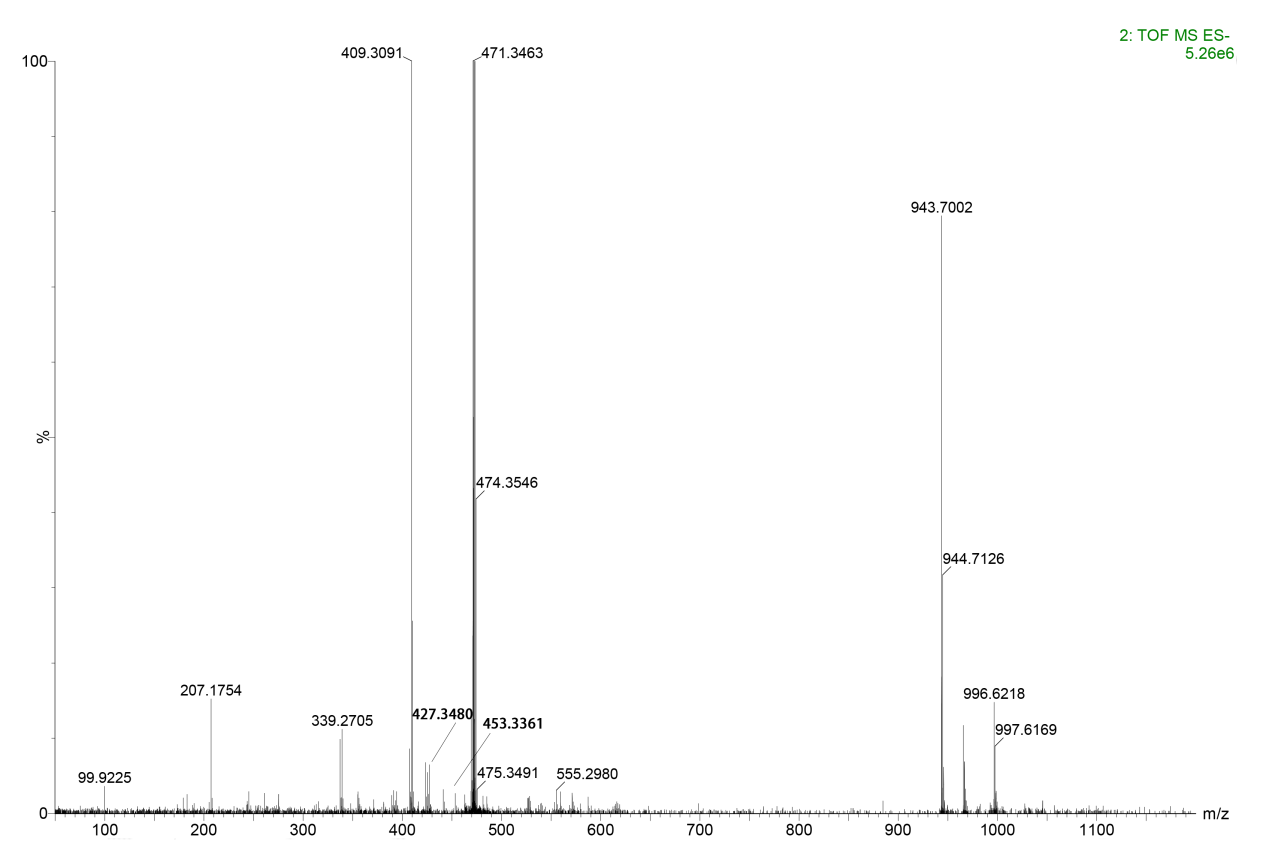


16α-Hydroxytrametenolic acid


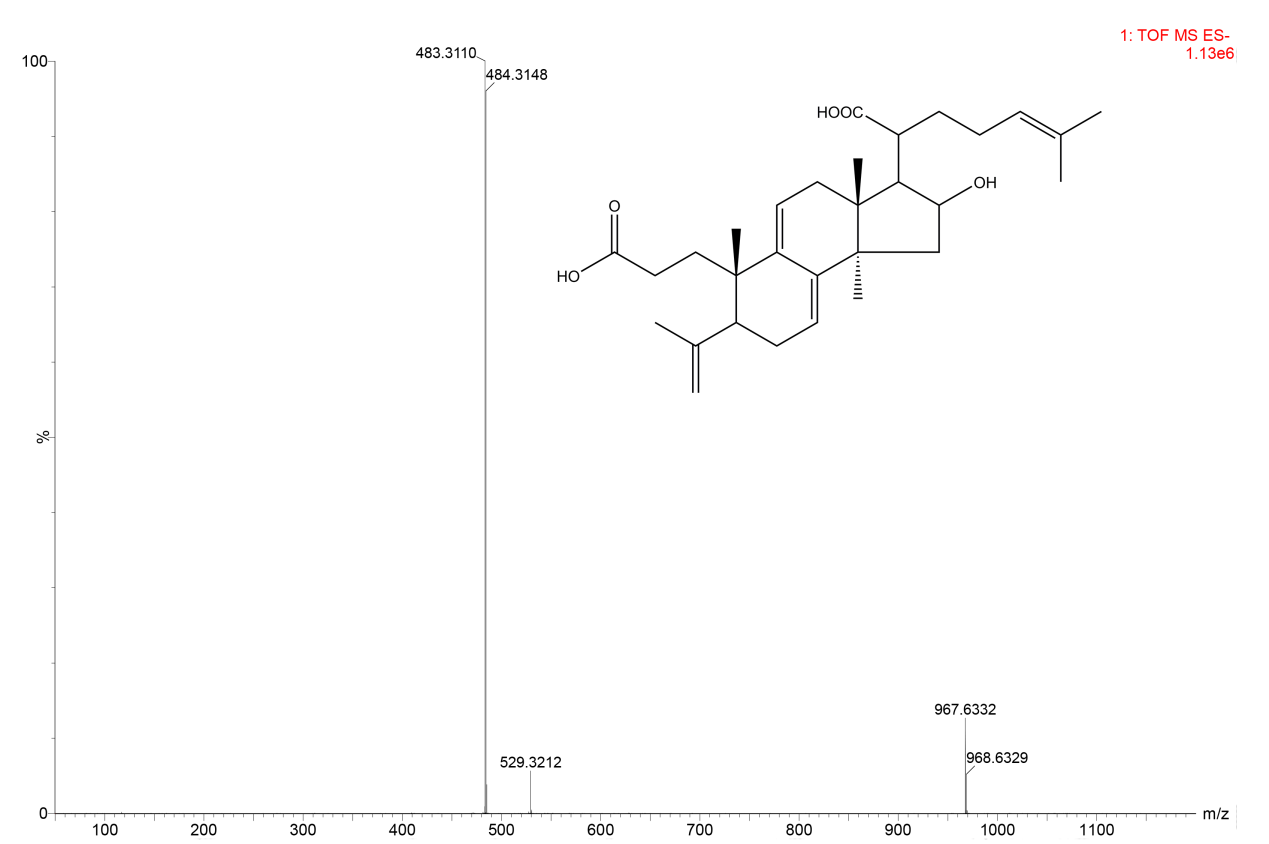

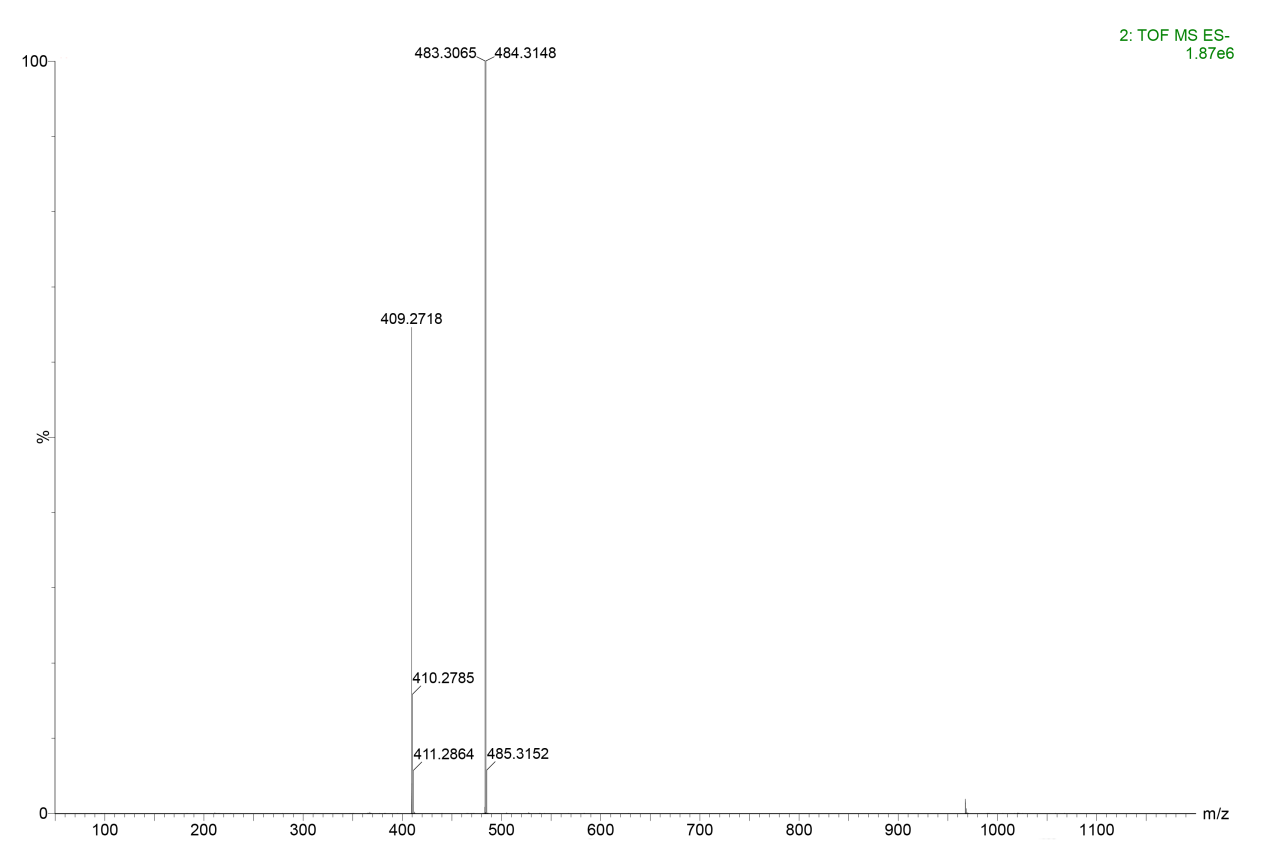


Poricoic acid B


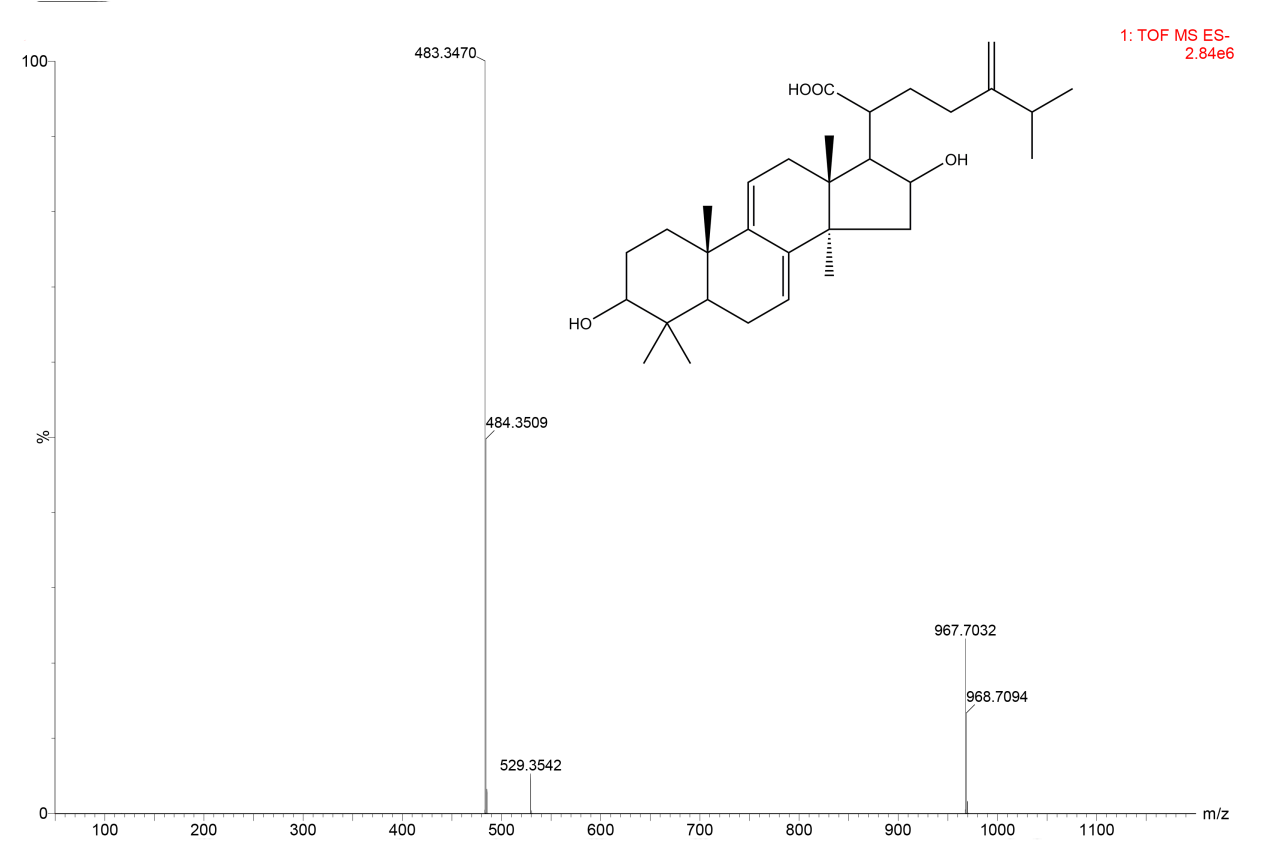

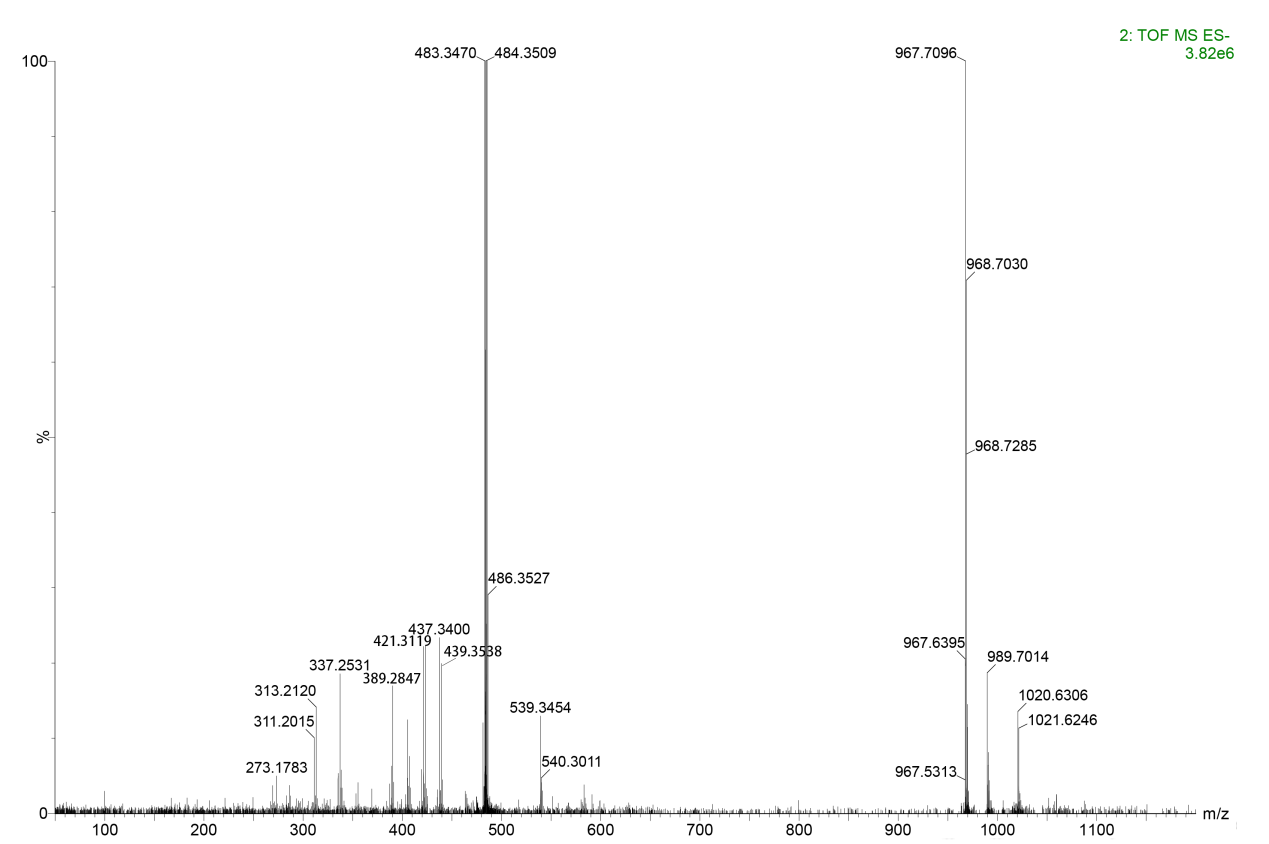
 Dehydrotumulosic acid


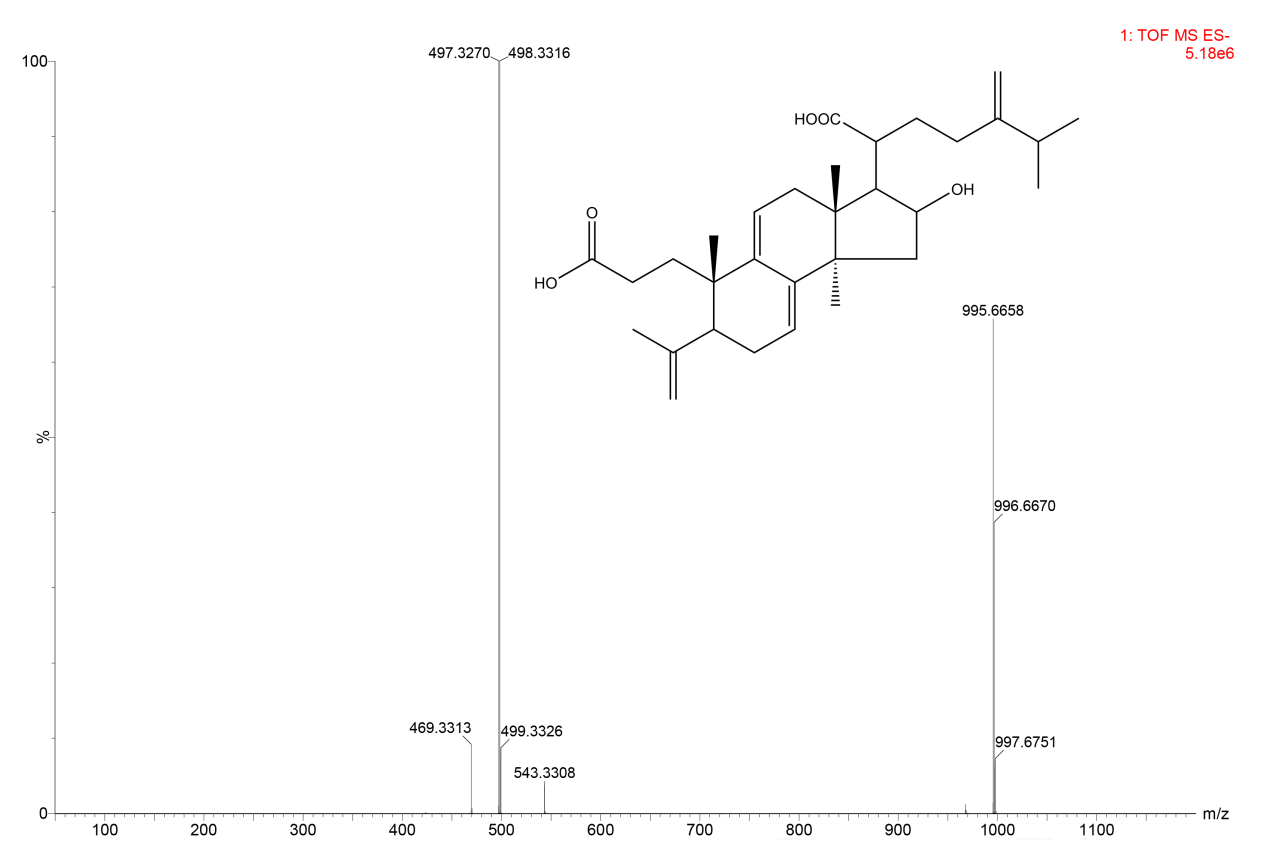

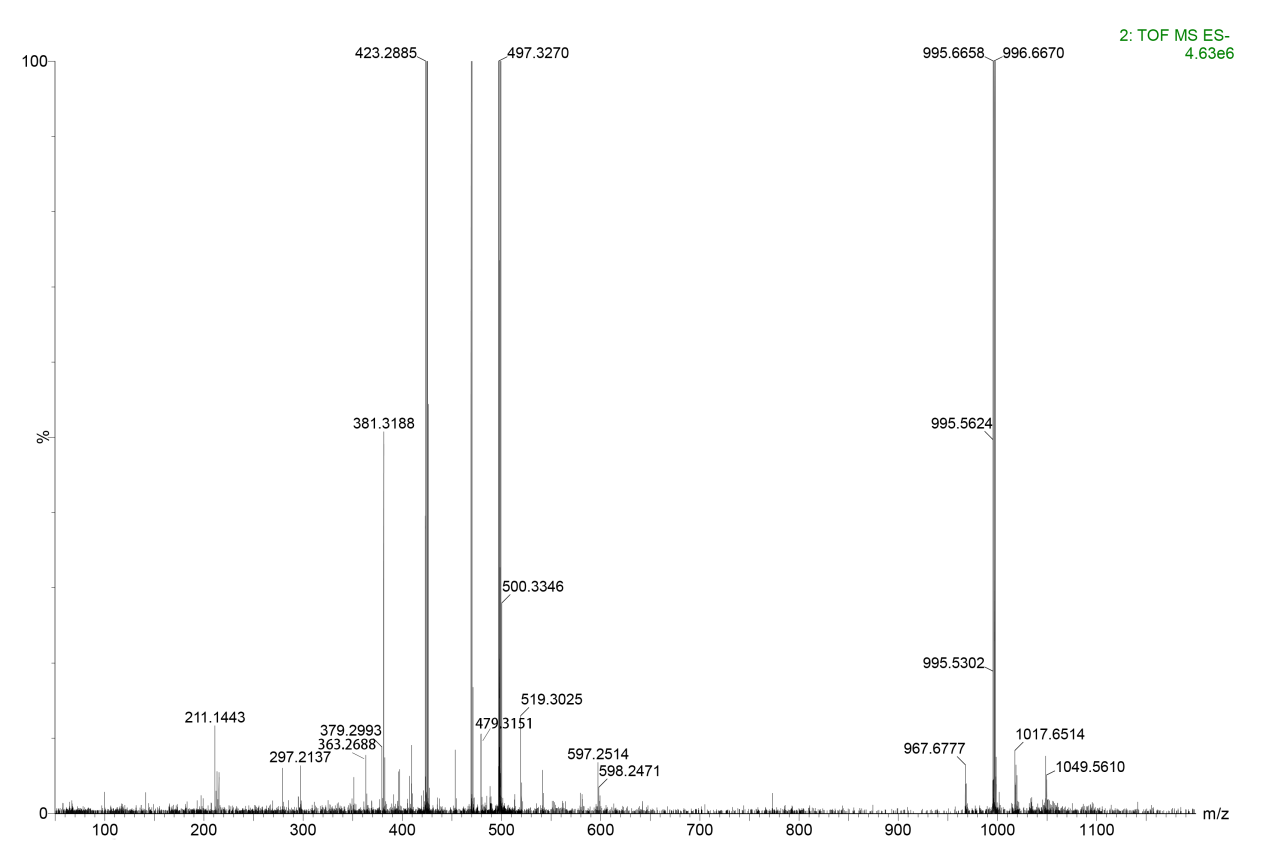
 Poricoic acid A


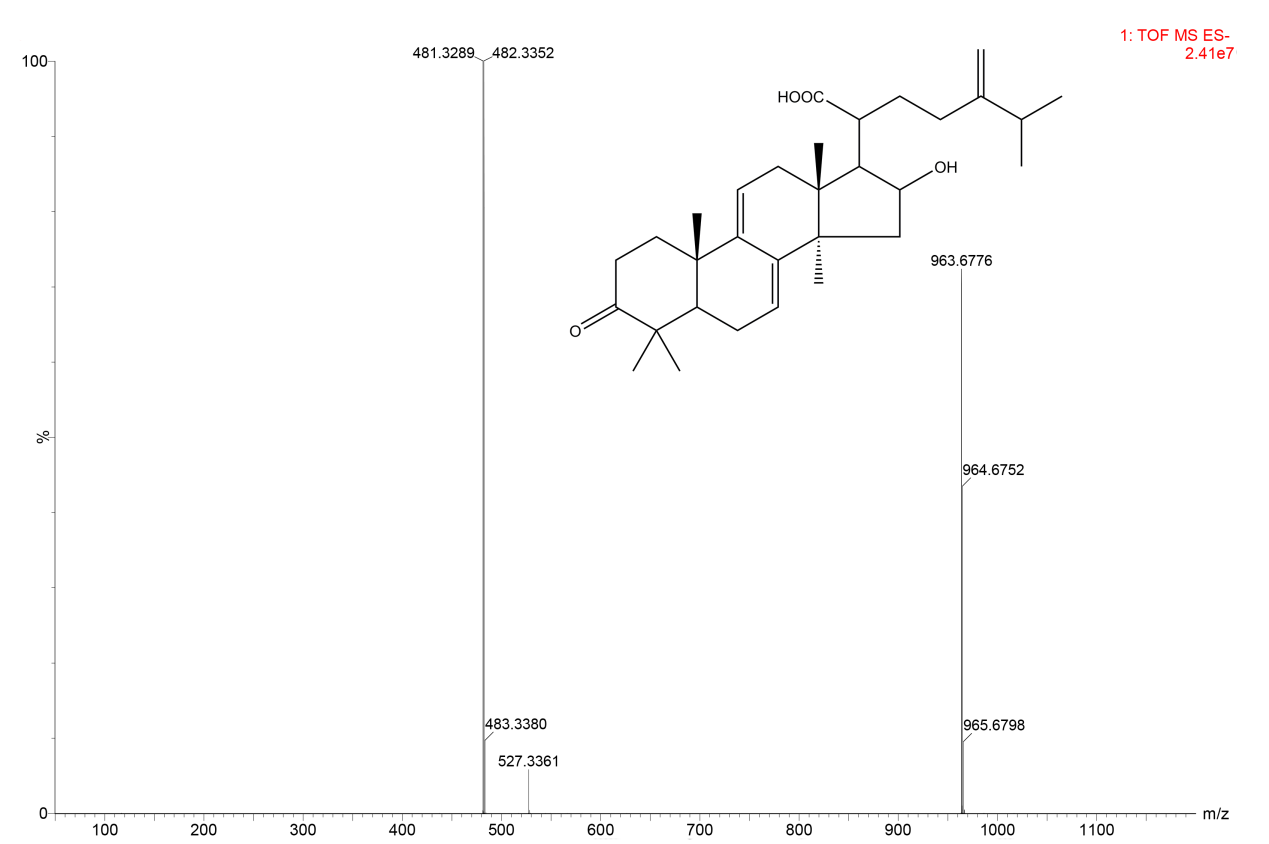

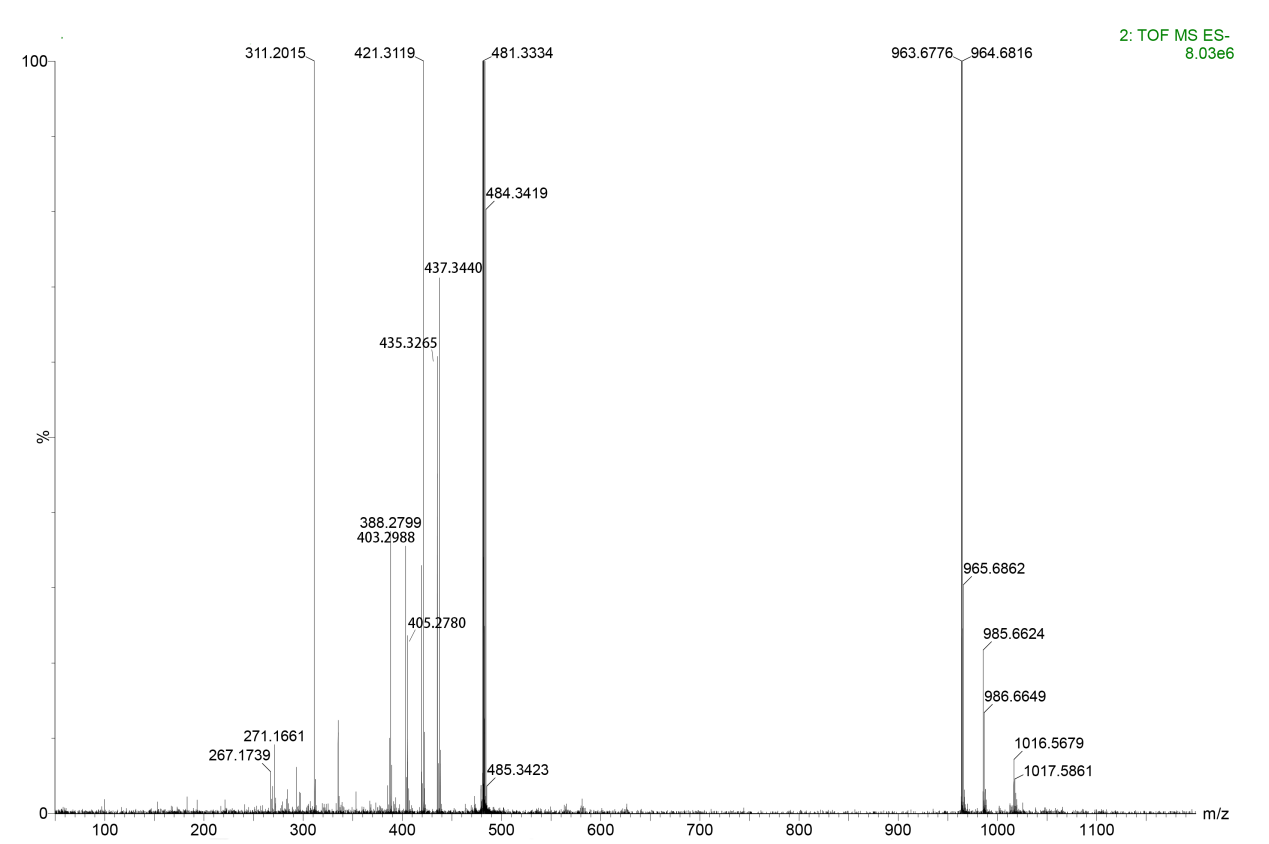
 Polyporenic acid C


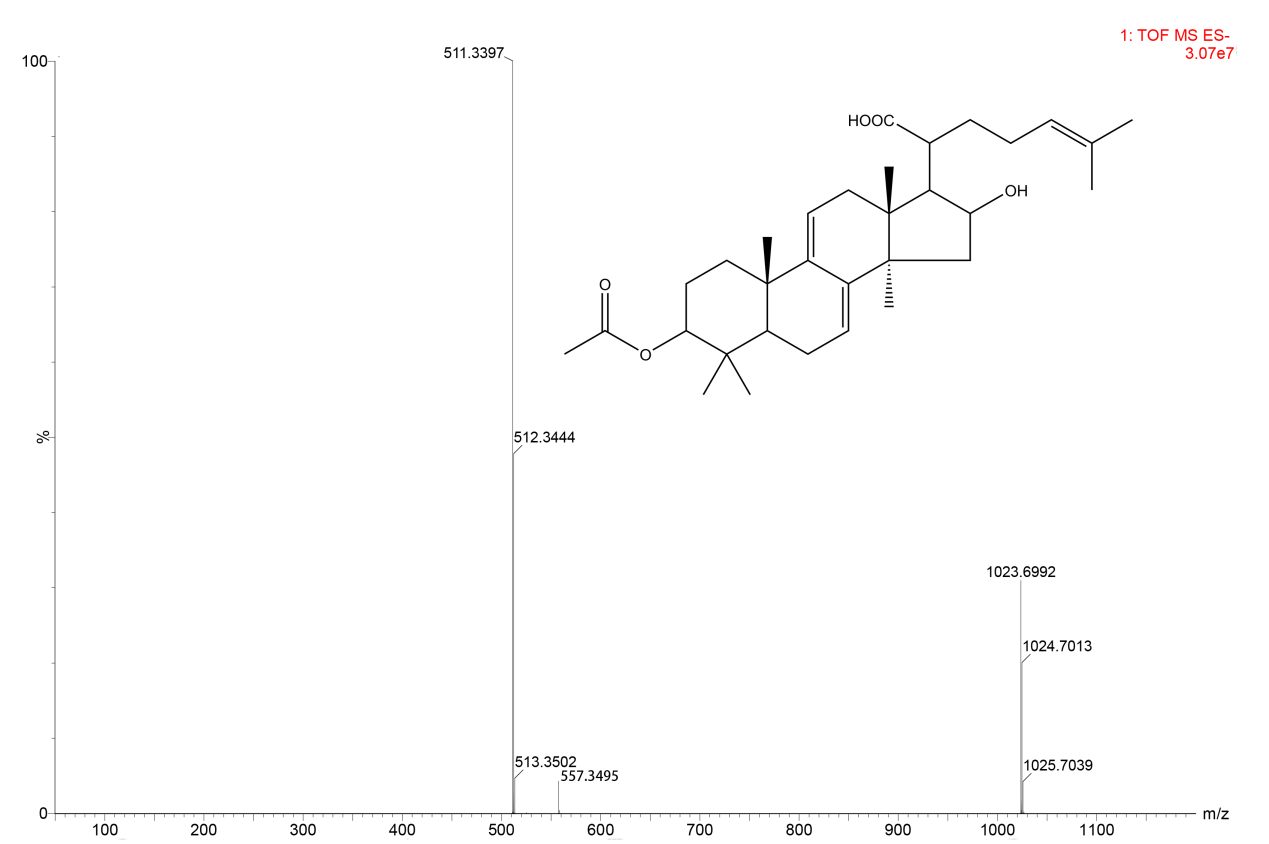

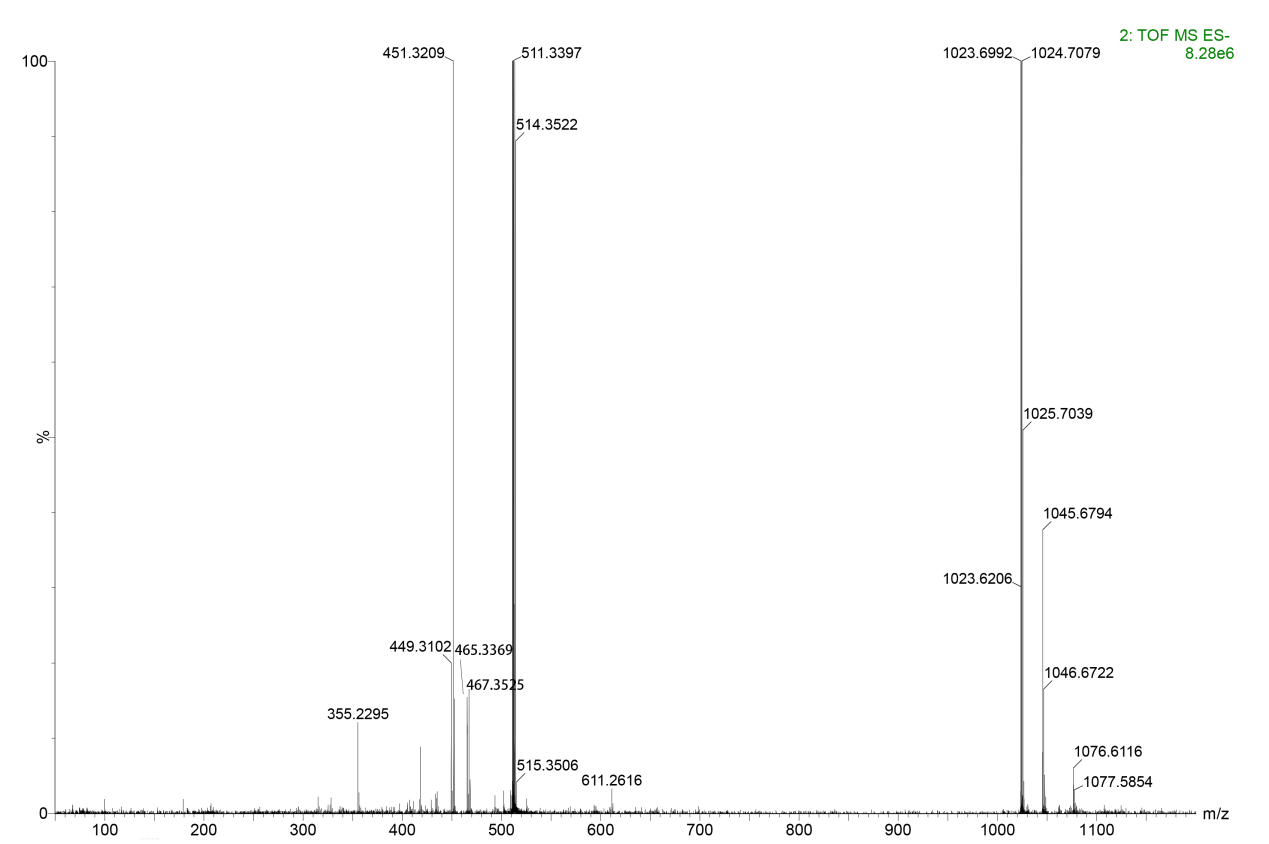
 3-O-Acetyl-16α-hydroxydehydrotrametenolic acid


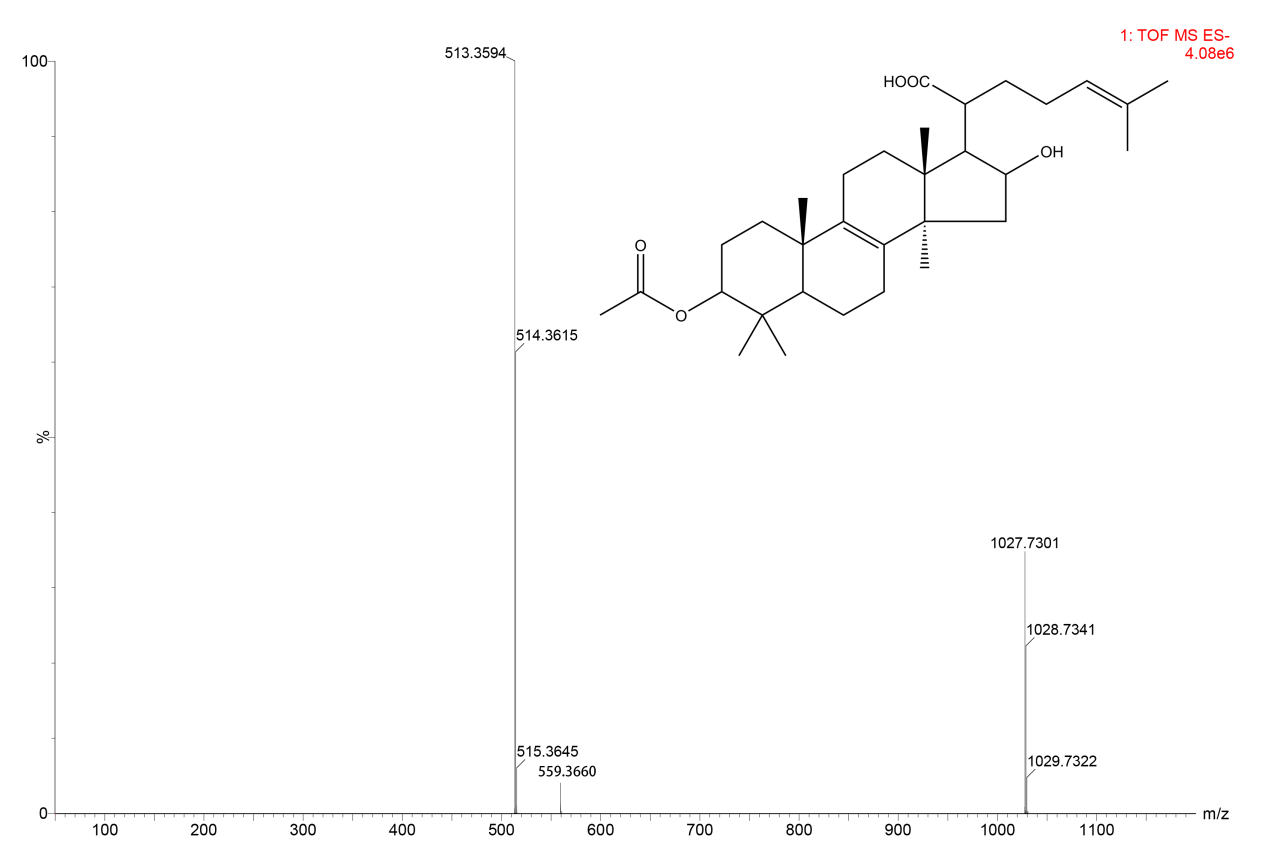

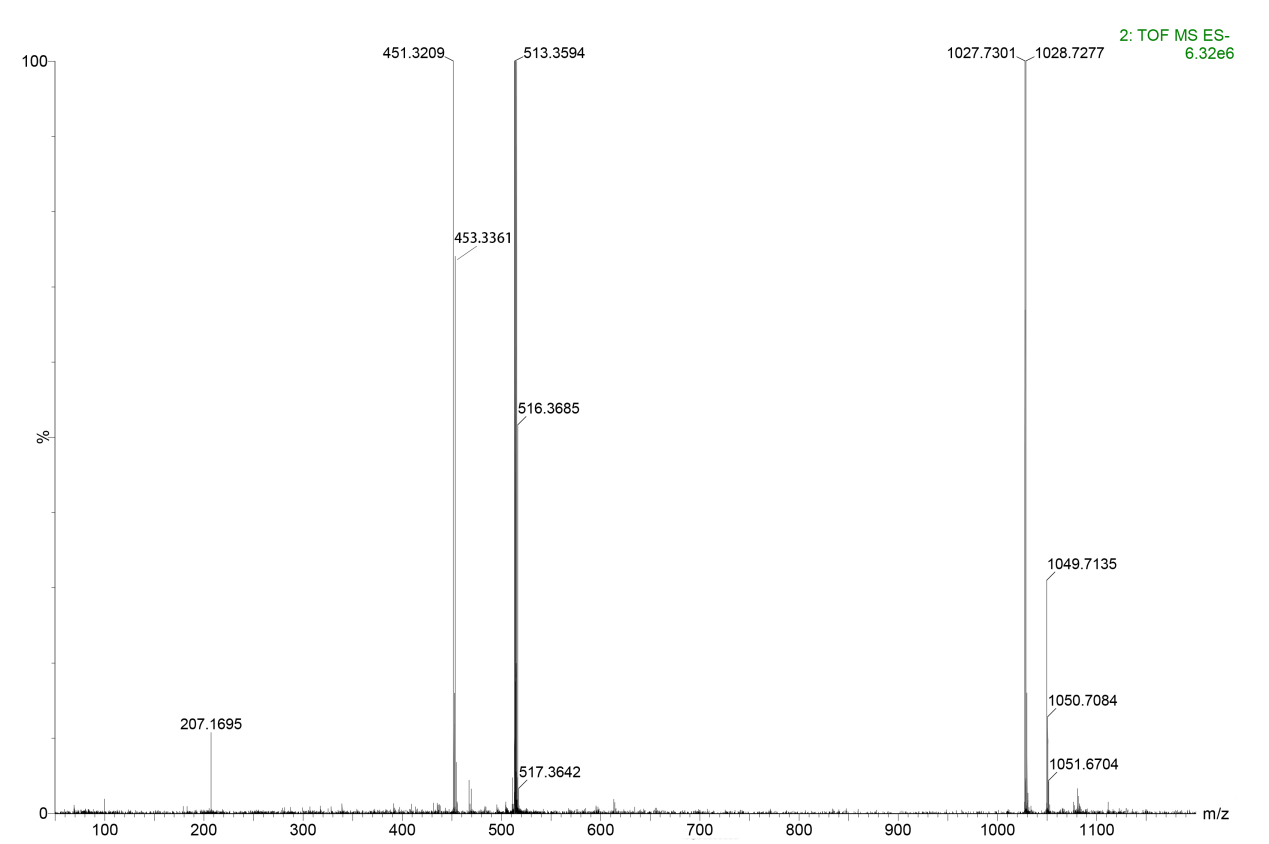
3-O-Acetyl-16α-hydroxytrametenolic acid


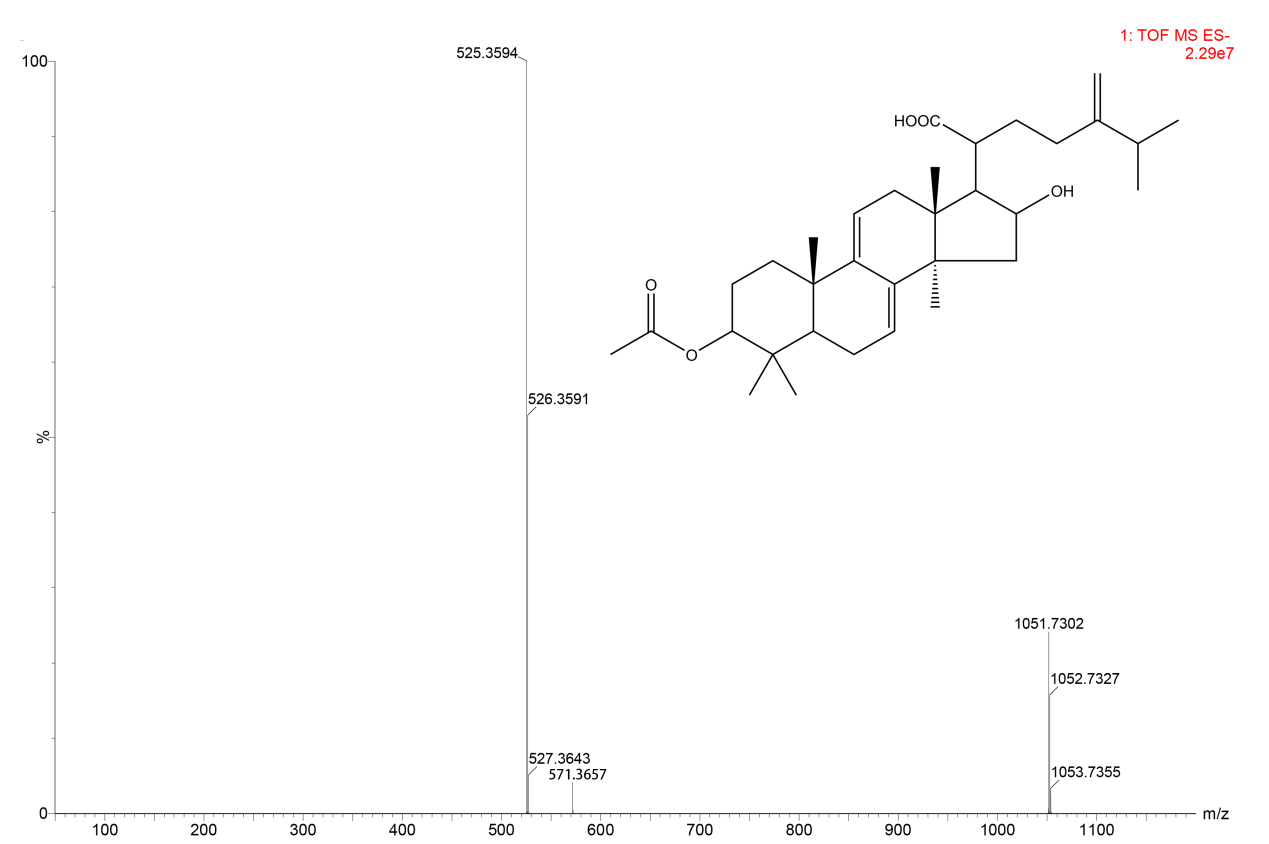

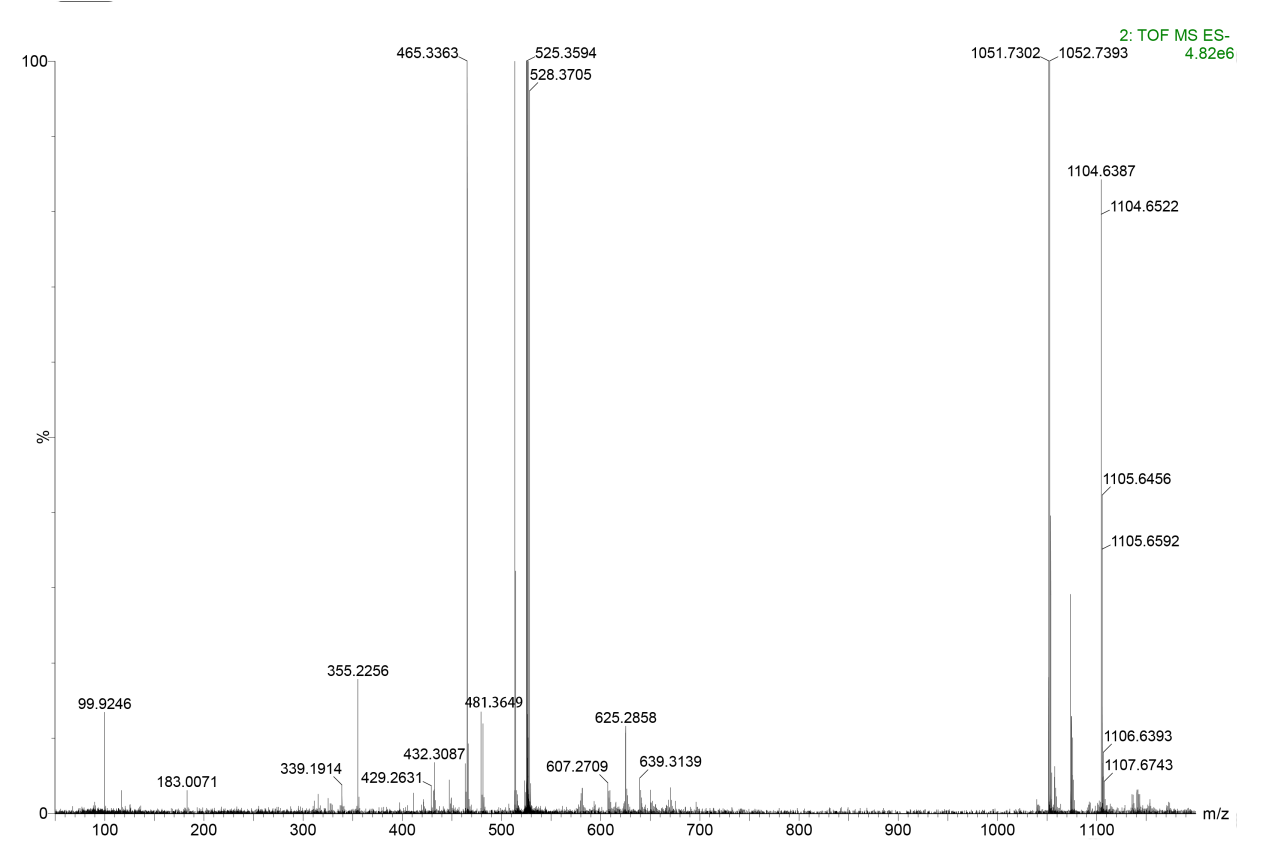
 Dehydropachymic acid


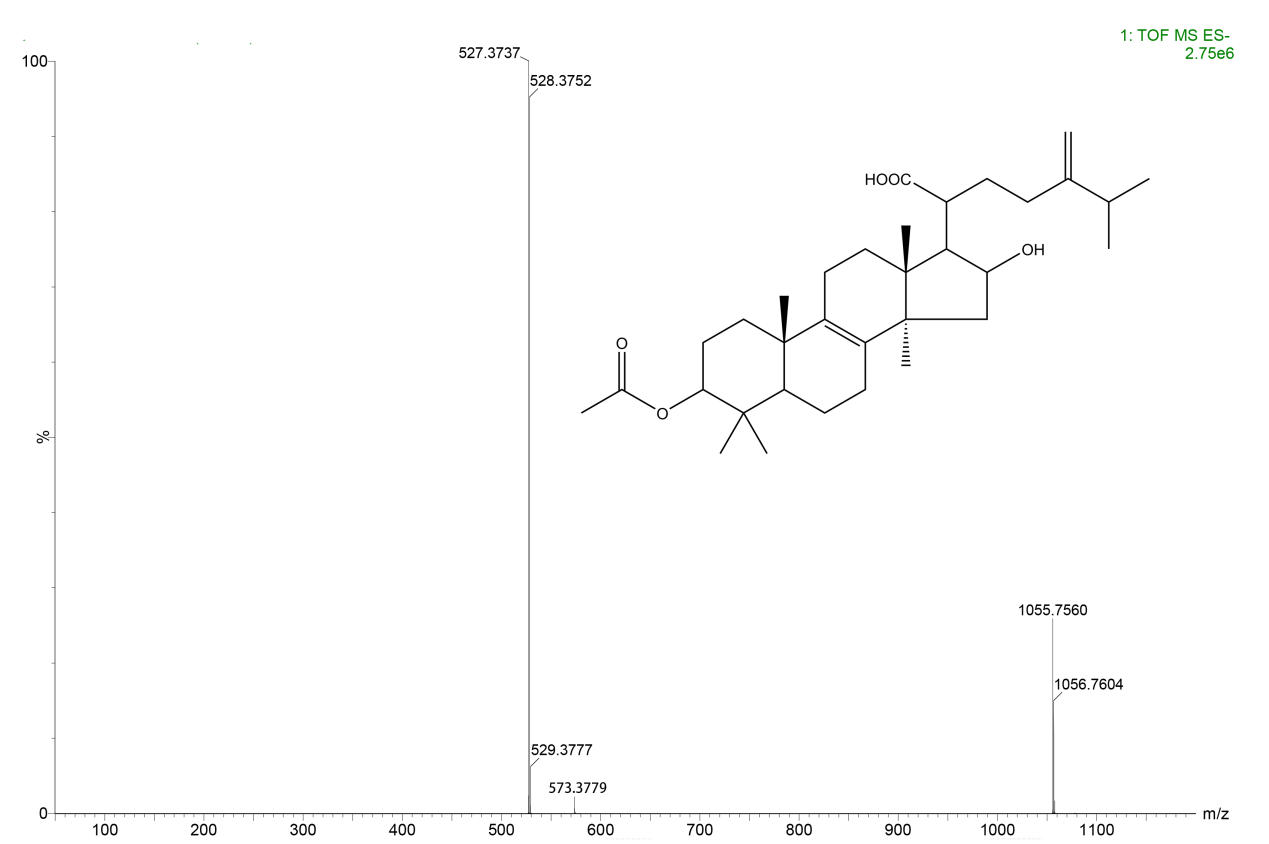

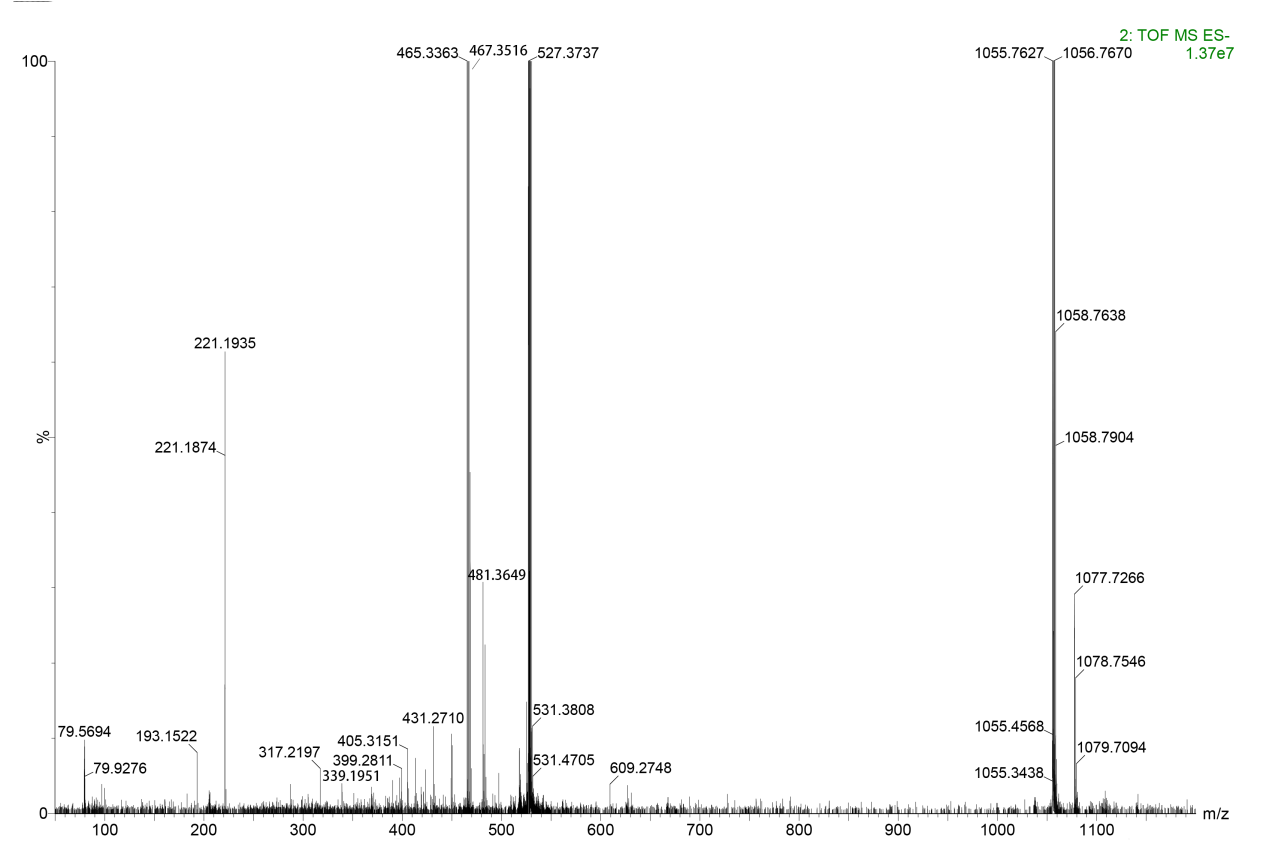
 Pachymic acid


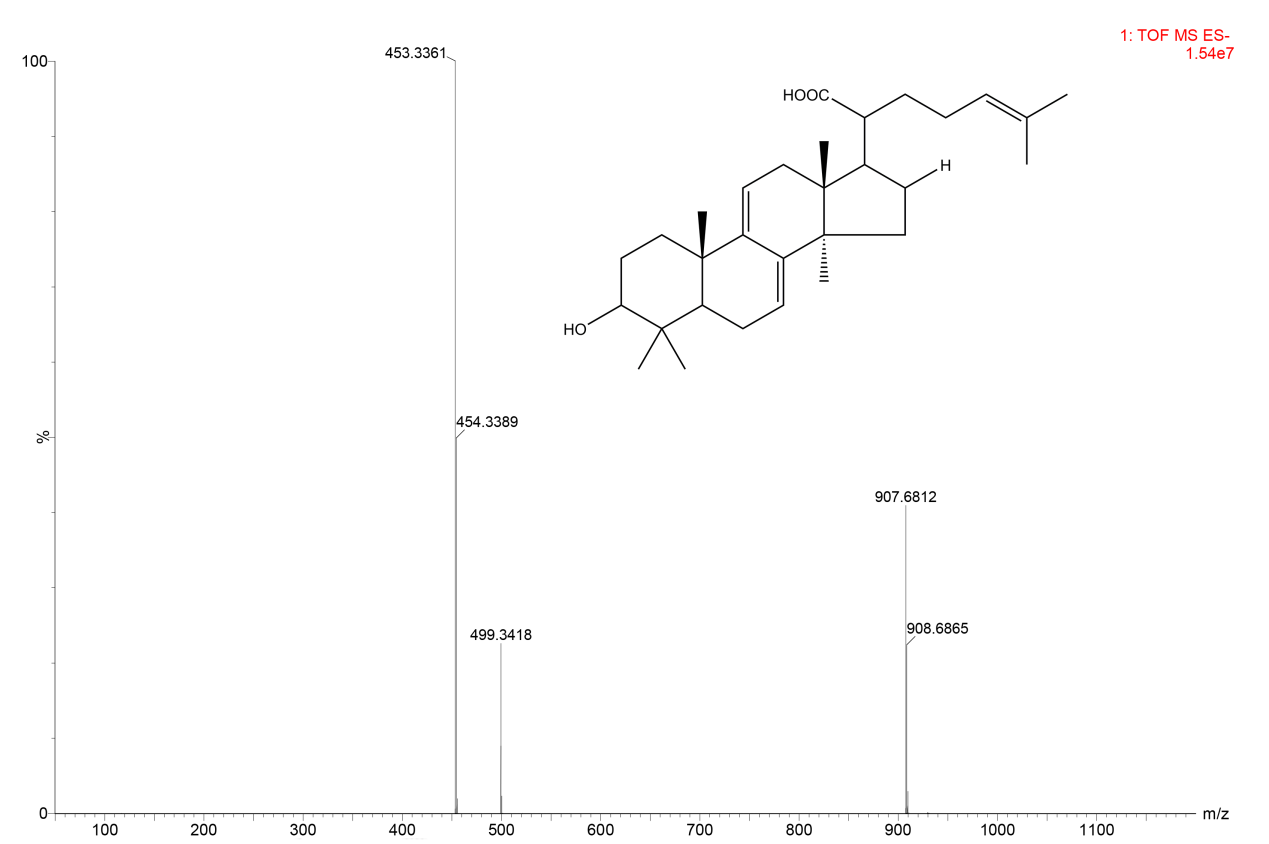

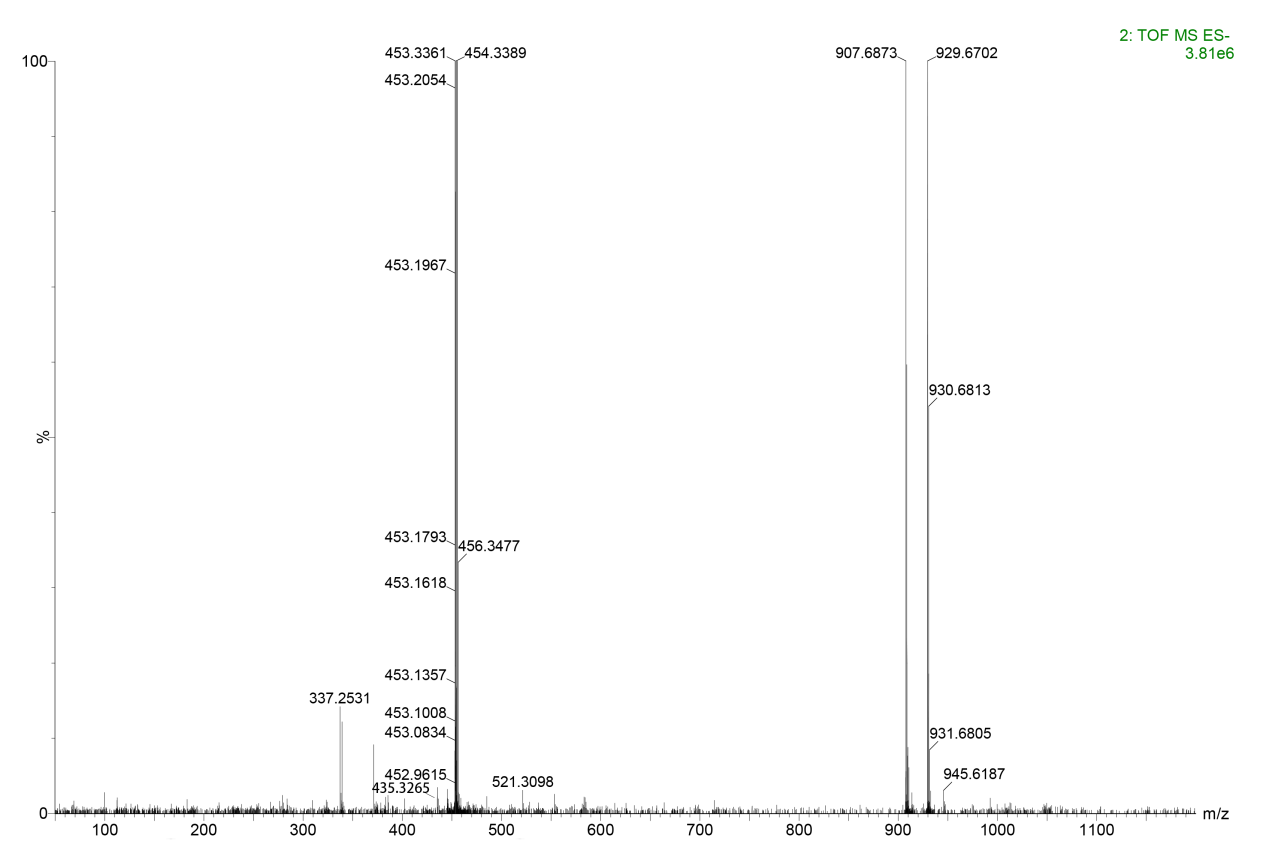
 Dehydrotrametenolic acid


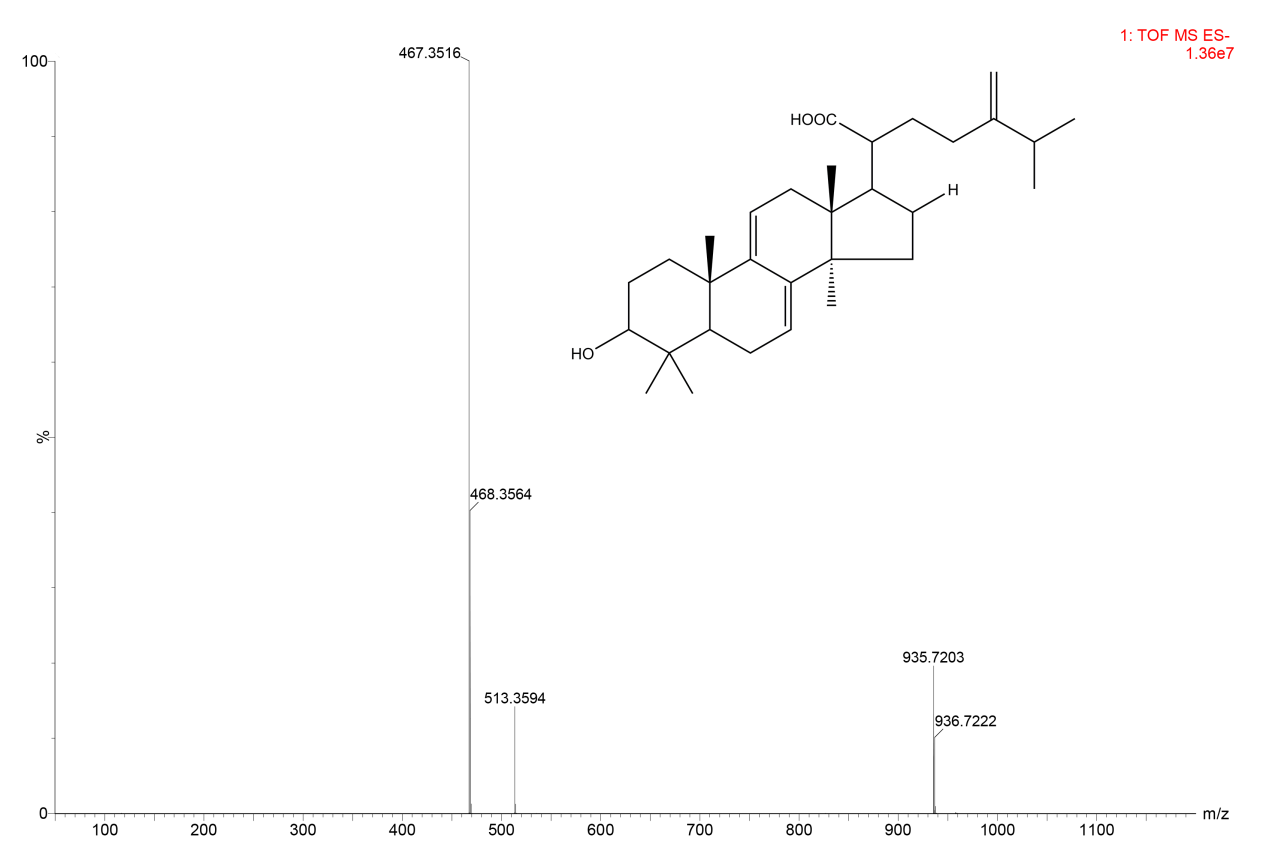

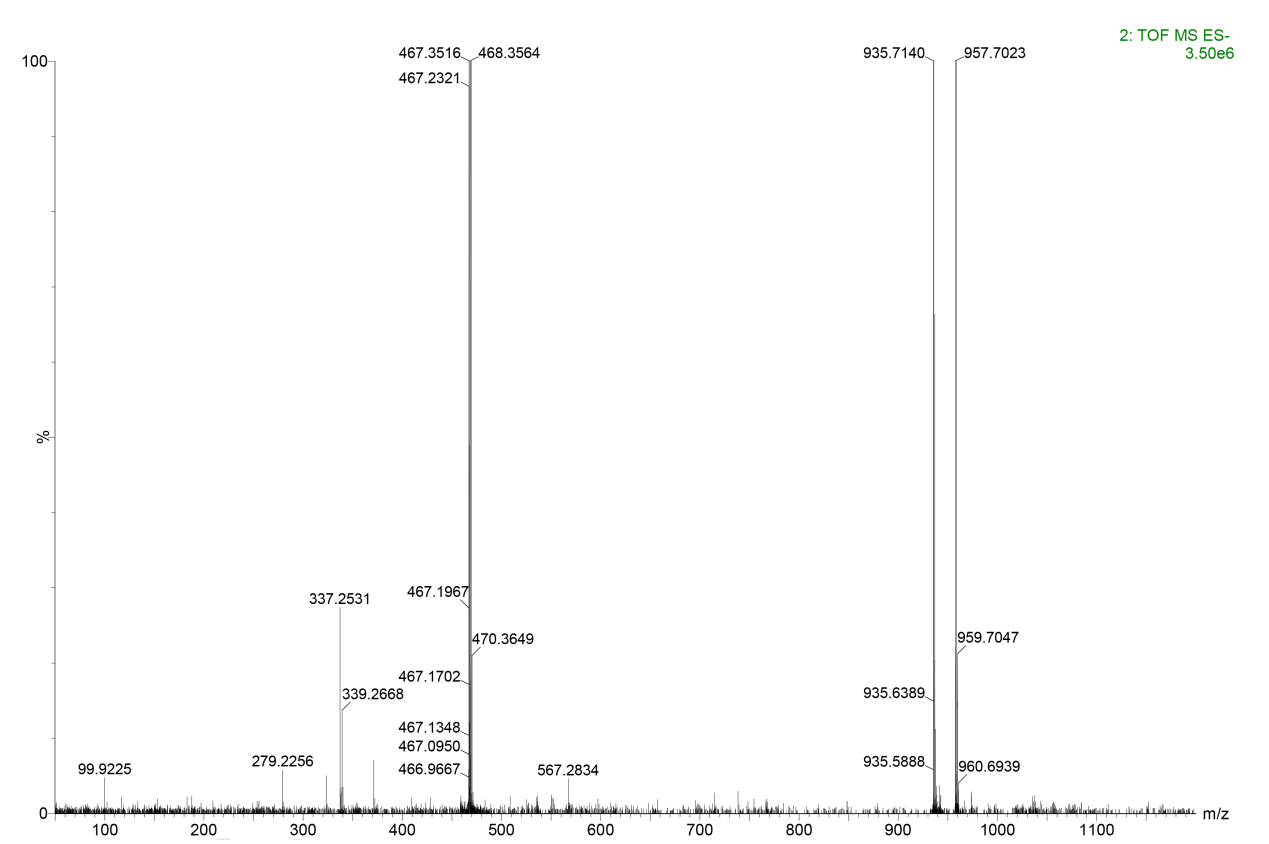
 Dehydroeburicoic acid


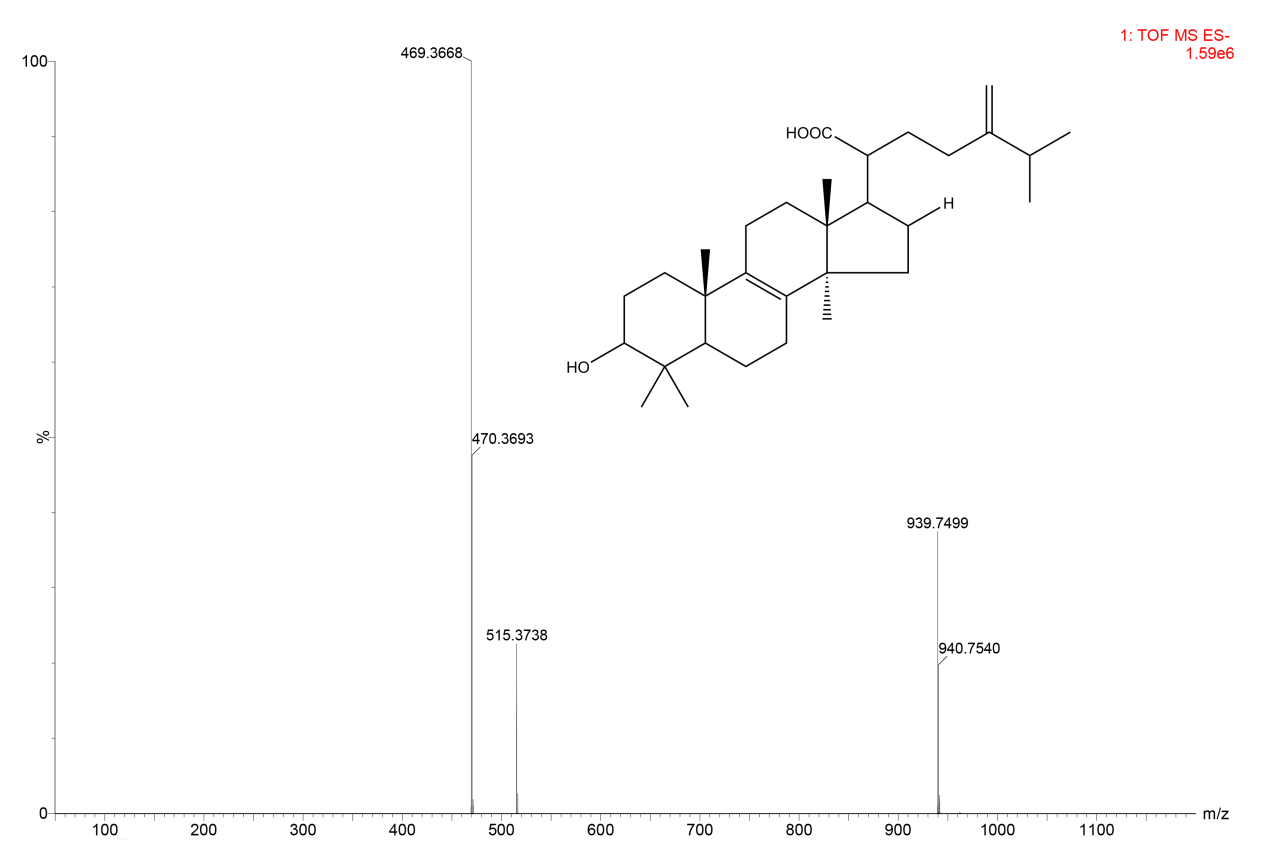

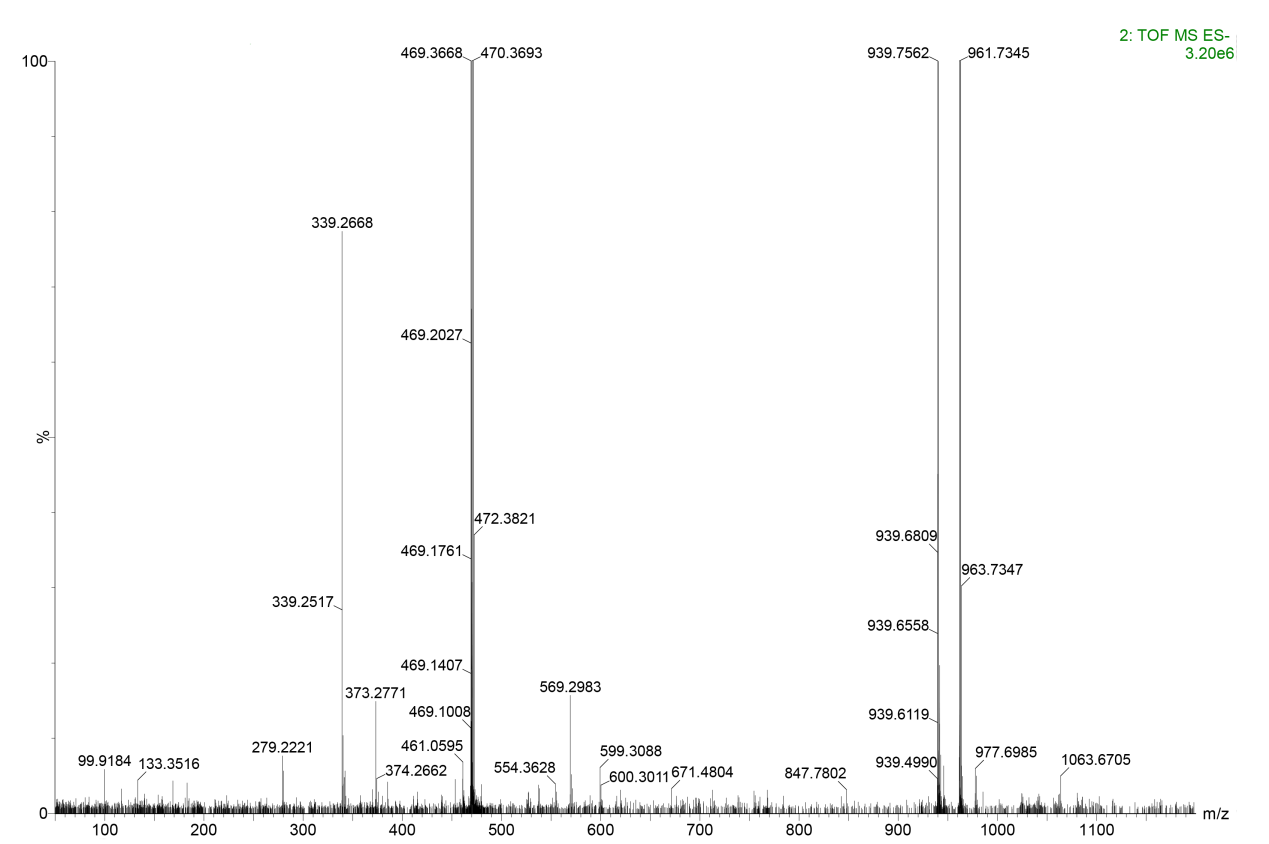
 Eburicoic acid

Supplementary Figure 3. The MS data and structures of the 13 reference compounds.


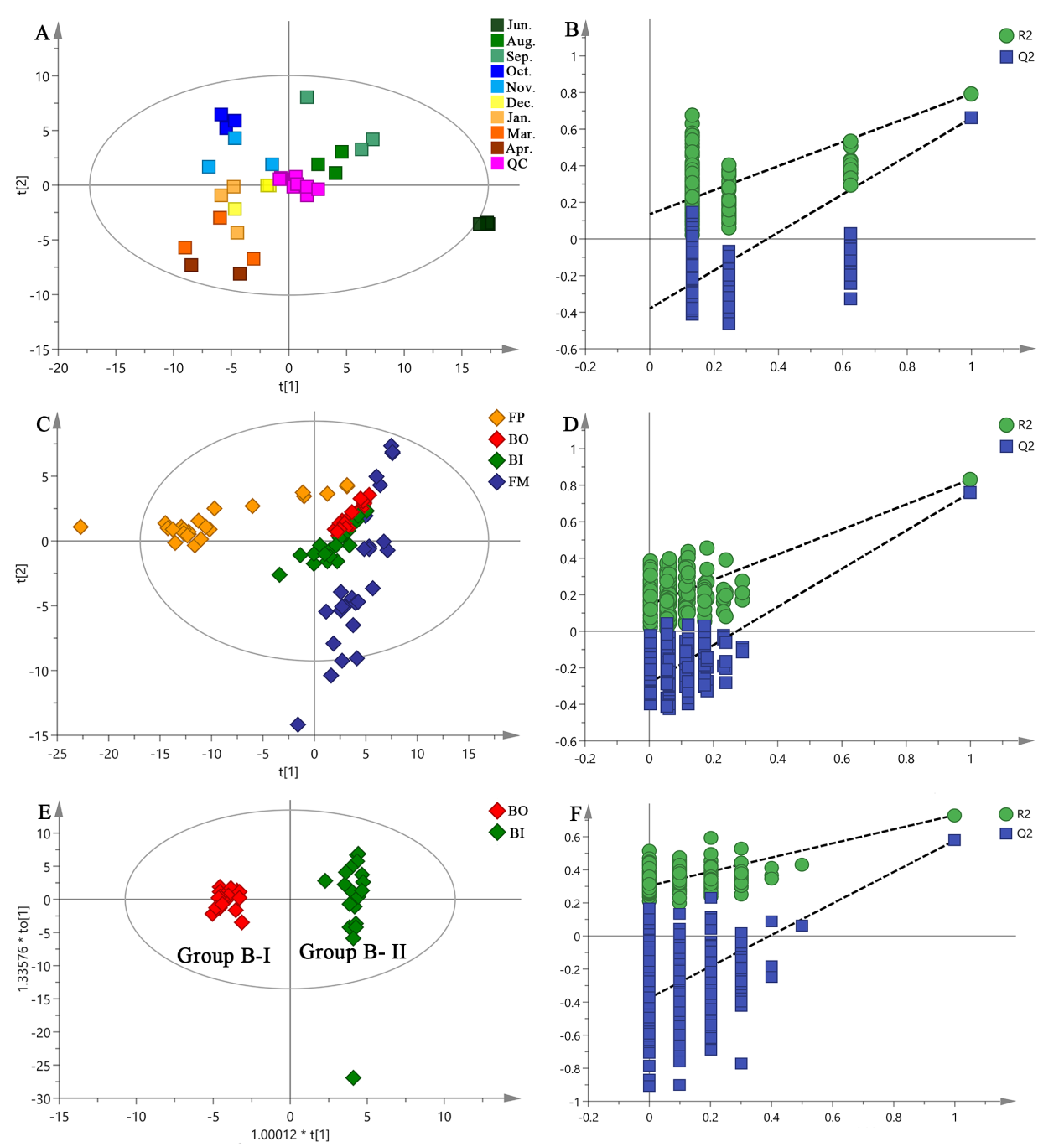


Supplementary Figure 4. PCA score plot of nine growth periods (A), permutation plot (after 200 times) of PLS-DA model of nine growth periods (B), PCA score plot of four parts (C), permutation plot (after 200 times) of PLS-DA model of four parts (D), OPLS-DA score plot of two parts (E), permutation plot (after 200 times) of OPLS-DA model of two parts (F) of Fushen based on UPLC-Q/TOF-MS in ESI^-^.


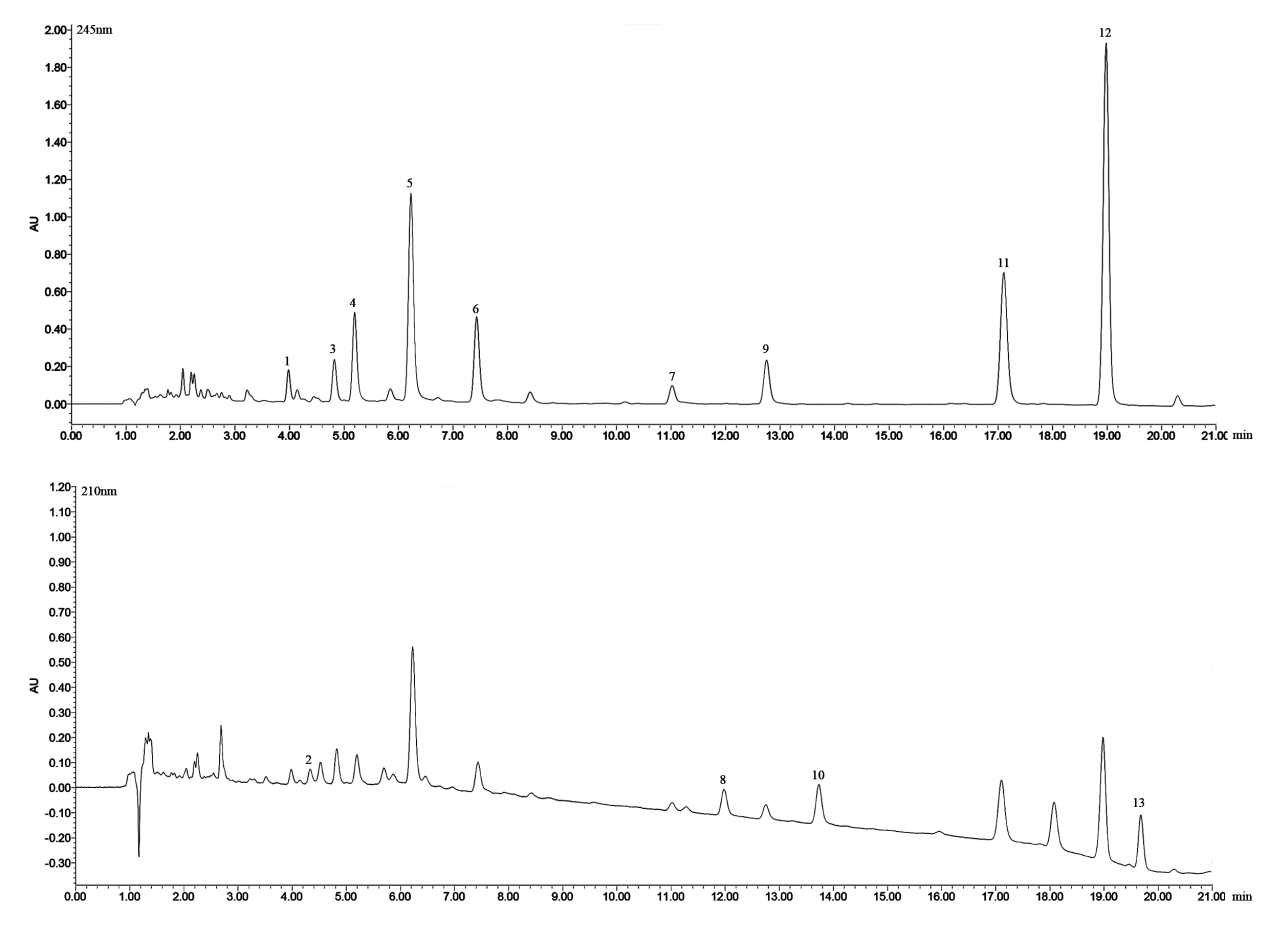
 Supplementary Figure 5. UPLC-PDA chromatogram of thirteen compounds in Fushen. (1) 16α-hydroxydehydrotrametenolic acid, (2) 16α-hydroxytrametenolic acid, (3) poricoic acid B, (4) dehydrotumulosic acid, (5) poricoic acid A, (6) polyporenic acid C, (7) 3-o-acetyl-16α-hydroxydehydrotrametenolic acid, (8) 3-o-acetyl-16α-hydroxytrametenolic acid, (9) dehydropachymic acid, (10) pachymic acid, (11) dehydrotratrametenolic acid, (12) dehydroeburicoic acid, (13) eburicoic acid.

Supplementary Table 1. Collection information of Fushen from nine growth periods.

| No | Sample No. | Color | Length/cm | Location | Collection Time |
| --- | --- | --- | --- | --- | --- |
| 1 | Jun.1 | brown | 46.7 | Jinzhai County, Anhui Province | 2019.6 |
| 2 | Jun.2 | brown | 50.1 | Jinzhai County, Anhui Province | 2019.6 |
| 3 | Jun.3 | brown | 52.0 | Jinzhai County, Anhui Province | 2019.6 |
| 4 | Aug.1 | brown | 58.3 | Jinzhai County, Anhui Province | 2019.8 |
| 5 | Aug.2 | brown | 60.7 | Jinzhai County, Anhui Province | 2019.8 |
| 6 | Aug.3 | brown | 59.0 | Jinzhai County, Anhui Province | 2019.8 |
| 7 | Sep.1 | dark brown | 77.2 | Jinzhai County, Anhui Province | 2019.9 |
| 8 | Sep.2 | dark brown | 73.0 | Jinzhai County, Anhui Province | 2019.9 |
| 9 | Sep.3 | dark brown | 70.9 | Jinzhai County, Anhui Province | 2019.9 |
| 10 | Oct.1 | dark brown | 82.5 | Jinzhai County, Anhui Province | 2019.10 |
| 11 | Oct.2 | dark brown | 78.4 | Jinzhai County, Anhui Province | 2019.10 |
| 12 | Oct.3 | dark brown | 79.1 | Jinzhai County, Anhui Province | 2019.10 |
| 13 | Nov.1 | dark brown | 77.5 | Jinzhai County, Anhui Province | 2019.11 |
| 14 | Nov.2 | dark brown | 80.2 | Jinzhai County, Anhui Province | 2019.11 |
| 15 | Nov.3 | dark brown | 79.0 | Jinzhai County, Anhui Province | 2019.11 |
| 16 | Dec.1 | dark brown | 75.0 | Jinzhai County, Anhui Province | 2019.12 |
| 17 | Dec.2 | dark brown | 76.7 | Jinzhai County, Anhui Province | 2019.12 |
| 18 | Dec.3 | dark brown | 75.3 | Jinzhai County, Anhui Province | 2019.12 |
| 19 | Jan.1 | dark brown | 78.7 | Jinzhai County, Anhui Province | 2020.1 |
| 20 | Jan.2 | dark brown | 80.2 | Jinzhai County, Anhui Province | 2020.1 |
| 21 | Jan.3 | dark brown | 74.5 | Jinzhai County, Anhui Province | 2020.1 |
| 22 | Mar.1 | dark brown | 82.0 | Jinzhai County, Anhui Province | 2020.3 |
| 23 | Mar.2 | dark brown | 82.3 | Jinzhai County, Anhui Province | 2020.3 |
| 24 | Mar.3 | dark brown | 87.5 | Jinzhai County, Anhui Province | 2020.3 |
| 25 | Apr.1 | dark brown | 81.6 | Jinzhai County, Anhui Province | 2020.4 |
| 26 | Apr.2 | dark brown | 75.4 | Jinzhai County, Anhui Province | 2020.4 |

Supplementary Table 2. Collection information from four parts of Fushen at nine growth periods.

| No | Batch No. | Color | Location | Collection Time |
| --- | --- | --- | --- | --- |
| 1 | Jun.FM1 | brown | Jinzhai County, Anhui Province | 2019.6 |
| 2 | Jun.FM2 | brown | Jinzhai County, Anhui Province | 2019.6 |
| 3 | Jun.FM3 | brown | Jinzhai County, Anhui Province | 2019.6 |
| 4 | Aug.FP1 | brown | Jinzhai County, Anhui Province | 2019.8 |
| 5 | Aug.FP2 | brown | Jinzhai County, Anhui Province | 2019.8 |
| 6 | Aug.FP3 | brown | Jinzhai County, Anhui Province | 2019.8 |
| 7 | Aug.FM1 | brown | Jinzhai County, Anhui Province | 2019.8 |
| 8 | Aug.FM2 | brown | Jinzhai County, Anhui Province | 2019.8 |
| 9 | Aug.FM3 | brown | Jinzhai County, Anhui Province | 2019.8 |
| 10 | Sep.FP1 | dark brown | Jinzhai County, Anhui Province | 2019.9 |
| 11 | Sep.FP2 | dark brown | Jinzhai County, Anhui Province | 2019.9 |
| 12 | Sep.FP3 | dark brown | Jinzhai County, Anhui Province | 2019.9 |
| 13 | Sep.BO1 | white | Jinzhai County, Anhui Province | 2019.9 |
| 14 | Sep.BO2 | white | Jinzhai County, Anhui Province | 2019.9 |
| 15 | Sep.BO3 | white | Jinzhai County, Anhui Province | 2019.9 |
| 16 | Sep.BI1 | white | Jinzhai County, Anhui Province | 2019.9 |
| 17 | Sep.BI2 | white | Jinzhai County, Anhui Province | 2019.9 |
| 18 | Sep.BI3 | white | Jinzhai County, Anhui Province | 2019.9 |
| 19 | Sep.FM1 | brown | Jinzhai County, Anhui Province | 2019.9 |
| 20 | Sep.FM2 | brown | Jinzhai County, Anhui Province | 2019.9 |
| 21 | Sep.FM3 | brown | Jinzhai County, Anhui Province | 2019.9 |
| 22 | Oct.FP1 | dark brown | Jinzhai County, Anhui Province | 2019.10 |
| 23 | Oct.FP2 | dark brown | Jinzhai County, Anhui Province | 2019.10 |
| 24 | Oct.FP3 | dark brown | Jinzhai County, Anhui Province | 2019.10 |
| 25 | Oct.BO1 | white | Jinzhai County, Anhui Province | 2019.10 |
| 26 | Oct.BO2 | white | Jinzhai County, Anhui Province | 2019.10 |
| 27 | Oct.BO3 | white | Jinzhai County, Anhui Province | 2019.10 |
| 28 | Oct.BI1 | white | Jinzhai County, Anhui Province | 2019.10 |
| 29 | Oct.BI2 | white | Jinzhai County, Anhui Province | 2019.10 |
| 30 | Oct.BI3 | white | Jinzhai County, Anhui Province | 2019.10 |
| 31 | Oct.FM1 | brown | Jinzhai County, Anhui Province | 2019.10 |
| 32 | Oct.FM2 | brown | Jinzhai County, Anhui Province | 2019.10 |
| 33 | Oct.FM3 | brown | Jinzhai County, Anhui Province | 2019.10 |
| 34 | Nov.FP1 | dark brown | Jinzhai County, Anhui Province | 2019.11 |
| 35 | Nov.FP2 | dark brown | Jinzhai County, Anhui Province | 2019.11 |
| 36 | Nov.FP3 | dark brown | Jinzhai County, Anhui Province | 2019.11 |
| 37 | Nov.BO1 | white | Jinzhai County, Anhui Province | 2019.11 |
| 38 | Nov.BO2 | white | Jinzhai County, Anhui Province | 2019.11 |
| 39 | Nov.BO3 | white | Jinzhai County, Anhui Province | 2019.11 |
| 40 | Nov.BI1 | white | Jinzhai County, Anhui Province | 2019.11 |
| 41 | Nov.BI2 | white | Jinzhai County, Anhui Province | 2019.11 |
| 42 | Nov.BI3 | white | Jinzhai County, Anhui Province | 2019.11 |
| 43 | Nov.FM1 | brown | Jinzhai County, Anhui Province | 2019.11 |
| 44 | Nov.FM2 | brown | Jinzhai County, Anhui Province | 2019.11 |
| 45 | Nov.FM3 | brown | Jinzhai County, Anhui Province | 2019.11 |
| 46 | Dec.FP1 | dark brown | Jinzhai County, Anhui Province | 2019.12 |
| 47 | Dec.FP2 | dark brown | Jinzhai County, Anhui Province | 2019.12 |
| 48 | Dec.FP3 | dark brown | Jinzhai County, Anhui Province | 2019.12 |
| 49 | Dec.BO1 | white | Jinzhai County, Anhui Province | 2019.12 |
| 50 | Dec.BO2 | white | Jinzhai County, Anhui Province | 2019.12 |
| 51 | Dec.BO3 | white | Jinzhai County, Anhui Province | 2019.12 |
| 52 | Dec.BI1 | white | Jinzhai County, Anhui Province | 2019.12 |
| 53 | Dec.BI2 | white | Jinzhai County, Anhui Province | 2019.12 |
| 54 | Dec.BI3 | white | Jinzhai County, Anhui Province | 2019.12 |
| 55 | Dec.FM1 | brown | Jinzhai County, Anhui Province | 2019.12 |
| 56 | Dec.FM2 | brown | Jinzhai County, Anhui Province | 2019.12 |
| 57 | Dec.FM3 | brown | Jinzhai County, Anhui Province | 2019.12 |
| 58 | Jan.FP1 | dark brown | Jinzhai County, Anhui Province | 2020.1 |
| 59 | Jan.FP2 | dark brown | Jinzhai County, Anhui Province | 2020.1 |
| 60 | Jan.FP3 | dark brown | Jinzhai County, Anhui Province | 2020.1 |
| 61 | Jan.BO1 | white | Jinzhai County, Anhui Province | 2020.1 |
| 62 | Jan.BO2 | white | Jinzhai County, Anhui Province | 2020.1 |
| 63 | Jan.BO3 | white | Jinzhai County, Anhui Province | 2020.1 |
| 64 | Jan.BI1 | white | Jinzhai County, Anhui Province | 2020.1 |
| 65 | Jan.BI2 | white | Jinzhai County, Anhui Province | 2020.1 |
| 66 | Jan.BI3 | white | Jinzhai County, Anhui Province | 2020.1 |
| 67 | Jan.FM1 | brown | Jinzhai County, Anhui Province | 2020.1 |
| 68 | Jan.FM2 | brown | Jinzhai County, Anhui Province | 2020.1 |
| 69 | Jan.FM3 | brown | Jinzhai County, Anhui Province | 2020.1 |
| 70 | Mar.FP1 | dark brown | Jinzhai County, Anhui Province | 2020.3 |
| 71 | Mar.FP2 | dark brown | Jinzhai County, Anhui Province | 2020.3 |
| 72 | Mar.FP3 | dark brown | Jinzhai County, Anhui Province | 2020.3 |
| 73 | Mar.BO1 | white | Jinzhai County, Anhui Province | 2020.3 |
| 74 | Mar.BO2 | white | Jinzhai County, Anhui Province | 2020.3 |
| 75 | Mar.BO3 | white | Jinzhai County, Anhui Province | 2020.3 |
| 76 | Mar.BI1 | white | Jinzhai County, Anhui Province | 2020.3 |
| 77 | Mar.BI2 | white | Jinzhai County, Anhui Province | 2020.3 |
| 78 | Mar.BI3 | white | Jinzhai County, Anhui Province | 2020.3 |
| 79 | Mar.FM1 | brown | Jinzhai County, Anhui Province | 2020.3 |
| 80 | Mar.FM2 | brown | Jinzhai County, Anhui Province | 2020.3 |
| 81 | Mar.FM3 | brown | Jinzhai County, Anhui Province | 2020.3 |
| 82 | Apr.FP1 | dark brown | Jinzhai County, Anhui Province | 2020.4 |
| 83 | Apr.FP2 | dark brown | Jinzhai County, Anhui Province | 2020.4 |
| 84 | Apr.BO1 | white | Jinzhai County, Anhui Province | 2020.4 |
| 85 | Apr.BO2 | white | Jinzhai County, Anhui Province | 2020.4 |
| 86 | Apr.BI1 | white | Jinzhai County, Anhui Province | 2020.4 |
| 87 | Apr.BI2 | white | Jinzhai County, Anhui Province | 2020.4 |
| 88 | Apr.FM1 | brown | Jinzhai County, Anhui Province | 2020.4 |
| 89 | Apr.FM2 | brown | Jinzhai County, Anhui Province | 2020.4 |

Supplementary Table 3. Characterization of specialized metabolites of Fushen by UPLC-Q/TOF-MS in ESI^+^.

| No | *tR*  (min) | Molecular  Formula | Adduct ion(mass accuracy, ppm) | MS/MS Fragmentation(mass accuracy, ppm<10) | Identification | Ref. |
| --- | --- | --- | --- | --- | --- | --- |
| 14 | 2.96 | C_20_H_26_O_4_ | 331.1913[M+H]^+^(1.2) | 251.1639 | Unknown | Zhu et al., 2018 |
| 16 | 3.28 | C_30_H_46_O_5_ | 487.3432[M+H]^+^(1.8)  509.3219[M+Na]^+^ (-4.7) | 469.3271[M+H-H_2_O]^+^  451.3154 | Poricoic acid G | Zhu et al., 2018;  Zou et al., 2019 |
| 21 | 3.78 | C_31_H_48_O_5_ | 501.3552[M+H]+(-5.6) | 483.3493[M+H-H_2_O]^+^  465.3328[M+H-2H_2_O]^+^ | 29-Hydroxydehydrotumulosic acid | Zou et al., 2019 |
| 31 | 5.26 | C_31_H_46_O_5_ | 499.3413[M+H]^+^(-2.0)  521.3243[M+Na]^+^ (0.0) | 481.3316[M+H-H_2_O]^+^  463.3134  325.2158 | 25-Hydroxyporicoic acid C/25-Hydroxypolyporenic acid C/  6-Hydroxypolyporenic acid C/Poricoic acid BM/  3-Oxo-6,16α-dihydroxy-lanosta-7,9(11),24(31)-trien-21-oic acid | Li, 2013;  Wang, 2016;  Zhu et al., 2018 |
| 33 | 5.62 | C_31_H_46_O_5_ | 499.3413[M+H]^+^(-2.0)  521.3243[M+Na]^+^ (0.0) | 481.3316[M+H-H_2_O]^+^  463.3134  325.2158 | 25-Hydroxyporicoic acid C/25-Hydroxypolyporenic acid C/  6-Hydroxypolyporenic acid C/Poricoic acid BM/  3-Oxo-6,16α-dihydroxy-lanosta-7,9(11),24(31)-trien-21-oic acid | Li, 2013;  Wang, 2016;  Zhu et al., 2018 |
| 35 | 5.97 | C_30_H_44_O_4_ | 469.3315[M+H]^+^(-0.6)  491.3132[M+Na]^+^ (-1.0) | 451.3241[M+H-H_2_O]^+^  433.3111[M+H-2H_2_O]^+^ | 16-Deoxyporicoic acid B/  25-Methoxyporicoic acid A | Wang, 2016;  Zou et al., 2019 |
| 39* | 6.57 | C_30_H_46_O_4_ | 471.3462[M+H]^+^(-2.5)  493.3286[M+Na]^+^ (-1.6) | 453.3390[M+H-H_2_O]^+^  435.3281[M+H-2H_2_O]^+^  293.2283[M+H-2H_2_O-C_8_H_14_O_2_] ^+^ | 16α-Hydroxydehydrotrametenolic acid | Wang, 2016 |
| 40 | 6.81 | C_31_H_48_O_4_ | 485.3668[M+H]^+^(7.6) | 467.3477[M+H-H_2_O]^+^ | 3-epi-Dehydrotumulosic acid | Wang, 2016 |
| 41* | 6.96 | C_30_H_48_O_4_ | 473.3651[M+H]^+^(-1.1)  495.3440[M+Na]^+^ (-2.0) | 455.3540[M+H-H_2_O]^+^  437.3412[M+H-2H_2_O]^+^  295.2429[M+H-2H_2_O-C_8_H_14_O_2_] ^+^ | 16α-Hydroxytrametenolic acid | Zhu et al., 2018 |
| 42 | 7.16 | C_33_H_50_O_6_ | 543.3705[M+H]^+^(3.5) | 525.3593[M+H-H_2_O]^+^ | 6α-Hydroxydehydropachymic acid | Zhu et al., 2018 |
| 43 | 7.23 | C_31_H_46_O_4_ | 483.3493[M+H]^+^(3.9)  505.3307[M+Na]^+^ (2.6) | 465.3372[M+H-H_2_O]^+^  447.3251[M+H-2H_2_O]^+^ | Poricoic acid C | Zhu et al., 2018 |
| 45* | 7.56 | C_30_H_44_O_5_ | 485.3262[M+H]^+^(-1.0)  507.3105[M+Na]^+^ (3.7)  969.6479[2M+H]^+^(2.4) | 467.3167[M+H-H_2_O]^+^  449.3050[M+H-2H_2_O]^+^  325.2195[M+H-H_2_O-C_8_H_14_O_2_] ^+^ | Poricoic acid B | Zhu et al., 2018 |
| 47* | 7.89 | C_31_H_48_O_4_ | 485.3622[M+H]^+^(-1.9)  507.3474[M+Na]^+^ (4.7) | 467.3512[M+H-H_2_O]^+^  449.3441[M+H-2H_2_O]^+^  311.2366[M+H-H_2_O-C_9_H_16_O_2_] ^+^  293.2283[M+H-2H_2_O-C_9_H_16_O_2_] ^+^ | Dehydrotumulosic acid | Zhu et al., 2018 |
| 49 | 8.47 | C_31_H_50_O_4_ | 487.3794[M+H]^+^(1.4)  509.3589[M+Na]^+^ (-3.5) | 469.3669[M+H-H_2_O]^+^  451.3589[M+H-2H_2_O]^+^ | Tumulosic acid | Wang, 2016;  Zhu et al., 2018 |
| 51* | 9.00 | C_31_H_46_O_5_ | 499.3413[M+H]^+^(-2.0)  521.3243[M+Na]^+^(0.0)  997.6788[2M+H]^+^(1.9)  1019.6566[2M+Na]^+^(-2.2) | 481.3316[M+H-H_2_O]^+^  463.3222[M+H-2H_2_O]^+^  325.2158[M+H-H_2_O-C_9_H_16_O_2_]^+^  307.2067[M+H-2H_2_O-C_9_H_16_O_2_] ^+^ | Poricoic acid A | Zhu et al., 2018 |
| 52 | 9.20 | C_30_H_46_O_5_ | 487.3432[M+H]^+^(1.8)  509.3219[M+Na]^+^(-4.7) | 469.3315[M+H-H_2_O]^+^  451.3198[M+H-2H_2_O]^+^ | Poriacosone A/Poriacosone B | Zheng and Yang, 2008; Zou et al., 2019 |
| 53 | 9.43 | C_30_H_46_O_4_ | 471.3506[M+H]^+^(6.8) | 453.3346[M+H-H_2_O]^+^  435.3238[M+H-2H_2_O]^+^  301.2182[M+H-HCOOH-C_9_H_16_O_2_]^+^ | 15α-Hydroxy-3-oxolanosta-8,24-dien-21-oic acid | Zhu et al., 2018;  Zou et al., 2019 |
| 54 | 9.77 | C_33_H_52_O_6_ | 545.3851[M+H]^+^(1.7) | 527.3732[M+H-H_2_O]^+^  451.3589 | 25-Hydroxypachymic acid | Zhu et al., 2018 |
| 56* | 10.23 | C_31_H_46_O_4_ | 483.3448[M+H]^+^(-5.4)  505.3307[M+Na]^+^ (2.6)  987.6669[2M+ Na]^+^(-2.1) | 465.3372[M+H-H_2_O]^+^  447.3294[M+H-2H_2_O]^+^  309.2219 [M+H-H_2_O-C_9_H_16_O_2_]^+^ | Polyporenic acid C | Zhu et al., 2018 |
| 59 | 10.75 | C_31_H_48_O_4_ | 485.3622[M+H]^+^(-1.9) | 467.3521[M+H-H_2_O]^+^ | 15α-Hydroxyeburiconic acid | Zou et al., 2019 |
| 61 | 11.21 | C_31_H_48_O_4_ | 485.3622[M+H]^+^(-1.9)  507.3474[M+Na]^+^ (4.7) | 467.3521[M+H-H_2_O]^+^  449.3441[M+H-2H_2_O]^+^ | 16α-Hydroxyeburiconic acid | Zou et al., 2019 |
| 65 | 12.2 | C_31_H_50_O_4_ | 487.3749[M+H]^+^(-7.8)  509.3589[M+Na]^+^ (-3.5) | 469.3714 | Hispindic acid B | Zou et al., 2019 |
| 68 | 12.88 | C_20_H_28_O_2_ | 301.1436[M+H]^+^(-1.3) |  | Unknown |  |
| 75* | 14.24 | C_32_H_48_O_5_ | 513.3566[M+H]^+^(-2.7)  535.3396[M+Na]^+^ (-0.6)  1047.6874[2M+ Na]^+^(-2.6) | 495.3485[M+H-H_2_O]^+^  435.3281[M+H-CH_3_COOH-2H_2_O]^+^  353.2489[M+H-H_2_O-C_8_H_14_O_2_]^+^ | 3-O-Acetyl-16α-hydroxydehydrotrametenolic acid | Zhu et al., 2018;  Zou et al., 2019 |
| 77 | 14.60 | C_33_H_48_O_5_ | 525.3593[M+H]^+^(2.5) | 509.3635[M+H-H_2_O]^+^  447.3263[M+H-CH_3_COOH -H_2_O]^+^ | 16α-Acetoxypolyporenic acid C | Wang, 2016 |
| 80* | 15.41 | C_32_H_50_O_5_ | 515.3753[M+H]^+^(2.9)  537.3583[M+Na]^+^ (5.0)  1051.7247[2M+ Na]^+^(3.1) | 497.3635[M+H-H_2_O]^+^  437.3412[M+H-H_2_O-CH_3_COOH]^+^  295.2429[M+H-H_2_O-CH_3_COOH-C_8_H_14_O_2_]^+^ | 3-O-Acetyl-16α-hydroxytrametenolic acid | Wang, 2014;  Zhu et al., 2018 |
| 83* | 16.33 | C_33_H_50_O_5_ | 527.3732[M+H]^+^(-2.8)  549.3538[M+Na]^+^ (-3.3)  1075.7224[2M+ Na]^+^(0.9) | 509.3632[M+H-H_2_O]^+^  449.3441[M+H-H_2_O-CH_3_COOH]^+^  353.2489[M+H-H_2_O-C_9_H_16_O_2_]^+^  293.2283[M+H-H_2_O-C_9_H_16_O_2_-CH_3_COOH]^+^ | Dehydropachymic acid | Zhu et al., 2018 |
| 86* | 17.56 | C_33_H_52_O_5_ | 529.3910[M+H]^+^(3.2)  551.3699[M+Na]^+^ (-2.4)  1057.7720[2M+H] ^+^ (1.1)  1079.7528[2M+ Na]^+^(0.1) | 511.3789[M+H-H_2_O]^+^  469.3714[M+H-CH_3_COOH]^+^  295.2429[M+H-H_2_O-C_9_H_16_O_2_-CH_3_COOH]^+^ | Pachymic acid | Zhu et al., 2018 |
| 99* | 21.72 | C_30_H_46_O_3_ | 455.3540[M+H]^+^(3.3)  477.3356[M+Na]^+^ (2.3) | 437.3412[M+H-H_2_O]^+^  409.3481[M+H-HCOOH]^+^ | Dehydrotratrametenolic acid | Zhu et al., 2018 |
| 103 | 22.98 | C_30_H_48_O_3_ | 457.3691[M+H]^+^(2.0) | 439.3590[M+H-H_2_O]^+^ | Trametenolic acid/Oleanolic acid | Wang, 2016 |
| 104 | 23.37 | C_30_H_48_O_3_ | 457.3691[M+H]^+^(2.0) | 439.3590[M+H-H_2_O]^+^ | Trametenolic acid/Oleanolic acid | Zhu et al., 2018 |
| 106* | 24.43 | C_31_H_48_O_3_ | 469.3669[M+H]^+^(-2.8)  959.6830[2M+ Na]^+^(3.2) | 451.3589[M+H-H_2_O]^+^  311.2366[M+H-C_9_H_16_O_2_]^+^  293.2248[M+H-H_2_O-C_9_H_16_O_2_]^+^ | Dehydroeburicoic acid | Zhu et al., 2018 |
| 109* | 25.78 | C_31_H_50_O_3_ | 471.3861[M+H]^+^(4.9)  493.3695[M+Na]^+^ (7.5) | 453.3738[M+H-H_2_O]^+^  435.3665[M+H-2H_2_O]^+^ | Eburicoic acid | Zhu et al., 2018;  Wang, 2016 |

*: Identified by comparing with standard compounds.

Supplementary Table 4. Calibration curves, LOD, LOQ, precision, stability, repeatability and recovery of thirteen standard compounds.

| Compounds | Calibration curves | r | Linear range(mg/mL) | LODs  (mg/mL) | LOQs  (mg/mL) | Precision(n=RSD%,6)  Intraday Interday | | Stability  (n=RSD%,6) | Repeatability  (n=RSD%,6) | Recovery  (n=RSD%,9) |
| --- | --- | --- | --- | --- | --- | --- | --- | --- | --- | --- |
| 1 | y=2E+07X-1987 | 0.9998 | 0.0034-0.1360 | 0.0012 | 0.0034 | 0.59 | 2.94 | 1.29 | 0.73 | 98.79±1.70 |
| 2 | y=5E+06X-24363 | 0.9991 | 0.0140-0.2800 | 0.0056 | 0.0140 | 0.47 | 0.97 | 0.78 | 0.95 | 103.18±2.66 |
| 3 | y=1E+07X-33576 | 0.9998 | 0.0067-0.9000 | 0.0018 | 0.0067 | 0.27 | 2.82 | 1.32 | 1.71 | 97.54±3.34 |
| 4 | y=2E+07X-87844 | 0.9995 | 0.0092-0.9200 | 0.00368 | 0.0078 | 0.72 | 1.27 | 2.51 | 2.30 | 103.65±2.62 |
| 5 | y=9E+06X+12845 | 0.9997 | 0.0084-2.1000 | 0.00315 | 0.0080 | 0.36 | 1.47 | 1.58 | 1.32 | 102.40±2.23 |
| 6 | y=2E+07X-27410 | 0.9998 | 0.0055-0.4340 | 0.00124 | 0.0055 | 0.70 | 1.63 | 0.79 | 0.63 | 102.43±2.83 |
| 7 | y=1E+07X-21465 | 0.9997 | 0.0076-0.4500 | 0.0022 | 0.0076 | 1.12 | 2.70 | 2.23 | 1.72 | 97.41±3.26 |
| 8 | y=7E+06X-48063 | 0.9991 | 0.0360-0.2800 | 0.0112 | 0.0360 | 0.44 | 1.48 | 0.79 | 0.72 | 98.62±2.20 |
| 9 | y=1E+07X+33881 | 0.9998 | 0.0147-1.1800 | 0.0029 | 0.0073 | 0.58 | 1.12 | 0.16 | 0.17 | 98.08±1.32 |
| 10 | y=5E+06X-7429 | 0.9998 | 0.1200-1.2000 | 0.0430 | 0.1200 | 0.97 | 2.78 | 1.58 | 2.30 | 97.95±2.83 |
| 11 | y=2E+07X+134438 | 0.9997 | 0.0044-1.3350 | 0.0008 | 0.0044 | 1.48 | 1.44 | 0.98 | 0.42 | 102.53±1.78 |
| 12 | y=2E+07X+173915 | 0.9990 | 0.0051-1.3800 | 0.0009 | 0.0046 | 1.58 | 2.78 | 1.63 | 0.86 | 101.49±2.49 |
| 13 | y=8E+06X-42899 | 0.9990 | 0.1300-0.3000 | 0.0280 | 0.0675 | 1.20 | 1.72 | 0.79 | 2.50 | 96.38±2.74 |

1,2,3,4,5,6,7,8,9,10,11,12,13 were represented16α-hydroxydehydrotrametenolic acid, 16α-hydroxytrametenolic acid, poricoic acid B, dehydrotumulosic acid, poricoic acid A, polyporenic acid C, 3-o-acetyl-16α-hydroxydehydrotrametenolic acid, 3-o-acetyl-16α-hydroxytrame-tenolic acid, dehydropachymic acid, pachymic acid, dehydrotratrametenolic acid, dehydroeburicoic acid, eburicoic acid, respectively.

Supplementary Table 5. Potential markers for the differentiation of nine growth periods of Fushen.

| No | *tR*  (min) | Molecular  Formula | Adduct ion(mass accuracy, ppm) | MS/MS Fragmentation(mass accuracy, ppm<10) | Identification | Ref. |
| --- | --- | --- | --- | --- | --- | --- |
| 15 | 3.05 | C_30_H_44_O_6_ | 499.3052[M-H]^-^(-1.6)  545.3123[M-H+HCOOH]^-^(1.7) | 481.2930[M-H-H_2_O]^-^  425.2697[M-H-C_2_H_5_COOH]^-^  467.2764  327.2152 | Poricoic acid E/Poricoic acid E isomer | Feng et al., 2018;  Zhu et al., 2018 |
| 21 | 3.78 | C_31_H_48_O_5_ | 499.3418[M-H]^-^(-1.0)  545.3505[M-H+HCOOH]^-^(5.0)  999.6895[2M-H]^-^(-3.0) | 481.3334[M-H-H_2_O]^-^  419.2980[M-H-HCOOH-CH_4_-H_2_O]^-^  437.3315 | 29-Hydroxydehydrotumulosic acid | Zou et al., 2019 |
| 22 | 4.03 | C_31_H_46_O_6_ | 513.3223[M-H]^-^(1.4)  559.3224[M-H+HCOOH]^-^(5.0) | 439.2852[M-H-C_2_H_5_COOH]^-^  487.3416  441.2992 | 5α,3β-Peroxy-dehydrotumulosic acid/  5α,8α-Peroxy-dehydrotumulosic acid | Wang, 2014;  Zhu et al., 2018;  Zou et al., 2019 |
| 25 | 4.30 | C_30_H_48_O_5_ | 487.3416[M-H]^-^(-1.4)  533.3452[M-H+HCOOH]^-^(-4.9) | 451.3192[M-H-2H_2_O]^-^  441.2261  367.2089 | 3α,16α,25-Trihydroxylanosta-8,24-dien-21-oic acid | Zou et al., 2019 |
| 33 | 5.62 | C_31_H_46_O_5_ | 497.3270[M-H]^-^(0.6)  543.3356[M-H]^-^(6.3)  995.6573[2M-H]^-^(-3.9) | 483.3155  469.3313  467.3162 | 25-Hydroxyporicoic acid C/25-Hydroxypolyporenic acid C/  6-Hydroxypolyporenic acid C/Poricoic acid BM/  3-Oxo-6,16α-dihydroxy-lanosta-7,9(11),24(31)-trien-21-oic acid | Li, 2013;  Wang, 2016;  Zhu et al., 2018 |
| 35 | 5.97 | C_30_H_44_O_4_ | 467.3162[M-H]^-^(0.2)  513.3177[M-H+HCOOH]^-^(-7.6) | 407.2950[M-H-CO_2_-CH_4_]^-^  393.2802[M-H-C_2_H_5_COOH]^-^ | 16-Deoxyporicoic acid B/  25-Methoxyporicoic acid A | Wang, 2016;  Zou et al., 2019 |
| 36 | 6.11 | C_32_H_48_O_6_ | 527.3361[M-H]^-^(-2.3)  573.3436 [M-H+HCOOH]^-^(1.6) | 481.3065  419.1387 | Poricoic acid DM | Zhu et al., 2018 |
| 38 | 6.41 | C_31_H_50_O_4_ | 485.3603[M-H]^-^(-5.8)  531.3666[M-H+HCOOH]^-^(-3.8) | 469.3623  427.2726 | 3β-Hydroxy-25-methoxy-24-methylene-27-Norlanost-8-en-21-oic acid/3-Oxo-6,16α-dihydroxy-lanosta-8,24-dien-21-oic acid | Zhu et al., 2018  Wang, 2014 |
| 43 | 7.23 | C_31_H_46_O_4_ | 481.3334[M-H]^-^(3.3)  527.3361[M-H+HCOOH]^-^(-2.3) | 407.2930[M-H-C_2_H_5_COOH]^-^ | Poricoic acid C | Zhu et al., 2018 |
| 45* | 7.56 | C_30_H_44_O_5_ | 483.3110[M-H]^-^(0.0)  529.3212[M-H+HCOOH]^-^(8.9) 967.6332[2M-H]^-^(4.0) | 409.2718[M-H-C_2_H_5_COOH]^-^ | Poricoic acid B | Zhu et al., 2018 |
| 47* | 7.89 | C_31_H_48_O_4_ | 483.3470[M-H]^-^(-0.8)  529.3542[M-H+HCOOH]^-^(2.5)  967.7032[2M-H]^-^(0.5) | 439.3538[M-H-CO_2_]^-^  437.3400[M-H-HCOOH]^-^  421.3119[M-H-HCOOH-CH_4_]^-^  389.2847[M-H-H_2_O-CO_2_-2CH_4_]^-^  311.2015[M-H-C_9_H_16_O_2_-CH_4_]^-^ | Dehydrotumulosic acid | Zhu et al., 2018 |
| 48 | 8.20 | C_32_H_50_O_6_ | 529.3542[M-H]^-^(2.5) | 483.3470[M-H-HCOOH]^-^  469.3313[M-H-CO_2_-CH_4_]^-^ | 3β-Acetyloxy-16α,26-dihydroxy-Lanosta-8,24-dien-21-oic acid | Zhu et al., 2018 |
| 52 | 9.20 | C_30_H_46_O_5_ | 485.3242[M-H]^-^(-5.2)  531.3336[M-H+HCOOH]^-^(2.6) | 467.3162[M-H_2_O]^-^  441.3336[M-H-CO_2_]^-^  423.2927[M-H-HCOOH-CH_4_]^-^ | Poriacosone A or Poriacosone B | Zheng and Yang, 2008; Zou et al., 2019 |
| 54 | 9.77 | C_33_H_52_O_6_ | 543.3690[M-H]^-^(0.7)  589.3753[M-H+HCOOH]^-^(2.2) | 483.3425[M-H-CO_2_-CH_4_]^-^  481.3289[M-H-HCOOH-CH_4_]^-^  467.3516  437.3229 | 25-Hydroxypachymic acid | Zhu et al., 2018 |
| 57 | 10.48 | C_31_H_46_O_4_ | 481.3289[M-H]^-^(-6.0) | 421.3077[M-H-CO_2_-CH_4_]^-^  469.3313  325.1749 | Polyporenic acid C isomer |  |
| 58 | 10.62 | C_33_H_50_O_6_ | 541.3530[M-H]^-^(0.2) | 495.3474[M-H-HCOOH]^-^  481.3334[M-H-CH_3_COOH]^-^  293.2087 | 29-Hydroxydehydropachymic acid | Zou et al., 2019 |
| 60 | 10.94 | C_31_H_48_O_5_ | 499.3418[M-H]^-^(-1.0)  545.3458[M-H+HCOOH]^-^(-3.7) | 481.3334[M-H-H_2_O]^-^  485.3242 | 16α,29-Dihydroxyeburiconic acid | Zou et al., 2019 |
| 64 | 11.89 | C_31_H_48_O_4_ | 483.3425[M-H]^-^(-10.1)  529.3495[M-H+HCOOH]^-^(6.4) | 421.2951  407.2498 | Dehydrotumulosic acid isomer | Zou et al., 2019 |
| 65 | 12.2 | C_31_H_50_O_4_ | 485.3648[M-H]^-^(3.5) | 423.3264[M-H-HCOOH-CH_4_]^-^  295.2237 | Hispindic acid B | Zou et al., 2019 |
| 66 | 12.39 | C_32_H_48_O_5_ | 511.3444[M-H]^-^(4.1)  557.3446[M-H+HCOOH]^-^(-5.7) | 451.3209[M-H-CH_3_COOH]^-^  467.3162  465.3363 | 3β-Acetoxy-16α,26-dihydroxy-lanosta-8,24-dien-21-oic acid  /Poricoic acid AM | Wang, 2016;  Zhu et al., 2018;  Zou et al., 2019 |
| 67 | 12.59 | C_32_H_46_O_5_ | 509.3286[M-H]^-^(3.7)  555.3269[M-H+HCOOH]^-^(7.7) | 449.3056[M-H-CO_2_-CH_4_]^-^ | Unknown |  |
| 74 | 14.03 | C_33_H_52_O_5_ | 527.3737[M-H]^-^(0.2) | 511.3351  339.1989 | Pachymic acid isomer |  |
| 75* | 14.24 | C_32_H_48_O_5_ | 511.3397[M-H]^-^(-5.1)  557.3495[M-H+HCOOH]^-^(3.1) | 467.3516[M-H-CO_2_]^-^  465.3363[M-H-HCOOH]^-^  451.3209[M-H-CH_3_COOH]^-^  355.2295[M-H-C_9_H_16_O_2_]^-^ | 3-O-Acetyl-16α-hydroxydehydrotrametenolic acid | Zhu et al., 2018;  Zou et al., 2019 |
| 76 | 14.43 | C_33_H_50_O_5_ | 525.3594[M-H]^-^(2.7)  571.3607[M-H+HCOOH]^-^(-4.9) | 463.3166[M-H-HCOOH-CH_4_]^-^  449.3058[M-H-CH_3_COOH-CH_4_]^-^ | 3-epi-Dehydropachymic acid | Zou et al., 2019  Zhu et al., 2018 |
| 77 | 14.60 | C_33_H_48_O_5_ | 523.3444[M-H]^-^(4.0)  569.3471[M-H+HCOOH]^-^(-1.2) | 463.3210[M-H-CH_3_COOH]^-^ | 16α-Acetoxypolyporenic acid C | Wang, 2016 |
| 80* | 15.41 | C_32_H_50_O_5_ | 513.3594[M-H]^-^(2.7)  559.3660[M-H+HCOOH]^-^(4.5) | 453.3361[M-H-CH_3_COOH]^-^  451.3209[M-H-HCOOH-CH_4_]^-^ | 3-O-Acetyl-16α-hydroxytrametenolic acid | Wang, 2014;  Zhu et al., 2018 |
| 90 | 19.10 | C_30_H_44_O_3_ | 451.3209[M-H]^-^(-0.7) | 433.1260 | 16α-Hydroxy-lanosta-7,9(11),24-trien-21-oic acid | Wang, 2016 |
| 92 | 19.88 | C_25_H_42_O_9_ | 485.2791[M-H]^-^(8.2) | 453.3318 | Unknow | Zhu et al., 2018 |
| 101 | 22.46 | C_35_H_54_O_6_ | 569.3862[M-H]^-^(3.5)  615.3923[M-H+HCOOH]^-^(4.2) |  | 16-O-Acetylpachymic acid/  3β,15α-Bis (acetyloxy)- 24-dien-21-oic acid | Zou et al., 2019  Wang, 2016; |
| 104 | 23.37 | C_30_H_48_O_3_ | 455.3515[M-H]^-^(-2.2)  501.3605[M-H+HCOOH]^-^(5.0) | 437.3315 | Trametenolic acid/Oleanolic acid | Zhu et al., 2018 |
| 109* | 25.78 | C_31_H_50_O_3_ | 469.3668[M-H]^-^(-3.0)  515.3738[M-H]^-^(0.4) | 453.3492  373.2771  339.2668  279.2227 | Eburicoic acid | Zhu et al., 2018;  Wang, 2016 |
| 113 | 27.62 | C_31_H_46_O_3_ | 465.3363[M-H]^-^(-1.3)  511.3397[M-H+HCOOH]^-^(-5.1) |  | Unknown | Wang, 2016 |

*: Identified by comparing with standard compounds.

Supplementary Table 6. Potential markers for the differentiation of four parts of Fushen.

| No | *tR*  (min) | Molecular  Formula | Adduct ion(mass accuracy, ppm) | MS/MS Fragmentation(mass accuracy, ppm<10) | Identification | Ref. |
| --- | --- | --- | --- | --- | --- | --- |
| 2 | 1.67 | C_20_H_26_O_5_ | 345.1689[M-H]^-^(-3.8) |  | Unknown | Zhu et al., 2018 |
| 3 | 1.73 | C_20_H_30_O_5_ | 349.1995[M-H]^-^(-5.7)  395.2063[M-H+HCOOH]^-^(-1.8) | 265.1440 | Pregn-7-ene-2β,3α,15α,20(S)-tetrol | Deng et al., 2020 |
| 4 | 1.87 | C_20_H_32_O_5_ | 351.2140[M-H]^-^(-8.8)  397.2187[M-H+HCOOH]^-^(-9.8) | 317.1723 | Unknown |  |
| 5 | 1.97 | C_20_H_28_O_4_ | 331.1883[M-H]^-^(-7.9) |  | Unknown | Zhu et al., 2018 |
| 6 | 2.09 | C_20_H_28_O_4_ | 331.1883[M-H]^-^(-7.9) |  | Unknown | Zhu et al., 2018 |
| 7 | 2.20 | C_17_H_24_O_4_ | 291.1574[M-H]^-^(-7.6) |  | Unknown | Zhu et al., 2018 |
| 9 | 2.52 | C_20_H_28_O_4_ | 331.1883[M-H]^-^(-7.9) |  | Unknown | Zhu et al., 2018 |
| 11 | 2.65 | C_20_H_32_O_4_ | 335.2229[M-H]^-^(2.1) |  | Unknown |  |
| 12 | 2.75 | C_20_H_32_O_5_ | 351.2140[M-H]^-^(-8.8)  397.2187[M-H+HCOOH]^-^(-9.8) | 317.1723 | Unknown |  |
| 13 | 2.86 | C_20_H_28_O_4_ | 331.1883[M-H]^-^(-7.9) |  | Unknown | Zhu et al., 2018 |
| 14 | 2.96 | C_20_H_26_O_4_ | 329.1765[M-H]^-^(3.6) | 251.1639 | Unknown | Zhu et al., 2018 |
| 27 | 4.49 | C_31_H_46_O_5_ | 497.3270[M-H]^-^(0.6)  543.3356[M-H]^-^(6.3)  995.6573[2M-H]^-^(-3.9) | 481.2930[M-H-CH_4_]^-^  419.2980[M-H-H_2_O-CO_2_-CH_4_]^-^  435.3310[M-H_2_O-CO_2_]^-^ | 25-Hydroxyporicoic acid C/25-Hydroxypolyporenic acid C/  6-Hydroxypolyporenic acid C/Poricoic acid BM/  3-Oxo-6,16α-dihydroxy-lanosta-7,9(11),24(31)-trien-21-oic acid | Li, 2013;  Wang, 2016;  Zhu et al., 2018 |
| 28 | 4.83 | C_31_H_48_O_6_ | 515.3367[M-H]^-^(-1.2)  561.3425[M-H+HCOOH]^-^(-0.4)  1031.6835[2M-H]^-^(1.1) | 497.3270[M-H-H_2_O]^-^  499.3418  423.2843 | 25-Hydroxyporicoic acid H | Zhu et al., 2018 |
| 38 | 6.41 | C_31_H_50_O_4_ | 485.3603[M-H]^-^(-5.8)  531.3666[M-H+HCOOH]^-^(-3.8) | 469.3623  427.2726 | 3β-Hydroxy-25-methoxy-24-methylene-27-Norlanost-8-en-21-oic acid/3-Oxo-6,16α-dihydroxy-lanosta-8,24-dien-21-oic acid | Zhu et al., 2018  Wang, 2014 |
| 48 | 8.20 | C_32_H_50_O_6_ | 529.3542[M-H]^-^(2.5) | 483.3470[M-H-HCOOH]^-^  469.3313[M-H-CO_2_-CH_4_]^-^ | 3β-Acetyloxy-16α,26-dihydroxy-Lanosta-8,24-dien-21-oic acid | Zhu et al., 2018 |
| 49 | 8.47 | C_31_H_50_O_4_ | 485.3648[M-H]^-^(3.5)  531.3666[M-H+HCOOH]^-^(-3.8) 971.7372[2M-H]^-^(3.3) | 469.3313[M-H-CH_4_]^-^  423.3264[M-H-HCOOH-CH_4_]^-^  391.3001[M-H- H_2_O-CO_2_-2CH_4_]^-^ | Tumulosic acid | Wang, 2016;  Zhu et al., 2018 |
| 51* | 9.00 | C_31_H_46_O_5_ | 497.3270[M-H]^-^(0.6)  543.3308[M-H+HCOOH]^-^(-2.6) 995.6658[2M-H]^-^(4.6) | 479.3151[M-H-H_2_O]^-^  423.2885[M-H-C_2_H_5_COOH]^-^  379.2993[M-H-C_2_H_5_COOH-CO_2_]^-^  363.2688[M-H-C_2_H_5_COOH-CO_2_-CH_4_]^-^ | Poricoic acid A | Zhu et al., 2018 |
| 54 | 9.77 | C_33_H_52_O_6_ | 543.3690[M-H]^-^(0.7)  589.3753[M-H+HCOOH]^-^(2.2) | 483.3425[M-H-CO_2_-CH_4_]^-^  481.3289[M-H-HCOOH-CH_4_]^-^  467.3516  437.3229 | 25-Hydroxypachymic acid | Zhu et al., 2018 |
| 59 | 10.75 | C_31_H_48_O_4_ | 483.3470[M-H]^-^(0.8)  529.3495[M-H+HCOOH]^-^(6.4) | 439.3581[M-H-CO_2_]^-^  437.3400[M-H-HCOOH]^-^  423.3222[M-H-CO_2_-CH_4_]^-^  421.3077[M-H-HCOOH-CH_4_]^-^ | 15α-Hydroxyeburiconic acid | Zou et al., 2019 |
| 62 | 11.50 | C_33_H_52_O_6_ | 543.3642[M-H]^-^(-8.1) | 483.3425[M-H-CO_2_-CH_4_]^-^ | 25-Hydroxypachymic acid isomer | Zhu et al., 2018 |
| 68 | 12.88 | C_20_H_28_O_2_ | 299.1999[M-H]^-^(-4.0) |  | Unknown |  |
| 69 | 13.19 | C_26_H_56_O_11_ | 543.3738[M-H]^-^(-1.1) |  | Unknown |  |
| 71 | 13.48 | C_35_H_54_O_6_ | 569.3862[M-H]^-^(3.5) |  | 16-O-Acetylpachymic acid/  3β,15α-Bis(acetyloxy)-24-dien-21-oic acid | Wang, 2016;  Zou et al., 2019 |
| 72 | 13.58 | C_32_H_48_O_5_ | 511.3397[M-H]^-^(-5.1)  557.3446[M-H+HCOOH]^-^(-5.7) | 451.3078  353.2073  293.1737 | 3β-Acetoxy-16α,26-dihydroxy-lanosta-8,24-dien-21-oic acid  /Poricoic acid AM | Wang, 2016;  Zhu et al., 2018;  Zou et al., 2019 |
| 73 | 13.81 | C_32_H_48_O_5_ | 511.3397[M-H]^-^(-5.1)  557.3446[M-H+HCOOH]^-^(-5.7) | 451.3078  353.2073  293.1737 | 3β-Acetoxy-16α,26-dihydroxy-lanosta-8,24-dien-21-oic acid/  Poricoic acid AM | Wang, 2016;  Zhu et al., 2018;  Zou et al., 2019 |
| 75* | 14.24 | C_32_H_48_O_5_ | 511.3397[M-H]^-^(-5.1)  557.3495[M-H+HCOOH]^-^(3.1) | 467.3516[M-H-CO_2_]^-^  465.3363[M-H-HCOOH]^-^  451.3209[M-H-CH_3_COOH]^-^  355.2295[M-H-C_9_H_16_O_2_]^-^ | 3-O-Acetyl-16α-hydroxydehydrotrametenolic acid | Zhu et al., 2018;  Zou et al., 2019 |
| 76 | 14.43 | C_33_H_50_O_5_ | 525.3594[M-H]^-^(2.7)  571.3607[M-H+HCOOH]^-^(-4.9) | 463.3166[M-H-HCOOH-CH_4_]^-^  449.3058[M-H-CH_3_COOH-CH_4_]^-^ | 3-epi-Dehydropachymic acid | Zou et al., 2019  Zhu et al., 2018 |
| 80* | 15.41 | C_32_H_50_O_5_ | 513.3594[M-H]^-^(2.7)  559.3660[M-H+HCOOH]^-^(4.5) | 453.3361[M-H-CH_3_COOH]^-^  451.3209[M-H-HCOOH-CH_4_]^-^ | 3-O-Acetyl-16α-hydroxytrametenolic acid | Wang, 2014;  Zhu et al., 2018 |
| 86* | 17.56 | C_33_H_52_O_5_ | 527.3737[M-H]^-^(0.2)  573.3779[M-H+HCOOH]^-^(-2.1) 1055.7560[2M-H]^-^(0.9) | 481.3649[M-H-HCOOH]^-^  467.3516[M-CH_3_COOH]^-^  465.3363[M-H-HCOOH-CH_4_]^-^  339.1951[M-H-C_9_H_16_O_2_-2CH_4_]^-^ | Pachymic acid | Zhu et al., 2018 |
| 88 | 18.37 | C_29_H_46_O_5_ | 473.3311[M-H]^-^(9.3) |  | Unknown |  |
| 99* | 21.72 | C_30_H_46_O_3_ | 453.3361[M-H]^-^(-1.8)  499.3418[M-H-HCOOH]^-^(-1.0) 907.6812[2M-H]^-^(-0.4) | 435.3265[M-H_2_O]^-^ | Dehydrotrametenolic acid | Zhu et al., 2018 |
| 101 | 22.46 | C_35_H_54_O_6_ | 569.3862[M-H]^-^(3.5)  615.3923[M-H+HCOOH]^-^(4.2) |  | 16-O-Acetylpachymic acid/  3β,15α-Bis (acetyloxy)- 24-dien-21-oic acid | Zou et al., 2019  Wang, 2016; |
| 103 | 22.98 | C_30_H_48_O_3_ | 455.3515[M-H]^-^(-2.2)  501.3605[M-H+HCOOH]^-^(5.0) | 437.3315 | Trametenolic acid/Oleanolic acid | Wang, 2016 |
| 106* | 24.43 | C_31_H_48_O_3_ | 467.3516[M-H]^-^(-1.9)  513.3594[M-H-HCOOH]^-^(2.7) 935.7140[2M-H]^-^(1.2) | 371.2554,  337.2531,  339.2668  279.2256 | Dehydroeburicoic acid | Zhu et al., 2018 |
| 109* | 25.78 | C_31_H_50_O_3_ | 469.3668[M-H]^-^(-3.0)  515.3738[M-H]^-^(0.4) | 453.3492  373.2771  339.2668  279.2227 | Eburicoic acid | Zhu et al., 2018;  Wang, 2016 |
| 112 | 27.22 | C_31_H_46_O_3_ | 465.3363[M-H]^-^(-1.3)  511.3397[M-H+HCOOH]^-^(-5.1) |  | Unknown | Chen et al., 2019 |
| 114 | 28.09 | C_31_H_48_O_3_ | 467.3516[M-H]^-^(-1.9) |  | Dehydroeburicoic acid isomer |  |
| 117 | 29.23 | C_33_H_62_O_10_ | 617.4265[M-H]^-^(-2.9) |  | Unknown |  |
| 119 | 29.56 | C_32_H_48_O_4_ | 495.3483[M-H]^-^(1.8)  541.3482[M-H+HCOOH]^-^(-8.7) | 473.3612 | Poricoic acid CM | Zhu et al., 2018 |

*: Identified by comparing with standard compounds.
